# A comparative analysis employing a gene- and genome-centric metagenomic approach reveals changes in composition, function, and activity in waterworks with different treatment processes and source water in Finland

**DOI:** 10.1016/j.watres.2022.119495

**Published:** 2022-12-14

**Authors:** Vicente Gomez-Alvarez, Sallamaari Siponen, Ari Kauppinen, Anna-Maria Hokajärvi, Ananda Tiwari, Anniina Sarekoski, Ilkka T. Miettinen, Eila Torvinen, Tarja Pitkänen

**Affiliations:** a Office of Research and Development, U.S. Environmental Protection Agency, 26W. Martin Luther King Dr., Cincinnati, OH 45268, United States; b Finnish Institute for Health and Welfare, Department of Health Security, Kuopio 70701, Finland; c Department of Environmental and Biological Sciences, Kuopio 70211, Finland; d Faculty of Veterinary Medicine, Department Food Hygiene and Environmental Health, University of Helsinki, Helsinki 00790, Finland

**Keywords:** Drinking water distribution systems, Microbial community, Metagenome, Metatranscriptome, Metagenome-assembled genome, Microbiome

## Abstract

The emergence and development of next-generation sequencing technologies (NGS) has made the analysis of the water microbiome in drinking water distribution systems (DWDSs) more accessible and opened new perspectives in microbial ecology studies. The current study focused on the characterization of the water microbiome employing a gene- and genome-centric metagenomic approach to five waterworks in Finland with different raw water sources, treatment methods, and disinfectant. The microbial communities exhibit a distribution pattern of a few dominant taxa and a large representation of low-abundance bacterial species. Changes in the community structure may correspond to the presence or absence and type of disinfectant residual which indicates that these conditions exert selective pressure on the microbial community. The Archaea domain represented a small fraction (up to 2.5%) and seemed to be effectively controlled by the disinfection of water. Their role particularly in non-disinfected DWDS may be more important than previously considered. In general, non-disinfected DWDSs harbor higher microbial richness and maintaining disinfectant residual is significantly important for ensuring low microbial numbers and diversity. Metagenomic binning recovered 139 (138 bacterial and 1 archaeal) metagenome-assembled genomes (MAGs) that had *a* >50% completeness and <10% contamination consisting of 20 class representatives in 12 phyla. The presence and occurrence of nitrite-oxidizing bacteria (NOB)-like microorganisms have significant implications for nitrogen biotransformation in drinking water systems. The metabolic and functional complexity of the microbiome is evident in DWDSs ecosystems. A comparative analysis found a set of differentially abundant taxonomic groups and functional traits in the active community. The broader set of transcribed genes may indicate an active and diverse community regardless of the treatment methods applied to water. The results indicate a highly dynamic and diverse microbial community and confirm that every DWDS is unique, and the community reflects the selection pressures exerted at the community structure, but also at the levels of functional properties and metabolic potential.

## 1. Introduction

Despite considerable improvements in treatment processes and disinfection practices, outbreaks associated with drinking water have been consistently reported worldwide (WHO, 2021). As a result, effective monitoring of microbial contamination in drinking water distribution systems (DWDSs) is critical to reduce health risks, particularly for immunocompromised members of populations (Prüss-Üstün et al., 2008; Ashbolt, 2015; Yates, 2019). Culture-based methods are primarily used to assess the microbial quality of drinking water, but these assays are selective in nature, providing a limited view of relevant issues (Gomez-Alvarez et al., 2015), such as: (1) intrinsic microbial diversity within DWDSs; (2) metabolic potential that might enhance the survival of pathogens; (3) presence of antibiotic resistance genes and antimicrobial resistance mechanisms; and (4) microbes responsible for the deterioration of distribution system infrastructure and water quality. The implementation of next generation sequencing technology (NGS) to study the water microbiome has provided a better understanding of the taxonomic affiliation, functional potential, and overall microbial diversity in DWDSs (Tan et al., 2015; Zhang and Liu, 2019; Garner et al., 2021; Tiwari et al., 2022). More recently, advanced metagenomic surveys have documented that DWDSs support a complex microbial network (Douterelo et al., 2018; Brumfield et al., 2020; Gomez-Alvarez et al., 2021). Therefore, a more complete characterization of the microbial community structure of DWDSs using molecular tools is critical to address public health research questions (e.g., conditions promoting the emergence of pathogens), which may be more difficult to answer using traditional methods.

Most of the Finnish population (up to 90%) is served by centralized DWDSs with the rest of the population using an alternate source of drinking water such as private drinking water wells (Ikonen et al., 2017). In 2018, there were 153 large EU-regulated waterworks in Finland that met the reporting criteria of the Drinking Water Directive, which supplied domestic water to about 4.5 million users with 43% of domestic water produced from groundwater, 38% from surface water and the remaining 19% from artificially recharged groundwater (Zacheus, 2013). The disinfection methods in Finnish waterworks include non-disinfection and combinations of UV-light, ClO_2_, Cl, NH_2_Cl and NaOCl in other systems. Since water source, treatment processes and disinfectant play a key role in shaping the bacterial community in the distribution system, we hypothesize that the bulk water at each service area will harbor distinct and diverse bacterial communities as well as unique water functional potential. This study reports on the characterization of the bulk phase water microbial community, based on 16S rRNA gene, metagenome and metatranscriptome libraries, from five waterworks in Finland with different raw water sources, treatment methods, and disinfectant. This study provides information regarding microbial community diversity influenced by different raw water sources and different water treatment technologies. It also provides a window into the functional properties and metabolic potential of the water microbiome.

## 2. Material and methods

### 2.1. DWDS sites, sample collection and nucleic acid extraction

A detailed description of the sample locations, details, and processing as well as specific details and characteristics of the five drinking water distribution systems (DWDS) can be found in our previous research papers focusing on the water microbiome (Inkinen et al., 2019, 2021) and the physico-chemical characteristics of DWDSs (Ikonen et al., 2017). The flowchart methodology of this research is available in Supplementary file: Fig. S1. Briefly, bulk water samples were collected from five different DWDSs (A to E) with the aim to cover the main treatment processes that are used in drinking water production in Finland (Table S1). Each waterwork varies regarding raw water source, treatment method, and disinfection treatment as follows: artificial groundwater production without any disinfection (DWDSs A and B, same geographical location), surface water with chlorine dioxide (ClO_2_) and chlorine (Cl_2_) disinfection (DWDS C), surface water with chloramine (NH_2_Cl) disinfection (DWDS D), and groundwater treated with sodium hypochlorite (NaOCl) (DWDS E). DWDS C to E included UV disinfection before chlorination.

Large volume bulk water samples (*n* = 10) were collected in two consecutive weeks during the summer season (August-September) at each location. The measured physico-chemical water quality and nutrients are available in Supplementary file: Table S2. Briefly, 100 L from the cold-water system was collected and filtered on-site using a dead-end ultrafiltration method (DEUF) using a Rexeed-25A hollow-fiber polysulfone filter (Asahi Kasei Medical Co., Ltd., Tokyo, Japan) attached to a tap (Inkinen et al., 2021). The average flow of water during sample collection was 3 L/min. The filters were stored and transported on ice for elution and DNA/RNA processing in the laboratory. Detailed information on sample collection, nucleic acid extraction, and complementary DNA (cDNA) synthesis from the purified RNA can be found in Supplementary file: Materials and Methods.

### 2.2. 16S rRNA gene amplification, sequencing, and reads processing

The highly variable V3–4 region of the bacteria 16S rRNA gene was amplified using the primer set 341F and 785R (Klindworth et al., 2013). The primers A340F (Gantner et al., 2011) and 915R (Stahl and Amann, 1991) were used for the amplification of the archaea 16S rRNA gene. Paired-end 300 bp reads were generated using the MiSeq^®^ platform (Illumina Inc., San Diego, USA) and screened following the procedure described in Gomez-Alvarez et al. (2016). Detailed information on PCR amplification, sequencing, and processing of reads can be found in Supplementary file: Materials and Methods. After quality control filtering and removal of artificial sequences, 138,073 and 54,534 reads were retained from 16S rRNA bacteria and archaea libraries, respectively. Archaea samples from sites C and D were excluded from further analysis due to a small number of recoverable reads.

### 2.3. 16S rRNA microbial community assemblages

Prior to community analysis, 16S rRNA gene libraries were rarefied to the smallest data set (4450 bacteria and 670 archaea reads). Bacteria and archaea analysis identified 6776 and 1176 operational taxonomic units (OTUs), respectively. Normalized libraries were used to calculate richness (*S*), richness estimators (ChaoI and *S*_ACE_), Shannon diversity (*H*) and evenness (*H*_*E*_) with the software mothur v1.45.2 (Schloss et al., 2009).

Taxonomic classification was obtained using the reference database Genome Taxonomy Database (GTDB) release 95 (Parks et al., 2020). Phylogenetic trees were constructed from the alignments of 16S rRNA gene sequences based on the maximum likelihood method using the software MEGA X v10.1.7 (Kumar et al., 2018).

### 2.4. Sequencing, processing, and assembly of metagenome and metatranscriptome libraries

Paired-end standard metagenome libraries were prepared using the Illumina HiSeq in the NovaSeq 6000 S2 PE150 XP sequence mode. Libraries were quality-checked using a 2100 Bioanalyzer Instrument (Agilent, Santa Clara, USA). Prior to assembly, the 150-nucleotide (nt) pair-end reads were subjected to quality filtering and cleaning from adapters and phiX artifacts, error corrected, normalized (≤100×), and filtered to a minimum length of 100-nt using the bioinformatics software package BBMap v38.22 (http://sourceforge.net/projects/bbmap) with the following parameters: ktrim=*r, k* = 23, mink=11, hdist=1, tbo, tpe, maxns=0, trimq=10, qtrim=*r*, maq=12, minlength=100, ecco=*t*, eccc=*t*, ecct=*t*, and target=100. The libraries contained an average (±SD) of 35,428,802 ± 3956,065 reads per sample.

To recover the active functional and metabolic information from the RNA extracted from the water samples, metatranscriptome of cDNA from the purified RNA were sequenced and processed following the same procedure as for the metagenome libraries. Then, ribosomal RNA was removed *in silico* by mapping metatranscriptomic reads to multiple rRNA databases with default settings using the local alignment tool SortMeRNA (Kopylova et al., 2012). The libraries from Sample B2, and DWDS C and E were excluded from subsequent analysis due to poor recovery of filtered reads. Only the data sets from sites A (ND) and D (CHM) were used to determine which populations were metabolically active at the time of sampling. The libraries contained an average (±SD) of 4290,346 ± 316,333 reads per sample.

### 2.5. Assembly, annotation, and OTU diversity of metagenomic reads

Libraries were *de novo* assembled using MEGAHIT v1.2.9 (Li et al., 2016) with default parameters but discarding contigs below 1500 nucleotides. Contigs were annotated with MetaProkka v1.14.6_1 (Telatin, 2020) a modified version of Prokka (Seemann, 2014). The tool SingleM v0.13.2 (Woodcroft, 2020) was used to estimate the abundances of OTUs directly from metagenome data from each sample. Shannon diversity was calculated based on the average of the rarefied OTU table across each of the 14 single-copy marker genes.

### 2.6. Taxonomic and metabolic inference of metagenomic and metatranscriptomic reads

Taxonomy was assigned using Kraken2 v2.1.2 (Wood et al., 2019) with a confidence value of 0.05 for taxonomic assignment using the pre-built custom Genome Taxonomy Database (GTDB) release 95 (http://ftp.tue.mpg.de/ebio/projects/struo2/GTDB_release95/kraken2). Taxonomy counts for each sample were summarized by collapsing taxonomic assignments to the phylum, class, order, family, and genus level with Bracken v.2.6.1 (Lu et al., 2017). A Sankey diagram was created using the online tool Pavian to illustrate the flow of reads from the root of the taxonomy to more specific ranks (Breitwieser et al., 2020). Metabolic reconstruction and the relative abundance of genes involved in key biogeochemical pathways were determined by DiTing v0.9 (Xue et al., 2021). Hierarchical classification (BRITE, KO, modules, pathways) of metabolic functions was obtained using the online tool FuncTree2 (Darzi et al., 2019). Selected waterborne pathogens were identified at the genus level from samples using the Pathogen-fluctuations script (Ghosh, 2021). The input file was the output of a UniRef90 (Suzek et al., 2015) based functional gene classification implemented through the HUMAnN v3.0.0 pipeline (Beghini et al., 2021). Detailed information on metabolic profiles and characterization can be found in Supplementary file: Materials and Methods.

### 2.7. Metagenomic binning

Prior to assembly, libraries were pooled (by location), and *de novo* co-assembly was performed with filtered reads using the assembler MEGAHIT. Contigs data was binned using anvi’o v6.1 (Eren et al., 2015) with the tools MaxBin2 v.2.2.6 (Wu et al., 2016) and MetaBat2 v.2.15 (Kang et al., 2019). Bins were optimized and dereplicated using the tool DAS Tool v 1.1.2 (Sieber et al., 2018). Subsequently, the bins were consolidated using MetaWRAP v1.3.2 (Uritskiy et al., 2018). Clusters were manually refined, and contaminants removed using the tools RefineM v0.1.1 (Parks et al., 2017) and MAGpurify v2.1.2 (Nayfach et al., 2019). Bins were reassembled with MetaWRAP (Uritskiy et al., 2018). The quality, coverage, and relative abundance in the community of metagenome-assembled genomes (MAGs) was assessed with CheckM v1.1.2 (Parks et al., 2015), and bins with ≥50% completeness, ≤10% contamination, and ≤10% strain heterogeneity were selected for downstream analysis (Bowers et al., 2017). MAGs were annotated with Prokka (Seemann, 2014), and their taxonomy was refined and confirmed by using GTDB-Tk v1.4.1 (Chaumeil et al., 2019) based on the GTDB release 95 database (Parks et al., 2020). Bins were de-replicated using dRep v3.2.0 (Olm et al., 2017) with default parameters. A phylogenetic tree of de-replicated MAGs was created with PhyloPhlAn v3.0.2 (Asnicar et al., 2020) using RAxML version 8.2.12 (Stamatakis et al., 2014). The tree was visualized with FigTree v1.4.4 (Rambaut, 2018). Metabolic reconstruction of each MAG was performed with the module METABOLIC–C using the software METABOLIC v4.0 (Zhou et al., 2020) with the following parameters: in-gn, r, rt=metaG and taxa=order. A Sankey diagram was created using the online tool SankeyMATIC (https://sankeymatic.com/) to illustrate the metabolic energy flow potential. Detailed information on MAG assemblies and characterization can be found in Supplementary file: Materials and Methods.

### 2.8. Multivariable ordination and statistical analysis

Non-metric multidimensional scaling (nMDS) was used to describe the relationships among microbial communities. The nMDS was based on the Square Root Jensen-Shannon Divergence coefficient (dissimilarity) matrix. The Jensen-Shannon divergence is a method of measuring the similarity between two probability distributions based on relative abundance. A one-way permutational multivariate analysis of variance (PERMANOVA) test was applied on the distance matrix with 9999 permutations to determine if there were significant differences (α = 0.05) between the microbial communities (Anderson, 2001). Similarity Percentage (SIMPER) analysis was conducted to determine the percentage contribution of species to the differences observed in non-disinfected to treated waters (Clarke, 1993). A Mann-Whitney U test (α = 0.05) was used to evaluate the differences in diversity indices (non-disinfected vs. treated waters), whereas the relationship between metabolic processes (e.g., genes) and DWDSs were examined using the non-parametric Kruskal-Wallis test for equal medians (α = 0.05). Ordination plots, PERMANOVA, SIMPER, Mann-Whitney U test, and Kruskal-Wallis analysis was performed with the software PAST v4.06 (Hammer et al., 2001). Statistical comparisons between total and active community profiles were calculated based on the Fisher’s exact test with corrected *q*-values (Storey’s FDR multiple test correction approach) using the software package STAMP v2.1.3 (Parks et al., 2010).

### 2.9. Data availability

The sequence data for this study have been deposited in the European Nucleotide Archive (ENA) at EMBL-EBI under accession number PRJEB40814 with the following BioSample numbers: SAMEA7465213 (Accession: ERS5222917) to SAMEA7465227 (ERS5222931). A single zip folder (THL_MAGs_selected_bins.zip) including all the selected MAGs is provided as a supplementary file.

## 3. Results and discussion

### 3.1. Microbial community assemblages were associated with disinfectant treatment

**Fig. 1.**
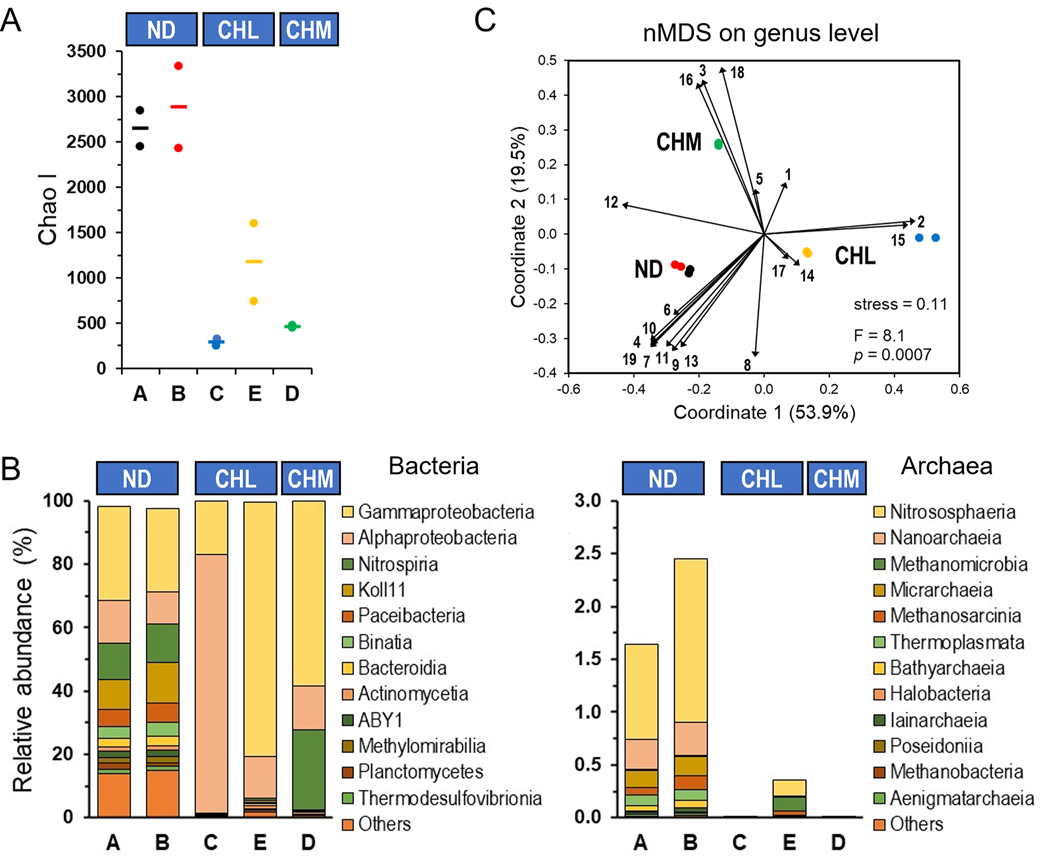
Community composition profiles of five DWDS revealed by a gene-centric metagenomic approach. (A) A decreasing gradient in microbial diversity revealed by Chao 1 from non-disinfected to treated water. (B) Taxonomic distribution of bacteria and archaea at the class level. (C) nMDS of all samples based on Jensen- Shannon distance on the genus level. Contribution of classes that explained ≈90% (SIMPER analysis) of the dissimilarity within all samples are represent by the size and direction of vectors. Numbers 1 to 19 indicate the classes *Gammaproteobacteria, Alphaproteobacteria, Nitrospiria, Paceibacteria, Actinomycetia, Bacteroidia, Binatia, Planctomycetes, Methylomirabilia, Thermodesulfovibrionia, Nitrososphaeria* (archaea), *Phycisphaerae, Verrucomicrobiae, Desulfuromonadia, Vampirovibrionia, Bdellovibrionia, Methanomicrobia* (archaea), *Zetaproteobacteria*, and *Koll11*, respectively. Disinfectant: ND, none; CHL, chlorine; CHM, chloramine.

Large volume bulk water samples were collected in two consecutive weeks during the summer season from each of the five DWDS locations. Raw water source and disinfection were the main differences between the systems (Ikonen et al., 2017). Metagenomic-based microbial diversity (Chao I index) was significantly higher in DWDSs (Mann-Whitney U test: *z* = 2.5, *p* = 0.0142) with no disinfectant (median: 2766, range from 2434 to 3334, *n* = 4) and decreased in chlorine and chloramine disinfected DWDSs (median: 641, range from 245 to 1601, *n* = 6) (Fig. 1A). The absence and decrease in disinfectant residual levels in conjunction with increasing distance from the waterworks enables regrowth and explains the increase in microbial growth at these distribution networks (Ikonen et al., 2017). Inkinen et al. (2019) observed a lower number of eukaryotic species in disinfected DWDSs as compared to nondisinfected water. Furthermore, Dai et al. (2020) established that the relative abundance of Archaea is dependent on the concentration of disinfectant residual. Maintaining disinfectant residual is significantly important for the mitigation of microbial contamination in DWDSs (USEPA, 2007). Although a few European countries require all water supplies to be disinfected and a disinfectant residual to be maintained, some countries do not require disinfection or the use of a disinfectant residual (Hydes, 1999). Finland offers guidance on disinfectant residuals (USEPA, 2007).

The taxonomic composition of the metagenomes revealed that most of the Bacteria domain diversity was associated with the phylum *Proteobacteria* followed by, *Nitrospirota, Omnitrophota, Patescibacteria, Planctomycetota, Bacteroidota, Desulfobacterota*, and *Actinobacteriota*. The Archaea domain represent a small fraction (up to 2.5%) of the prokaryote community (Fig. 1B). Results from previous shotgun metagenomic DNA sequencing studies have indicated lower proportions of archaeal representatives in drinking water systems (Gomez-Alvarez et al., 2012a and 2021; Douterelo et al., 2018). Although the abundance of archaeal representatives is relatively low, our study confirmed the presence of a highly taxonomic diversity composed of at least twelve major classes (Fig. 1B). Their role, particularly in non-disinfected DWDS may be more important than previously considered (Inkinen et al., 2021). The negative relationship between archaea abundance and disinfectant residual (i.e., treatment) is particularly evident for disinfected DWDSs, where the archaea community seemed to be effectively controlled by disinfection of the water. Overall, the observed taxonomic composition in which the dominant phyla represented here (albeit not at the same ratios) and the small relative abundance of Archaea is consistent with a previous meta-analysis of DWDSs (Bautista-de los Santos et al., 2016).

Moreover, the non-metric multidimensional scaling (nMDS) analysis formed three defined clusters (nMDS: stress = 0.11; PERMANOVA: *F* = 8.1, *p* = 0.0007) based on disinfectant treatment (Fig. 1C). The community composition in the DWDSs shifted markedly with disinfectant (disinfectant: none, ND; chlorine, CHL; chloramine, CHM): the dominant class switched from *Koll11, Paceibacteria, Binatia, Bacteroidia, Methylomirabilia, Thermodesulfovibrionia, Planctomycetes, Actinomycetia, Nitrososphaeria* (archaea), *Phycisphaerae*, and *Verrucomicrobiae* in ND communities to *Alphaproteobacteria, Desulfuromonadia, Vampirovibrionia*, and *Methanomicrobia* (archaea) when CHL was used as disinfectant. The DWDS treated with CHM was dominated by *Gammaproteobacteria, Nitrospiria, Bdellovibrionia*, and *Zetaproteobacteria*. This dissimilarity is explained by a small number of genus-level taxa (39 out of 3368 representing 1.2% of the taxa) whose relative abundance varied significantly among the disinfectant treatment (PERMANOVA: *F* = 8.1, *p* = 0.0007). The 21 taxa are among the most abundant taxa (each representing >1% of the total distribution) and explained 69% (Similarity Percentage [SIMPER] analysis) of the dissimilarity within DWDSs. The dominant taxa (in the following order of contribution) in ND DWDSs were closely related to members of the genera UBA10183 (class Koll11), UBA9968 (class *Binatia*), *Polynucleobacter, Immundisolibacter, Gallionella*, UBA6249 (class Koll11), UBA1546 (class *Thermodesulfovibrionia*), CG2–30–66–27 (phylum *Desulfobacterota*), RBG-16–66–20 (order *Burkholderiales*), UBA9973 (class *Paceibacteria*), UBA5619 (order *Syntrophales*), GWC2–42–12 (phylum *Patescibacteria*), and SXKJ01 (family *Planctomycetaceae*) (Figs. S2 and S3). Representatives of the genera UBA4765 (order *Rhizobiales*), *Hyphomicrobium, Limnohabitans, Polaromonas, Acidovorax, Thiobacillus, Rhodoferax, Sphingobium, Alicycliphilus, Giesbergeria, Hydrogenophaga, Bosea, Pseudomonas*, and *Sphingopyxis* were overrepresented in the CHL DWDSs (Figs. S4 and S5). The CHM DWDSs were dominated by the genera *Nitrosomonas, Nitrotoga, Nitrospira*, Palsa-1315 (family *Nitrospiraceae*), *Sphingomonas, Hylemonella*, PHCI01 (family *Burkholderiaceae*), *Aquabacterium, Bradyrhizobium*, and *Reyranella* (Fig. S6). These bacterial populations are ubiquitous members of DWDSs and may be considered part of the core microbiota of these ecosystems.

Concurrently we used 16S rRNA-based analysis of the Bacteria and Archaea domain to confirm that DWDSs with no disinfectant harbor higher microbial diversity (Figs. S7A and S8A). The resulting analysis of OTUs corroborated that the DWDS communities displayed variations in their taxonomic composition (Figs. S7B and S8B) and that bacterial communities formed clear clusters (nMDS: stress = 0.07; PERMANOVA: *F* = 5.8, *p* = 0.0007) based on disinfectant treatment (Fig. S7C). In general, complex microbial communities are extremely diverse and typically exhibit a distribution pattern of a few dominant taxa and a large representation of low-abundance bacterial species (Nemergut et al., 2013). Furthermore, the observed changes in the structure of the microbial community may correspond to the presence/absence and type of disinfectant residual which indicates that these conditions exert selective pressure on the microbial community.

### 3.2. Metabolic versatility of drinking water microbial communities

**Fig. 2.**
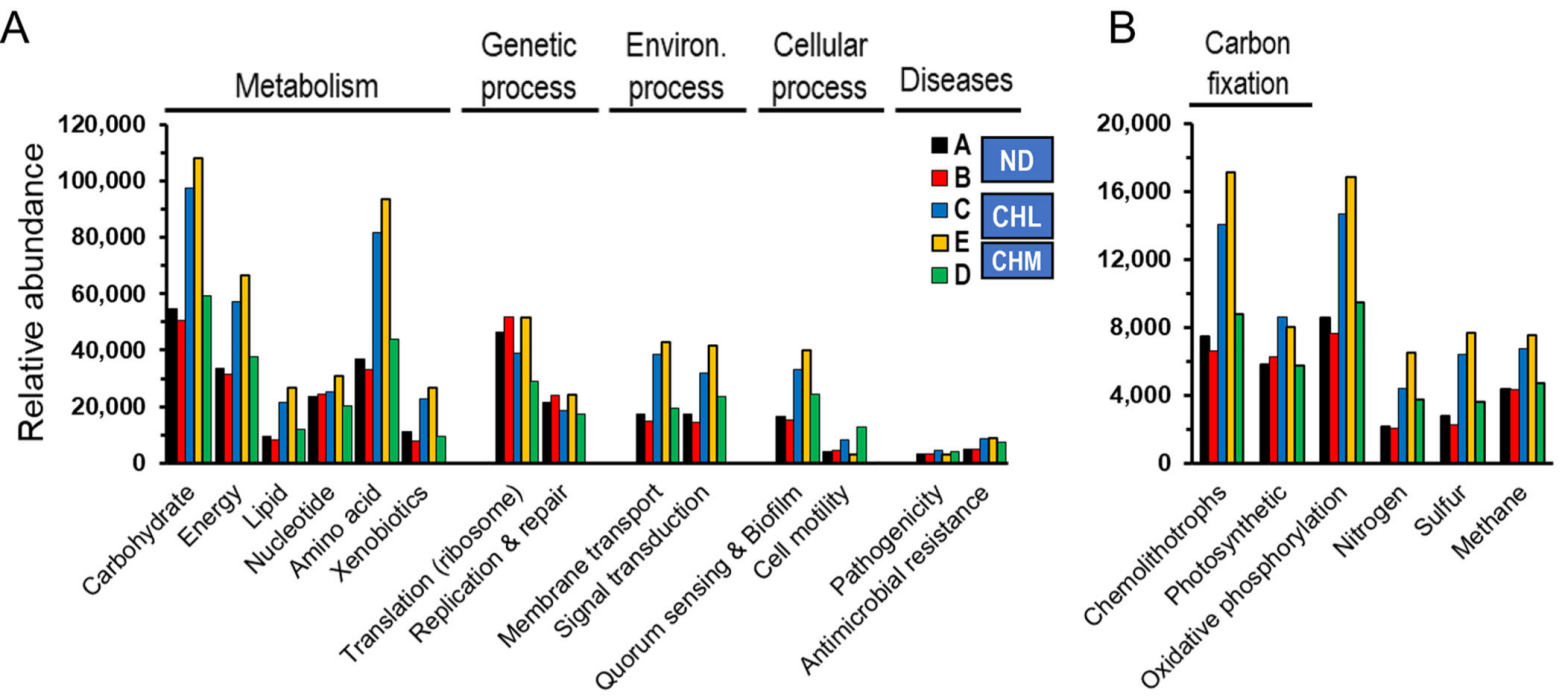
Metabolic potential of five DWDSs revealed by a gene-centric metagenomic approach. Relative abundance of KEGG orthologs (KOs) in (A) biological processes and (B) energy metabolic pathways. Disinfectant: ND, none; CHL, chlorine; CHM, chloramine.

We analyzed the metabolic potential of the DWDS metagenomes by focusing on KEGG-based pathways and modules (functional units of genes linked to specific metabolic capacities). The analysis of the metabolic potential revealed a total of 2990 KEGG orthologs (KOs) (ND = 2515; CHL = 2542; CHM = 2334) which were further categorized into 45 BRITE (level 2) functional categories (ND = 44; CHL = 44; CHM =45) comprising 344 pathways (ND = 307; CHL = 311; CHM = 326), and 778 modules (ND = 688; CHL = 701; CHM = 708). Drinking water systems host a great microbial biodiversity and provide niche stability to a vast collection of microorganisms, and our study detected and identified numerous functional processes comparable in numbers to complex ecosystems (Gomez-Alvarez et al., 2012a). These drinking water environments exhibit conditions that are favorable for the establishment of distinct communities harboring numerous biochemical processes (Fig. 2). Furthermore, the metagenomic analysis highlighted a moderate to high coverage (i.e., complete) of constructed KEGG pathways associated with core functions such as metabolism (energy, nucleotide, amino acid, xenobiotics), genetic processes (transcription, translation, replication, and repair), environmental processes (membrane transport, signal transduction), and cellular processes (growth, membrane functions) (Fig. S9). Among the energy metabolic processes (Fig. 2B), carbon fixation (chemolithotrophs and photosynthetic), oxidative phosphorylation, nitrogen, sulfur, and methane metabolisms showed a greater estimated number of completed pathways (avg coverage [±SD] = 79.4 ± 16.1) suggesting the potential of the water microbiome to actively utilize these energetic processes (Fig. S9B).

Functional analysis indicated differences in the metabolic characteristics of each DWDS at the KEGG pathways, modules, and KO levels. Cluster analysis grouped metabolic profiles (i.e., microbial communities) into three distinct clusters (PERMANOVA: *F* = 11.9, *p* = 0.0007) (Fig. S10). Metabolic processes as well as sequences associated with diseases, environmental, and cellular information processing were enriched in disinfected drinking water systems (CHL and CHM) compared to non-disinfected samples (Fig. 2A). In particular, the energic metabolic pathways constituting cellular respiration (oxidative phosphorylation) and carbon fixation (photosynthetic and non-photosynthetic [e.g., chemolithotrophs]), as well as metabolic pathways for nitrogen, sulfur, and methane were overrepresented in disinfected systems (Fig. 2B). The ecosystem characteristics of each DWDS may have led to differences in the ability to use carbon and nitrogen sources by their respective microbiome. These results, however, suggested that a higher taxonomic diversity did not accompany higher metabolic gene diversity (Figs. 1A and 2). For example, metabolic diversity was not significantly different between no disinfectant and disinfected DWDSs (Mann-Whitney U test: *z* = 1.2, *p* = 0.24095). The inconsistency between the metabolic and taxonomic diversities might suggest a higher functional redundancy of the microbial community in non-disinfected (ND) systems. Distinct species in similar niches might perform similar metabolic roles in biogeochemical cycles and overlapping niches may increase the functional redundancy of the ecosystem (Wang et al., 2017). In general, this study provided insight into the effects of disinfectant (as selective pressure) and water source on the metabolic potential of the DWDS microbial community. Similarly, Dai et al. (2020) suggests that selection pressures exerted within disinfected systems are not only evident at the community structure, but also evident at the community metabolic potential level.

### 3.3. Prevalence of antimicrobial resistance and occurrence of pathogenicity traits in DWDSs

Despite the drinking water in Finland being regularly monitored and showing excellent water quality, several antimicrobial resistance (AMR) and pathogenicity traits were identified in the DWDS metagenomes (Fig. 2A). The average proportion of genes associated with arsenic resistance (ars operons) was higher in the microbial communities in the CHL and CHM systems compared to ND water systems. A parallel study of these DWDSs revealed that most of the ARGs were associated with resistance to several antibiotic classes such as bacitracin, mupirocin, tetracycline, polymyxin, beta-lactam, aminoglycoside, glycopeptide, fosmidomycin, and fluoroquinolone (Tiwari et al., 2022). The DWDSs studied herein are considered pathogen-free but still might contain opportunistic waterborne pathogens. Preliminary results using the Pathogen-fluctuations script (Ghosh, 2021) identified functional genes of selected waterborne pathogens that are representatives of the genera *Acinetobacter, Aeromonas, Burkholderia, Campylobacter, Citrobacter, Enterobacter, Enterococcus, Escherichia, Haemophilus, Helicobacter, Klebsiella, Legionella, Mycobacterium, Proteus, Providencia, Pseudomonas, Salmonella, Serratia, Shigella, Staphylococcus, Stenotrophomonas, Streptococcus*, and *Vibrio*. These approaches only identified the presence of genes associated with resistance mechanisms and pathogenicity traits, and not whether these are actively transcribed genes, or whether their associated hosts are active members of the water microbiome at these sites.

The presence of these or other genes associated with AMR mechanisms and opportunistic waterborne pathogens does not imply evidence of water contamination or represent a risk to the public health. Nonetheless, these results may illustrate the ubiquity of these genes and public health-relevant or closely relate microorganisms in manufactured water systems (Sanganyado et al., 2019; Gao et al., 2021). The establishment and growth of waterborne pathogens (including resistance mechanisms to disinfectants and antibiotics) in drinking water systems may have significant impacts on human health as well as serious economic consequences.

### 3.4. Microbiome-level differences in biogeochemical processes between non-disinfected and disinfected systems

**Fig. 3.**
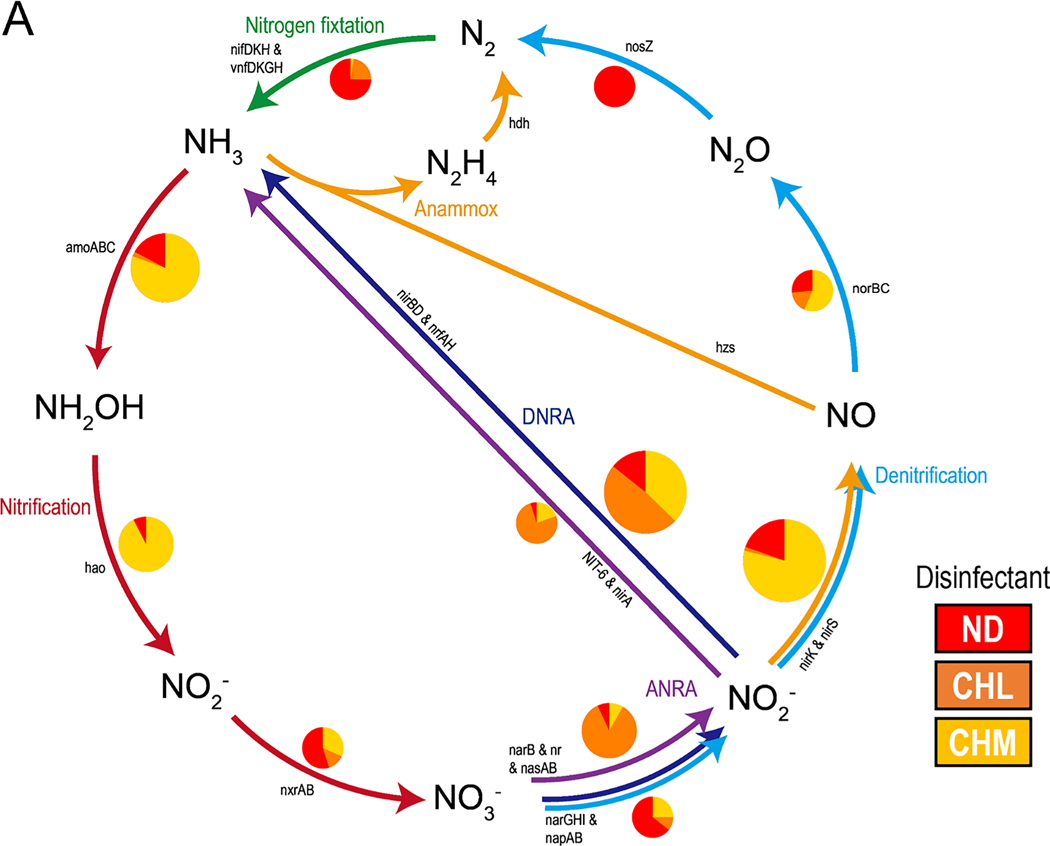

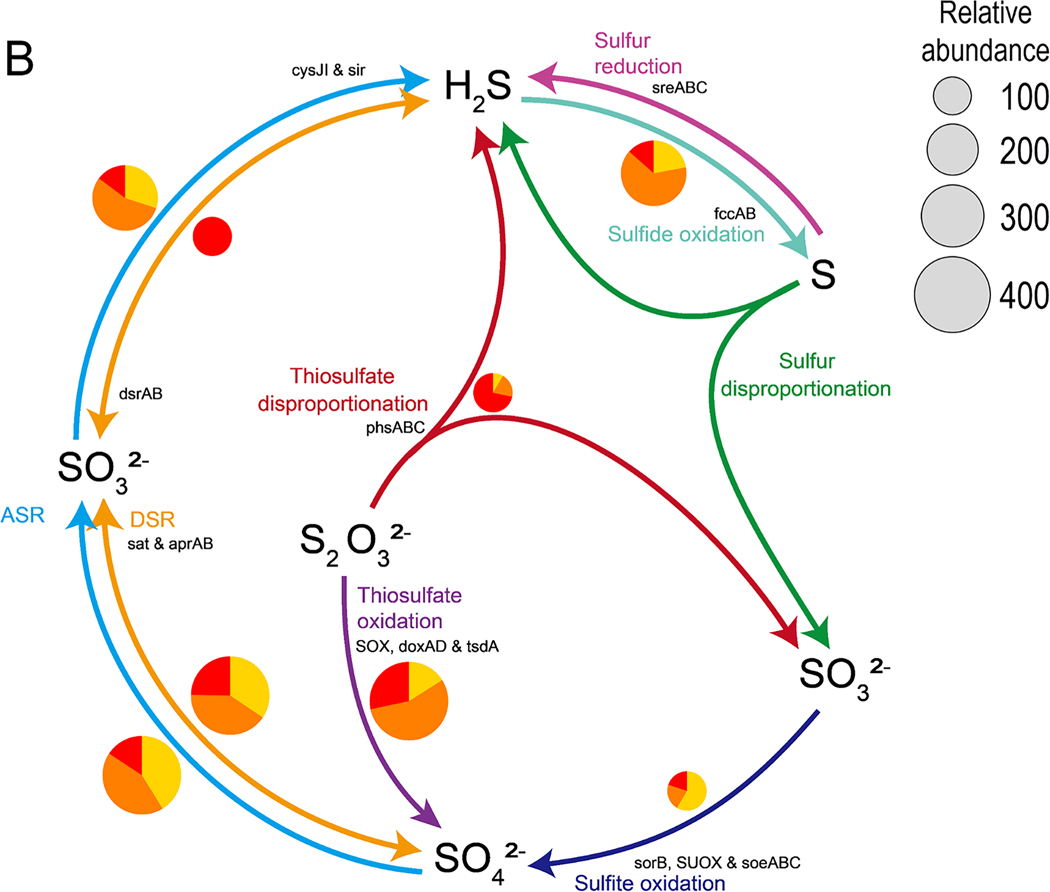
Relative abundances of the pathways involved in the (A) nitrogen and (B) sulfur cycle. The pie chart indicates the relative abundance of each pathway in each disinfectant group and the size of the pie chart is proportional to the relative abundance of the gene involved in the pathway. Disinfectant: ND, none; CHL, chlorine; CHM, chloramine. Nitrogen pathways: ANRA, assimilatory nitrate reduction to ammonium; DNRA, Dissimilatory nitrate reduction to ammonium; Anammox, anaerobic ammonium oxidation. Sulfur pathways: ASR, assimilatory sulfate reduction; DSR, dissimilatory sulfate reduction.

The complexity of biogeochemical processes (at the level of metabolic gene organization) of the water microbiome is evident in DWDSs (Figs. 3 and S11). The normalized relative abundance of genes involved in nitrogen and sulfur metabolism was higher in disinfected systems than in non-disinfected systems and is consistent with previous comparisons of drinking water samples from disinfected and non-disinfected systems (Bautista-de los Santos et al., 2016; Dai et al., 2020). This indicated that compared with the microbes in the non-disinfected systems, those in the disinfected waters tended to rely on nitrogen (denitrification, DNRA, ANRA, nitrification, comammox) and sulfur (assimilatory sulfate reduction, dissimilatory sulfate reduction to sulfite, thiosulfate oxidation, and sulfide oxidation) compounds more heavily as an energy source (Fig. 3). In DWDSs, the nitrogen and in some extent the sulfur pathway play a significant role in the ecosystem, and the populations engaged in these pathways are part of a complex and highly diverse microbial community. The transformation of nitrogen into its many redox states is key to ecosystem productivity and is driven by microbially mediated reactions. Evidence of the importance of the nitrogen biogeochemical cycle is derived from several studies of DWDSs (Dai et al., 2020; Gomez-Alvarez et al., 2012a, 2020 and 2021). Sulfur is another essential element in drinking water ecosystems that is cycled by microbes between oxidized and reduced forms (Fig. 3B). The wide range of annotated functions associated with several sulfur pathways may be indicative of the availability of several electron donors at drinking water distribution pipes undergoing corrosion (Gomez-Alvarez et al., 2012b). Concrete corrosion of distribution systems is a significant cause of deterioration and premature failure.

Nitrogen reduction pathways (denitrification, dissimilatory nitrate reduction to ammonium [DNRA], nitrogen fixation, and assimilatory nitrate reduction to ammonium [ANRA]) and oxidation pathways (nitrification, and complete ammonia oxidation [comammox]) were reconstructed from the bulk water samples (Fig. 3A). Genes related to nitrogen metabolism were identified and included those involved in (i) denitrification: cytoplasmic nitrate reductase (*narGHI*), periplasmic nitrate reductase (*napAB*), nitrite reductase (*nirKS*), nitric oxide reductase (*norBC*), and nitrous oxide reductase (*nosZ*); (ii) DNRA: cytoplasmic nitrate reductase (*narGHI*), periplasmic nitrate reductase (*napAB*), ammonia-forming dissimilatory nitrite reductase (*nrfAH*), and nitrite reductase (*nirBD*); (iii) nitrogen fixation: nitrogenase reductase (*nifDHK*); (iv) ANRA: assimilatory nitrate reductase (*nasAB*) and ferredoxin-nitrite reductase (*nirA*); (v) nitrification: ammonia monooxygenase (*amoCAB*), hydroxylamine dehydrogenase (*hao*), and nitrite oxidoreductase (*nxrAB*); and (vi) comammox: ammonia monooxygenase associated with comammox (*amoCAB*). The total abundance of N-metabolism genes was significantly higher in CHM (relative abundance [±SD] = 856 ± 108) compared with ND (296 ± 22) and CHL (376 ± 52) systems (Kruskal-Wallis: *H* = 9.7, *p* = 0.0072). The relative abundance of genes associated with denitrification (*nirK*/*nirS* and *norBC*) was higher in the microbial communities in CHM systems (mean = 333 and 17, respectively) than those in ND (68 and 8) and CHL systems (17 and 6). In CHM systems, excess free ammonia from the source water, and chloramine formation and/or decay may support an active nitrifying community (Gomez-Alvarez et al., 2021). This assumption is supported by increased levels of *amoCAB* and *hao* genes associated with nitrification in CHM systems of this study (Fig. 3A). Moreover, denitrification couples with an increased detection of *amoCAB* (ammonia monooxygenase) genes associated with comammox, suggest a strong potential of nitrogen removal in CHM systems. In contrast, the genes encoding *nirBD* and *nrfAH*, as well as *narB, nasAB*, and *nirA*, which are associated with the ANRA and DNRA pathways (respectively), were present in a higher proportion in the microbial communities from the CHL systems (Fig. 3A). Meanwhile, the proportion of genes associated with nitrogen fixation (*nifKDH*) in ND systems were significantly higher than those in CHL or in CHM systems (Kruskal-Wallis: *H* = 6.0, *p* = 0.0240).

Analysis of metagenome libraries identified key genes associated with the sulfur pathway (Fig. 3B). These functions were found to be abundant in the metagenomes, although we observed differences in the enrichment of specific gene families within the sulfur cycling. For example, the relative abundance of genes related to the processes of assimilatory sulfate reduction, dissimilatory sulfate reduction to sulfite, thiosulfate oxidation, and sulfide oxidation were significantly higher in the CHL systems (Kruskal-Wallis: *H* = 7.9, *p* = 0.0193) with sporadic difference observed in other processes (Fig. 3B). In contrast, the genes encoding *dsrAB* and *phsABC*, which are associated with dissimilatory sulfite reduction to sulfide and thiosulfate disproportionation pathways, respectively, were present in a higher proportion in the microbial communities from the ND systems than from the disinfected systems. CHM systems showed a higher representation of genes involved in sulfite oxidation, as well the genes *hydADGB* (sulfhydrogenases) and ETHE1-like sulfur dioxygenase, which are associated with sulfur- reducing and sulfur-oxidation activities, respectively (Fig. 3B).

Lastly, carbon is one of the most essential elements to living organisms and the carbon cycle illustrates the connection and exchange between heterotrophs and autotrophs in DWDS ecosystems. The presence and differences in the proportion of genes involved in carbon metabolism in the results suggest different carbon use strategies (Fig. S11). Overall, changes in water quality and the characteristics of the environment drive the variation of microbial communities which regulate core biogeochemical processes such as carbon, sulfur, and nitrogen metabolism in DWDSs. Finally, we need to understand how these biogeochemical cycles interact with one another in DWDS environments (Rousk et al., 2014).

### 3.5. Recovered metagenome assembled genomes

**Fig. 4.**
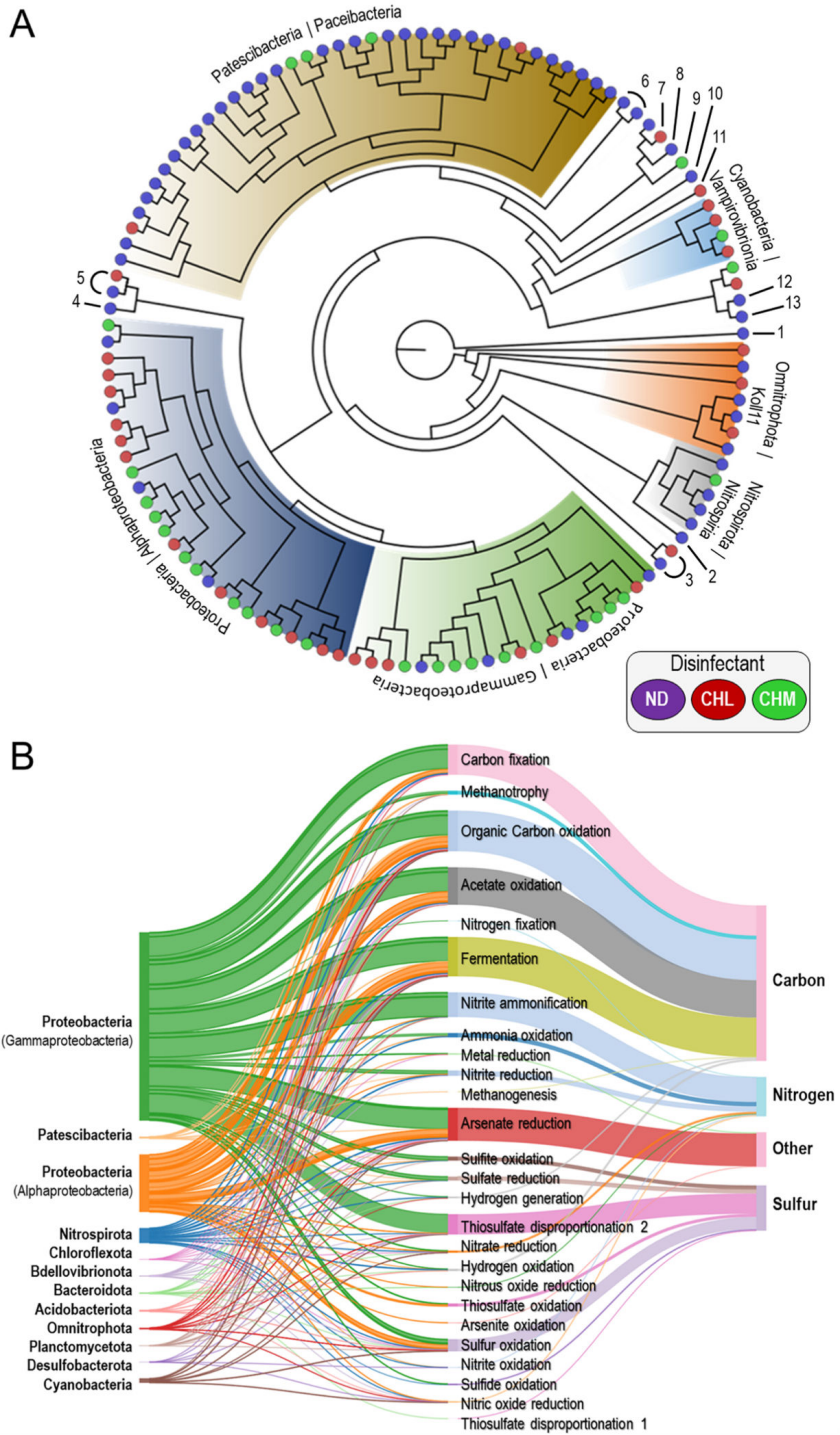
Phylogenetic assignment of MAGs and contribution to metabolic and biogeochemical processes. (A) Phylogenomic tree of de-replicated MAGs using RAxML based on the concatenated alignment of single-copy genes specific to the Archaea and Bacteria. Numbers 1 to 13 indicate the phyla (class) *Nanoarchaeota* (*Nanoarchaeia*), *Desulfobacterota* (*Binatia*), *Acidobacteriota* (*Blastoca tellia*), *Bdellovibrionota* (*Bdellovibrionia*), *Bdellovibrionota* (*Bacteriovoracia*), *Patescibacteria* (*Microgenomatia*), *Patescibacteria* (*Doudnabacteria*), *Patescibacteria* (*ABY1*), *Patescibacteria* (*Saccharimona dia*), *Patescibacteria* (*Gracilibacteria*), *Chloroflexota* (*Dehalococcoidia*), *Planctomycetota* (*Phycisphaerae*), *Planctomycetota* (*Planctomycetes*), and *Bacteroidota* (*Bacteroidia*), respectively. MAGs are colored based on disinfectant treatment. Disinfectant: ND, none; CHL, chlorine; CHM, chloramine. (B) Metabolic energy flow potential Sankey diagram at the phylum-level resolution. The three columns from left to right represent taxonomic groups scaled by the number of genomes, the contribution to each metabolic function by microbial groups calculated based on genome coverage, and the biogeochemical cycle.

Genome-centric metagenomic analysis yielded 144 genomes (143 bacterial and 1 archaeal) with 139 bins representing metagenome-assembled genomes (MAGs) that had *a* >50% completeness and <10% contamination consisting of 20 class representatives in 12 phyla (Table S3). The MAGs accounted for 43%, 83%, and 45% of reads mapping to the metagenomic data set for ND, CHL, and CHM systems, respectively (Table S4). From this data set, 88 MAGs were identified to the genus level with 7 of the MAGs having *a* >95% average nucleotide identity (ANI) to a reference genome, with the rest of the MAGs possibly representing new candidate species. MAGs analyzed with GTDB-Tk identified them as members of the phyla *Acidobacteriota* (class *Blastocatellia*), *Bacteroidota* (*Bacteroidia*), *Bdellovibrionota* (*Bacteriovoracia* and *Bdellovibrionia*), *Chloroflexota* (*Dehalococcoidia*), *Cyanobacteria* (*Vampirovibrionia*), *Desulfobacterota* (*Binatia*), *Nitrospirota* (*Nitrospiria*), *Omnitrophota* (Koll11), *Patescibacteria* (ABY1, *Doudnabacteria, Gracilibacteria, Microgenomatia, Paceibacteria*, and *Saccharimonadia*), *Planctomycetota* (*Phycisphaerae* and *Planctomycetes*), Proteobacteria (*Alphaproteobacteria* and *Gammaproteobacteria*) (Fig. S12). Only one archaea MAG was recovered and identified as member of the phylum *Nanoarchaeota* (class *Nanoarchaeia*), a phylum composed of small obligate symbionts that lack most genes involved in major biosynthetic pathways (St. John and Reysenbach, 2019). The representation of archaeal genomes in the reference database GTDB release 95 is much less complete than the representation of bacterial genomes (Parks et al., 2020). These are the first MAG representatives (including an archaeal MAG) assembled from waterworks from Finland. The MAGs data set captured the prevalent bacterial and archaeal lineages revealed by metagenome and 16S rRNA gene analysis (Figs. 1, 4, S7 and S8).

MAGs corresponding to the classes *Paceibacteria, Alphaproteobacteria, Gammaproteobacteria*, and *Nitrospiria* were differentially enriched with disinfectant treatment, consistent with their dominance at each DWDS (Fig. 4A). Among these, the MAG with the highest percentage of mapped reads belong to the class *Paceibacteria* (phylum *Patescibacteria*) and made up of approximately 26% of the ND microbial community. The analysis of 16S amplicon sequencing data supported this statement as *Paceibacteria*, belonging to different families, was among the most abundant taxa found in ND systems. This highly abundant group of common inhabitants of freshwater (Proctor et al., 2018) and groundwater environments (Brown et al., 2015) consists of small size and low-nucleic acid content bacteria of which most lack numerous biosynthetic pathways (Fig. 4B). Tian et al. (2020) proposed that the reduced functional and metabolic features combined with genomic simplicity are adaptations of *Patescibacteria* to the extreme conditions (e.g., low or lack of nutrients and oxygen) in groundwater environments.

On the other hand, MAGs corresponding to the classes *Gammaproteobacteria* (*Polaromonas* spp.) and *Alphaproteobacteria* (*Sphingorhabdus*_B spp., *Rhizobiales* spp. UBA4765, and *Hyphomicrobium* spp.) were differentially enriched in CHL systems in comparison to ND and CHM samples. These ecologically diverse microorganisms are common inhabitants in freshwater systems but are also widespread in drinking water systems (Bautista-de los Santos et al., 2016). The dominant MAG in CHL systems was the *Polaromonas* spp. comprising an average of 42% of the microbial community. *Polaromonas* is generally not observed in natural freshwater systems, but the nutritional versatility microorganism is prevalent in oligotrophic environments such as bottled mineral water (Carraturo et al., 2021). In a previous study, *Polaromonas* was identified as the predominant genus in granular activated carbon (GAC) filters from full-scale water treatment plants in the Netherlands (Magic-Knezev et al., 2009). The contribution of this taxon to the microbial community in DWDSs requires further examination. The MAGs *Sphingorhabdus*_B, *Rhizobiales* spp. UBA4765, *Hyphomicrobium* spp. represent an average of 16%, 9%, and 7% of the population in CHL systems, respectively. Members of these *Alphaproteobacteria* genera represent typical freshwater bacteria and are highly physiologically diverse (Jogler et al., 2013; Parks et al., 2017). Despite chemical disinfection, genomic annotation revealed that the group of MAGs harbors numerous biosynthetic pathways related to carbon, nitrogen, and sulfur compounds (Fig. 4B). It is evident from our analysis that the CHL community contained taxa that are metabolically diverse with lower functional redundancy compared to the ND and CHM communities, where distinct species might perform distinct metabolic roles (Fig. 2B).

The *Nitrotoga*-like MAG exhibited the highest relative abundance in the CHM system comprising 23% of the metagenomic data set followed by a *Nitrospira*-like MAG (phylum *Nitrospirota*) comprising 7%. Both MAGs were identified as nitrite-oxidizing bacteria (NOB) which play a critical role in the biogeochemical nitrogen cycle by metabolizing nitrite to nitrate (Fig. 4B). NOB are physiologically versatile, widely distributed, and may have diverse ecological functions within and beyond the nitrogen cycle, including carbon, hydrogen, and sulfur biogeochemical metabolism (Daims et al., 2016). The known phylogenetic diversity of NOB (classes *Alphaproteobacteria* and *Gammaproteobacteria* and the phyla *Nitrospirota* and *Nitrospinota*) has been now expanded by the description of several new NOB lineages including the genus *Nitrotoga* (Alawi et al., 2007). Boddicker and Mosier (2018) estimated the relative abundance of *Nitrotoga*-like sequences to be as high as 10% of the total microbial community across globally distributed freshwater habitats. *Nitrotoga* may play a critical role in the biogeochemical nitrogen cycle (i. e., nitrite oxidation) in engineered environments (Alawi et al., 2009; Lücker et al., 2015). Moreover, the genomic characterization of *Nitrotoga* has revealed potential alternative energy metabolisms and a broader spectrum of physiological adaptations which may explain their competitive adaptation in engineered environments (Kitzinger et al., 2018). Further research is needed to understand which environmental conditions allow the coexistence of *Nitrotoga* with *Nitrospira* (or other NOB) and which factors lead to their dominance in CHM DWDSs. Overall, the presence and occurrence of NOB-like microorganisms have significant implications for nitrogen biotransformation in drinking water systems (Gomez-Alvarez et al., 2015, 2016 and 2020), particularly in storage tanks (Gomez-Alvarez et al., 2021).

### 3.6. Active populations in non-disinfected and disinfected systems

We compared the relative abundance of metagenomic (DNA) and metatranscriptomic (cDNA) reads to determine the extent to which the total taxonomic and functional potential abundance of the microbial community correlated with its active population. Herein, for the purpose of this discussion, the relative abundance obtained from the metagenome and metatranscriptome libraries will be referred to as the total and active fraction of the community, respectively. Only the data sets of sites A (ND) and D (CHM) sets were used to determine which populations were metabolically active at the time of sampling. While the sequencing depth in this study compensated in part for the limited number of samples that were analyzed (*n* = 8), additional 16S rRNA and metagenomic surveys are needed to better understand the total microbial genetic potential of these systems. However, through randomization procedures (i.e., Fisher’s exact test with Storey’s FDR multiple test correction approach) we found statistically distinct taxonomic and functional groups in each of the DWDS samples. Similar taxonomic groups (i.e., membership) were identified in the total and active community at the phylum and class level for the dominant taxa but of these dominant taxa showed different structure patterns (i.e., distribution) in their active community (Fisher’s exact test, *q* < 0.0001). For example, in ND systems the class *Paceibacteria* dominated the total community (36%), but a shift was observed to the classes *Alphaproteobacteria* (from 15% to 21%), *Gammaproteobacteria* (13% to 28%), and *Nitrospiria* (1% to 9%) in the active community (Fig. S14A). *Paceibacteria* was reduced to only 5% of the active ND community. It is important to emphasize that members of the class *Paceibacteria* consists of small size cells with low-nucleic acid content that lack numerous biosynthetic pathways (Tian et al., 2020). To avoid predation and endure at these environments these microorganisms with highly reduced genomes may adjust their metabolism and rely on simple intermediate metabolites from a host-associated lifestyle for energy. Similarly, a moderate shift in the structure of the CHM community was observed with an increase of the classes *Gammaproteobacteria* (from 45% to 56%) and *Nitrospiria* (14% to 25%) while the rest of the classes decreased in the active community (Fisher’s exact test, *q* < 0.0001) (Fig. S16A).

**Fig. 5.**
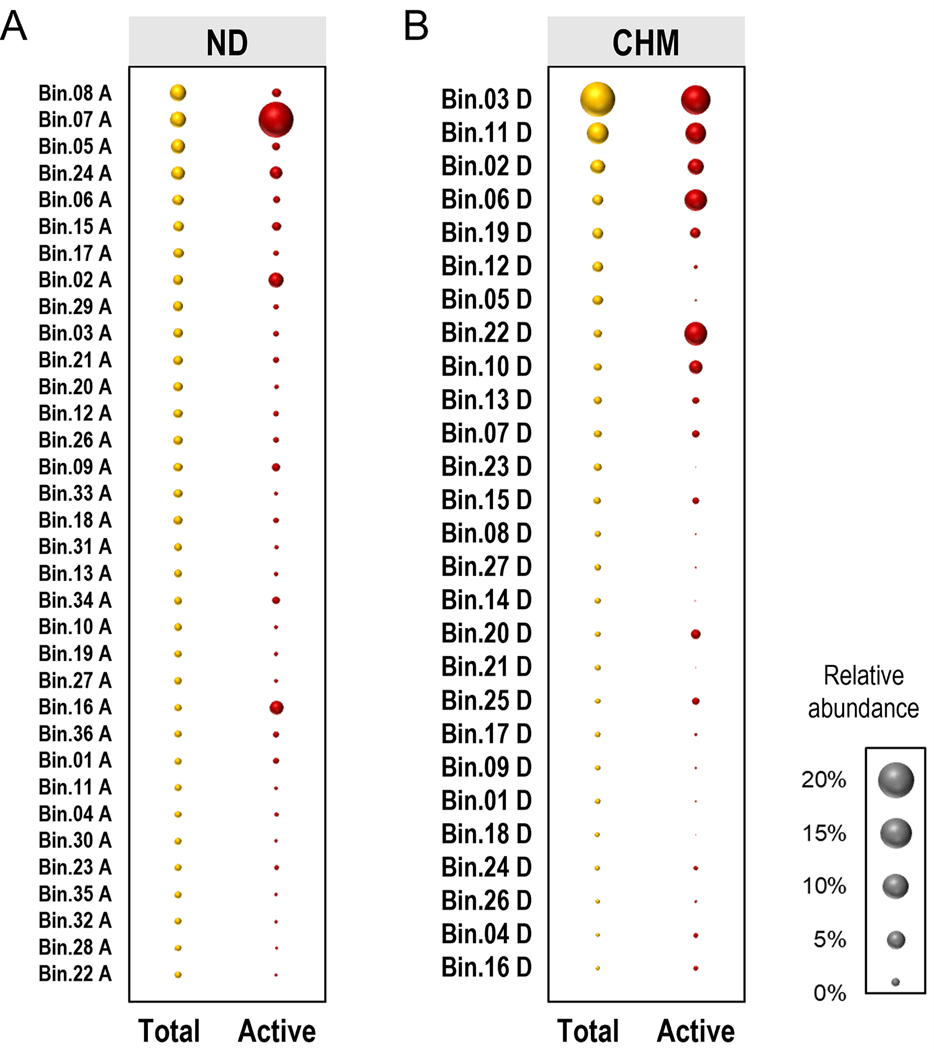
Relative abundance of MAGs recovered from ND and CHM contributing to the total and active fraction of the community. MAGs are ordered from top to bottom by the most abundant MAGs in the total community. Bubble size indicates the relative abundance of the MAG in the total community in orange (●) and the active population in red (●). The relative abundance (%) in the total community was estimated as a proportion of a bin relative to the number of reads mapped to assembled contigs from metagenome and adjusted for the size of the bin, while the active fraction of the community was calculated as a proportion of metatranscriptome reads mapping to a MAG. Taxonomic classification and coverage for each MAG is listed in Table S4.

To determine whether there were any patterns in microbial activity distinguishing ND and CHM systems at various taxonomic levels, we generated a flow diagram based on their respective patterns of relative abundance. The analysis of ND systems indicated that the top ten group-level taxa (at various taxonomic levels) decreased in the active community (Fig. S14B). A similar pattern occurred for the MAGs recovered from ND systems, where 91% (31 out of 34) experienced an average of 0.4-fold change decrease (Fig. 5A). *Paceibacteria* MAGs, which were the most abundant in the total community were among those with lower significance abundance in the active fraction of the community (Fisher’s exact test, *q* = 0.0236), while *Gammaproteobacteria*-assigned MAGs expressed higher relative abundance in the active community (Fisher’s exact test, *q* = 0.0007). For example, the MAG members Bin.07 A (*Polynucleobacter* spp.) and Bin.02 A (*Nitrospira* spp.) increased from a relative abundance of 3.4% to 18.5% and 1.5% to 3.2%, respectively. *Polynucleobacter* is a bacterioplankton and member of the family *Burkholderiaceae* widely detected in freshwater environments (Watanabe et al., 2009). It is unclear why these taxonomic group showed higher abundances in ND systems (Waak et al., 2019). It can be speculated that recharge water (i.e., artificial groundwater) brings along nutrients that specifically favors these microorganisms (Abiriga et al., 2021).

Contrary to the ND systems, the analysis of the CHM system indicated an increase in the active community for most of the dominant group-level taxa at various taxonomic levels (Fig. S16B). This was confirmed by the increase of the top dominant MAGs, where 33% (9 out of 27) experienced an average of 2.7-fold change increase in the active community (Fig. 5B). Compared to the CHM system, only 3 MAGs (9% of the total set) from ND systems showed an increased in relative abundance in the active community with an average of 3.9-fold increase (up to 290% change), while only 67% (18 out of 27) in the CHM system experienced an average of 0.3-fold change decrease (Fig. 5B). These MAGs conserved similar levels of distribution in the total and active communities (Fisher’s exact test, *q* = 1.0). The MAGs *Nitrotoga* (Bin.03 D), *Burkholderiaceae* PHCI01 (Bin.22 D), *Sphingomonas* (Bin.06 D), and *Nitrospira* (Bin.11 D) maintained their dominance in the active CHM community. *Nitrotoga* and *Nitrospira* are the main NOB populations in aquatic environments (Spieck et al., 2021). In chloraminated-treated drinking water distribution systems, *Nitrotoga* coexists together with the *Nitrospira* populations (Waak et al., 2019) which also co-occurs with heterotrophic bacteria classified as *Rhizobiales* and *Sphingomonas* (Potgieter et al., 2020). The current study suggested the relevance of co-occurrence relationships which may be important for understanding community assembly and ecosystem functions in DWDS.

In addition to community-level taxonomic analysis, we also examined and compared the relative abundance of metabolic processes in the active fraction of the community. In ND systems metabolic genes were universally present but only a significant increase in genes related to nitrification and methane oxidation were detected at higher relative abundances in the active ND community (Fisher’s exact test, *q* < 0.0001) (Fig. S13). For example, an increase of ammonia monooxygenase (*amoCAB*) and hydroxylamine dehydrogenase (*hao*) genes in the nitrification pathway was detected (Fig. S14C). This is consistent with changes in the taxonomic composition in the active ND population where the NOB group *Nitrospiria* increased in relative abundance from 1.2% to 9.1% (Fisher’s exact test, *q* < 0.0001) (Figs. S14A and B). Contrary to the ND systems, the analysis of the CHM system indicated a noticeably difference in operational metabolic pathways between the total and active community (Fig. S15). In CHM systems genes related to nitrification and DNRA were detected at higher relative abundances in the active CHM community (Fisher’s exact test, *q* < 0.0001) (Fig. S16C). This corresponds to an increase of the genera *Nitrosomonas* (Fig. S16B), a chemoautotrophic bacterium responsible for the biological oxidation of ammonia/ ammonium to nitrite. To determine whether there were any patterns in microbial activity distinguishing ND and CHM systems at the population level, we compared the coverage of the metabolic and biogeochemical functional traits of the recovered MAGs with their respective metagenomic and metatranscriptomic data sets. MAG coverages in ND and CHM systems accounted for only 19% and 43% of the metabolic pathways in total and active communities (Fig. S17). In general, recovered MAGs from the CHM system may represent a considerable proportion of the active bacterial population, where MAGs from the ND systems represent only a reduced portion of the active community. Given these results, the broader set of genes transcribed in both drinking water ecosystems (i.e., DWDS) may indicate an active and diverse community regardless of the treatment methods applied to the water (Table S1).

## 4. Conclusions

- The microbial communities in the DWDS sites exhibit a distribution pattern of a few dominant taxa and a large representation of low-abundance bacterial species.
- Metagenomes and 16S rRNA-based analysis of the Bacteria and Archaea domain confirmed that DWDSs with non-disinfected water harbor higher microbial richness and composition. This suggest that maintaining disinfectant residual is significantly important for ensuring low microbial numbers and diversity.
- The observed changes in the structure of the microbial community correspond to the presence or absence and type of disinfectant residual suggesting that these conditions exert selective pressure on the microbial community.
- The archaea domain in DWDSs represents a small fraction of the prokaryote community and seemed to be effectively controlled by disinfection of water. One archaea MAG was recovered and was identified as *Nanoarchaeota*, a phylum composed of small obligate symbionts that lack most genes involved in major biosynthetic pathways. Their role particularly in non-disinfected DWDS may be more important than previously considered.
- Selection pressures exerted within disinfected systems are not only evident at the community structure level, but also evident at the functional and metabolic potential level.
- The presence of a diverse group of opportunistic pathogens combined with the occurrence of microbial resistance mechanisms may constitute a significant challenge for drinking water treatment efficiency and affect drinking water safety.
- Comparative analysis of the community suggested the relevance of co-occurrence relationships in the biological stability of drinking water systems. The presence and occurrence of NOB-like microorganisms have significant implications for nitrogen biotransformation in drinking water systems.
- The broader set of genes annotated and transcribed in non-disinfected and disinfected systems may indicate an active and diverse community regardless of the treatment methods applied to the water.

## Supplementary Material

Supplementary Material

## Data Availability

Data will be made available on request.

## References

[R1] Abiriga D, Jenkins A, Alfsnes K, Vestgarden LS, Klempe H, 2021. Spatiotemporal and seasonal dynamics in the microbial communities of a landfill-leachate contaminated aquifer. FEMS Microbiol. Ecol. 97, fiab086. 10.1093/femsec/fiab086. PMC8247425 34137824

[R2] Alawi M, Lipski A, Sanders T, Pfeiffer EM, Spieck E, 2007. Cultivation of a novel cold-adapted nitrite oxidizing betaproteobacterium from the Siberian Arctic. ISME J. 1, 256–264. 10.1038/ismej.2007.34. 18062041

[R3] Alawi M, Off S, Kaya M, Spieck E, 2009. Temperature influences the population structure of nitrite-oxidizing bacteria in activated sludge. Environ. Microbiol. Rep. 1, 184–190. 10.1111/j.1758-2229.2009.00029.x. 23765792

[R4] Anderson MJ, 2001. A new method for non-parametric multivariate analysis of variance. Austral. Ecol. 26, 32–46. 10.1111/j.1442-9993.2001.01070.pp.x.

[R5] Ashbolt NJ, 2015. Microbial contamination of drinking water and human health from community water systems. Curr. Environ. Health Rep. 2, 95–106. 10.1007/s40572-014-0037-5. 25821716 PMC4372141

[R6] Asnicar F, Thomas AM, Beghini F, Mengoni C, Manara S, Manghi P, Zhu Q, Bolzan M, Cumbo F, May U, Sanders JG, Zolfo M, Kopylova E, Pasolli E, Knight R, Mirarab S, Huttenhower C, Segata N, 2020. Precise phylogenetic analysis of microbial isolates and genomes from metagenomes using PhyloPhlAn 3.0. Nat. Commun. 11, 2500. 10.1038/s41467-020-16366-7. 32427907 PMC7237447

[R7] Bautista-de los Santos QM, Schroeder JL, Sevillano-Rivera MC, Sungthong R, Ijaz UZ, Sloan WT, Pinto AJ, 2016. Emerging investigators series: microbial communities in full-scale drinking water distribution systems - a meta-analysis. Environ. Sci. Water Res. Technol. 2, 631–644. 10.1039/C6EW00030D.

[R8] Beghini F, McIver LJ, Blanco-Míguez A, Dubois L, Asnicar F, Maharjan S, Mailyan A, Manghi P, Scholz M, Thomas AM, Valles-Colomer M, Weingart G, Zhang Y, Zolfo M, Huttenhower C, Franzosa EA, Segata N, 2021. Integrating taxonomic, functional, and strain-level profiling of diverse microbial communities with bioBakery 3. Elife 10, e65088. 10.7554/eLife.65088. PMC8096432 33944776

[R9] Boddicker AM, Mosier AC, 2018. Genomic profiling of four cultivated *Candidatus* Nitrotoga spp. predicts broad metabolic potential and environmental distribution. ISME J. 12, 2864–2882. 10.1038/s41396-018-0240-8. 30050164 PMC6246548

[R10] The Genome Standards Consortium Bowers RM, Kyrpides NC, Stepanauskas R, Harmon-Smith M, Doud D, Reddy TBK, Schulz F, Jarett J, Rivers AR, Eloe-Fadrosh EA, Tringe SG, Ivanova NN, Copeland A, Clum A, Becraft ED, Malmstrom RR, Birren B, Podar M, Bork P, Weinstock GM, Garrity GM, Dodsworth JA, Yooseph S, Sutton G, Glockner FO, Gilbert JA, Nelson WC, ¨ Hallam SJ, Jungbluth SP, Ettema TJG, Tighe S, Konstantinidis KT, Liu W−T, Baker BJ, Rattei T, Eisen JA, Hedlund B, McMahon KD, Fierer N, Knight R, Finn R, Cochrane G, Karsch-Mizrachi I, Tyson GW, Rinke C, Lapidus A, Meyer F, Yilmaz P, Parks DH, Murat Eren A, Schriml L, Banfield JF, Hugenholtz P, Woyke T, 2017. Minimum information about a single amplified genome (MISAG) and a metagenome-assembled genome (MIMAG) of Bacteria and Archaea. Nat. Biotechnol. 35, 725–731. 10.1038/nbt.3893. 28787424 PMC6436528

[R11] Breitwieser FP, Salzberg SL, 2020. Pavian: interactive analysis of metagenomics data for microbiome studies and pathogen identification. Bioinformatics 36, 1303–1304. 10.1093/bioinformatics/btz715. 31553437 PMC8215911

[R12] Brown CT, Hug LA, Thomas BC, Sharon I, Castelle CJ, Singh A, Wilkins MJ, Wrighton KC, Williams KH, Banfield JF, 2015. Unusual biology across a group comprising more than 15% of domain Bacteria. Nature 523, 208–211. 10.1038/nature14486. 26083755

[R13] Brumfield KD, Hasan NA, Leddy MB, Cotruvo JA, Rashed SM, Colwell RR, Huq A, 2020. A comparative analysis of drinking water employing metagenomics. PLoS One 15, e0231210. 10.1371/journal.pone.0231210. PMC7145143 32271799

[R14] Carraturo F, Del Giudice C, Compagnone M, Libralato G, Toscanesi M, Trifuoggi M, Galdiero E, Guida M, 2021. Evaluation of microbial communities of bottled mineral waters and preliminary traceability analysis using NGS microbial fingerprints. Water 13, 2824. 10.3390/w13202824 (Basel).

[R15] Chaumeil PA, Mussig AJ, Hugenholtz P, Parks DH, 2019. GTDB-Tk: a toolkit to classify genomes with the genome taxonomy database. Bioinformatics 36, 1925–1927. 10.1093/bioinformatics/btz848. 31730192 PMC7703759

[R16] Clarke KR, 1993. Non-parametric multivariate analyses of changes in community structure. Aust. J. Ecol. 18, 117–143. 10.1111/j.1442-9993.1993.tb00438.x.

[R17] Dai Z, Sevillano-Rivera MC, Calus ST, Bautista-de los Santos QM, Eren AM, van der Wielen PWJJ, Ijaz UZ, Pinto AJ, 2020. Disinfection exhibits systematic impacts on the drinking water microbiome. Microbiome 8, 42. 10.1186/s40168-020-00813-0. 32197656 PMC7085177

[R18] Daims H, Lücker S, Wagner MA, 2016. New perspective on microbes formerly known as nitrite-oxidizing bacteria. Trends Microbiol. 24, 699–712. 10.1016/j.tim.2016.05.004. 27283264 PMC6884419

[R19] Darzi Y, Yamate Y, Yamada T, 2019. FuncTree2: an interactive radial tree for functional hierarchies and omics data visualization. Bioinformatics 35, 4519–4521. 10.1093/bioinformatics/btz245. 31004476 PMC6821421

[R20] Douterelo I, Calero-Preciado C, Soria-Carrasco V, Boxall JB, 2018. Whole metagenome sequencing of chlorinated drinking water distribution systems. Environ. Sci. Water Res. Technol. 4, 2080–2091. 10.1039/C8EW00395E.

[R21] Eren AM, Esen ÖC, Quince C, Vineis JH, Morrison HG, Sogin ML, Delmont TO, 2015. Anvi’o: an advanced analysis and visualization platform for ‘omics data. PeerJ 3, e1319. 10.7717/peerj.1319. PMC4614810 26500826

[R22] Gantner S, Andersson AF, Alonso-Sáez L, Bertilsson S, 2011. Novel primers for 16S rRNA-based archaeal community analyses in environmental samples. J. Microbiol. Methods 84, 12–18. 10.1016/j.mimet.2010.10.001. 20940022

[R23] Gao R, Sui M, 2021. Antibiotic resistance fate in the full-scale drinking water and municipal wastewater treatment processes: a review. Environ. Eng. Res. 26, 200324 10.4491/eer.2020.324.

[R24] Garner E, Davis BC, Milligan E, Blair MF, Keenum I, Maile-Moskowitz A, Pan J, Gnegy M, Liguori K, Gupta S, Prussin AJ 2nd, Marr LC, Heath LS, Vikesland PJ, Zhang L, Pruden A, 2021. Next generation sequencing approaches to evaluate water and wastewater quality. Water Res. 194, 116907 10.1016/j.watres.2021.116907. 33610927

[R25] Ghosh S. 2021. Pathogen-fluctuations (Mar 2, 2021). Available at: https://github.com/sudeshna-ghosh/Pathogen-fluctuations (accessed 1 June 2021).

[R26] Gomez-Alvarez V, Revetta RP, Santo Domingo JW, 2012a. Metagenomic analyses of drinking water receiving different disinfection treatments. Appl. Environ. Microbiol. 78, 6095–6102. 10.1128/AEM.01018-12. 22729545 PMC3416622

[R27] Gomez-Alvarez V, Revetta RP, Santo Domingo JW, 2012b. Metagenome analyses of corroded concrete wastewater pipe biofilms reveal a complex microbial system. BMC Microbiol. 12, 122. 10.1186/1471-2180-12-122. 22727216 PMC3409016

[R28] Gomez-Alvarez V, Humrighouse BW, Revetta RP, Santo Domingo JW, 2015. Bacterial composition in a metropolitan drinking water distribution system utilizing different source waters. J. Water Health 13, 140–151. 10.2166/wh.2014.057. 25719474

[R29] Gomez-Alvarez V, Pfaller S, Pressman JG, Wahman DG, Revetta RP, 2016. Resilience of microbial communities in a simulated drinking water distribution system subjected to disturbances: role of conditionally rare taxa and potential implications for antibiotic-resistant bacteria. Environ. Sci. Water Res. Technol. 2, 645–657. 10.1039/c6ew00053c.

[R30] Gomez-Alvarez V, Revetta RP, 2020. Monitoring of nitrification in chloraminated drinking water distribution systems with microbiome bioindicators using supervised machine learning. Front. Microbiol. 11, 571009 10.3389/fmicb.2020.571009. PMC7526508 33042076

[R31] Gomez-Alvarez V, Liu H, Pressman JG, Wahman DG, 2021. Metagenomic profile of microbial communities in a drinking water storage tank sediment after sequential exposure to monochloramine, free chlorine, and monochloramine. ACS ES&T Water 1, 1283–1294. 10.1021/acsestwater.1c00016. 34337601 PMC8318090

[R32] Hammer Ø, Harper DAT, Ryan PD, 2001. PAST: paleontological statistics software package for education and data analysis. Palaeontol. Electron. 4, 1–9 palaeo-electronica.org/2001_1/past/issue1_01.htm.

[R33] Hydes O, 1999. European regulations on residual disinfectant. J. Am. Water Works Assoc. 91, 70–74. 10.1002/j.1551-8833.1999.tb08570.x.

[R34] Ikonen JM, Hokajärvi A−M, Heikkinen J, Pitkänen T, Ciszek R, Kolehmainen M, 2017. Drinking water quality in distribution systems of surface and ground waterworks in Finland. J. Water Secur 3, 1–10. 10.15544/jws.2017.004.

[R35] Inkinen J, Jayaprakash B, Siponen S, Hokajärvi A, Pursiainen A, Ikonen J, 2019. Active eukaryotes in drinking water distribution systems of ground and surface waterworks. Microbiome 7, 1–17. 10.1186/s40168-019-0715-5. 31269979 PMC6610866

[R36] Inkinen J, Siponen S, Jayaprakash B, Tiwari A, Hokajärvi AM, Pursiainen A, Ikonen J, Kauppinen A, Miettinen IT, Paananen J, Torvinen E, Kolehmainen M, Pitkänen T, 2021. Diverse and active archaea communities occur in non-disinfected drinking water systems-Less activity revealed in disinfected and hot water systems. Water Res. X 12, 100101. 10.1016/j.wroa.2021.100101. PMC8131914 34027378

[R37] Jogler M, Chen H, Simon J, Rohde M, Busse HJ, Klenk HP, Tindall BJ, Overmann J, 2013. Description of *Sphingorhabdus planktonica* gen. nov., sp. nov. and reclassification of three related members of the genus *Sphingopyxis* in the genus *Sphingorhabdus* gen. nov. Int. J. Syst. Evol. Microbiol. 63, 1342–1349. 10.1099/ijs.0.043133-0. 22798658

[R38] Kang DD, Li F, Kirton E, Thomas A, Egan R, An H, Wang Z, 2019. MetaBAT 2: an adaptive binning algorithm for robust and efficient genome reconstruction from metagenome assemblies. PeerJ 7, e7359. 10.7717/peerj.7359. PMC6662567 31388474

[R39] Kitzinger K, Koch H, Lücker S, Sedlacek CJ, Herbold C, Schwarz J, Daebeler A, Mueller AJ, Lukumbuzya M, Romano S, Leisch N, Karst SM, Kirkegaard R, Albertsen M, Nielsen PH, Wagner M, Daims H, 2018. Characterization of the first “*Candidatus* Nitrotoga” isolate reveals metabolic versatility and separate evolution of widespread nitrite-oxidizing bacteria. MBio 9. 10.1128/mBio.01186-18e01186-18. PMC6050957 29991589

[R40] Klindworth A, Pruesse E, Schweer T, Peplies J, Quast C, Horn M, Glöckner FO, 2013. Evaluation of general 16S ribosomal RNA gene PCR primers for classical and next-generation sequencing-based diversity studies. Nucleic Acids. Res. 41, e1. 10.1093/nar/gks808. 22933715 PMC3592464

[R41] Kopylova E, Noé L, Touzet H, 2012. SortMeRNA: fast and accurate filtering of ribosomal RNAs in metatranscriptomic data. Bioinformatics 28, 3211–3217. 10.1093/bioinformatics/bts611. 23071270

[R42] Kumar S, Stecher G, Li M, Knyaz C, Tamura K, 2018. MEGA X: molecular evolutionary genetics analysis across computing platforms. Mol. Biol. Evol. 35, 1547–1549. 10.1093/molbev/msy096. 29722887 PMC5967553

[R43] Li D, Luo R, Liu CM, Leung CM, Ting HF, Sadakane K, Yamashita H, Lam TW, 2016. MEGAHIT v1.0: a fast and scalable metagenome assembler driven by advanced methodologies and community practices. Methods 102, 3–11. 10.1016/j.ymeth.2016.02.020. 27012178

[R44] Lu J, Breitwieser FP, Thielen P, Salzberg SL, 2017. Bracken: estimating species abundance in metagenomics data. PeerJ Comput. Sci. 3, e104. 10.7717/peerj-cs.104. PMC12016282 40271438

[R45] Lücker S, Schwarz J, Gruber-Dorninger C, Spieck E, Wagner M, Daims H, 2015. *Nitrotoga*-like bacteria are previously unrecognized key nitrite oxidizers in full-scale wastewater treatment plants. ISME J. 9, 708–720. 10.1038/ismej.2014.158. 25180967 PMC4270731

[R46] Magic-Knezev A, Wullings B, Van der Kooij D, 2009. *Polaromonas* and *Hydrogenophaga* species are the predominant bacteria cultured from granular activated carbon filters in water treatment. J. Appl. Microbiol. 107, 1457–1467. 10.1111/j.1365-2672.2009.04337.x. 19457026

[R47] Nayfach S, Shi ZJ, Seshadri R, Pollard KS, Kyrpides NC, 2019. New insights from uncultivated genomes of the global human gut microbiome. Nature 568, 505–510. 10.1038/s41586-019-1058-x. 30867587 PMC6784871

[R48] Nemergut DR, Schmidt SK, Fukami T, O’Neill SP, Bilinski TM, Stanish LF, Knelman JE, Darcy JL, Lynch RC, Wickey P, Ferrenberg S, 2013. Patterns and processes of microbial community assembly. Microbiol. Mol. Biol. Rev. 77, 342–356. 10.1128/MMBR.00051-12. 24006468 PMC3811611

[R49] Olm MR, Brown CT, Brooks B, Banfield JF, 2017. dRep: a tool for fast and accurate genomic comparisons that enables improved genome recovery from metagenomes through de-replication. ISME J. 11, 2864–2868. 10.1038/ismej.2017.126. 28742071 PMC5702732

[R50] Parks DH, Beiko RG, 2010. Identifying biologically relevant differences between metagenomic communities. Bioinformatics 26, 715–721. 10.1093/bioinformatics/btq041. 20130030

[R51] Parks DH, Imelfort M, Skennerton CT, Hugenholtz P, Tyson GW, 2015. CheckM: assessing the quality of microbial genomes recovered from isolates, single cells, and metagenomes. Genome Res. 25, 1043–1055. 10.1101/gr.186072.114. 25977477 PMC4484387

[R52] Parks DH, Rinke C, Chuvochina M, Chaumeil PA, Woodcroft BJ, Evans PN, Hugenholtz P, Tyson GW, 2017. Recovery of nearly 8,000 metagenome-assembled genomes substantially expands the tree of life. Nat. Microbiol. 2, 1533–1542. 10.1038/s41564-017-0012-7. 28894102

[R53] Parks DH, Chuvochina M, Chaumeil PA, Rinke C, Mussig AJ, Hugenholtz P, 2020. A complete domain-to-species taxonomy for Bacteria and Archaea. Nat. Biotechnol. 38, 1079–1086. 10.1038/s41587-020-0501-8. 32341564

[R54] Potgieter SC, Dai Z, Venter SN, Sigudu M, Pinto AJ, 2020. Microbial nitrogen metabolism in chloraminated drinking water reservoirs. mSphere 5, e00274. 10.1128/mSphere.00274-20.-20. PMC7193043 32350093

[R55] Proctor CR, Besmer MD, Langenegger T, Beck K, Walser JC, Ackermann M, Bürgmann H, Hammes F, 2018. Phylogenetic clustering of small low nucleic acid-content bacteria across diverse freshwater ecosystems. ISME J. 12, 1344–1359. 10.1038/s41396-018-0070-8. 29416124 PMC5932017

[R56] Prüss-Üstün A, Bos R, Gore F, Bartram J, 2008. Safer Water, Better Health: Costs, Benefits and Sustainability of Interventions to Protect and Promote Health. World Health Organization, Geneva. https://apps.who.int/iris/handle/10665/43840.

[R57] Rambaut A. 2018. FigTree v1.4.4. Available at: https://github.com/rambaut/figtree (accessed 18 February 2021).

[R58] Rousk J, Bengtson P, 2014. Microbial regulation of global biogeochemical cycles. Front. Microbiol. 5, 103. 10.3389/fmicb.2014.00103. 24672519 PMC3954078

[R59] Sanganyado E, Gwenzi W, 2019. Antibiotic resistance in drinking water systems: occurrence, removal, and human health risks. Sci. Total Environ. 669, 785–797. 10.1016/j.scitotenv.2019.03.162. 30897437

[R60] Schloss PD, Westcott SL, Ryabin T, Hall JR, Hartmann M, Hollister EB, Lesniewski RA, Oakley BB, Parks DH, Robinson CJ, Sahl JW, Stres B, Thallinger GG, Van Horn DJ, Weber CF, 2009. Introducing mothur: open-source, platform-independent, community-supported software for describing and comparing microbial communities. Appl. Environ. Microbiol. 75, 7537–7541. 10.1128/AEM.01541-09. 19801464 PMC2786419

[R61] Seemann T, 2014. Prokka: rapid prokaryotic genome annotation. Bioinformatics 30, 2068–2069. 10.1093/bioinformatics/btu153. 24642063

[R62] Sieber CMK, Probst AJ, Sharrar A, Thomas BC, Hess M, Tringe SG, Banfield JF, 2018. Recovery of genomes from metagenomes via a dereplication, aggregation and scoring strategy. Nat. Microbiol. 3, 836–843. 10.1038/s41564-018-0171-1. 29807988 PMC6786971

[R63] Spieck E, Wegen S, Keuter S, 2021. Relevance of *Candidatus* Nitrotoga for nitrite oxidation in technical nitrogen removal systems. Appl. Microbiol. Biotechnol. 105, 7123–7139. 10.1007/s00253-021-11487-5. 34508283 PMC8494671

[R64] St. John E, Reysenbach A−L, 2019. Nanoarchaeota. Schmidt TM Encyclopedia of Microbiology Fourth Edition. Academic Press, Massachusetts, USA, pp. 274–279. 10.1016/B978-0-12-809633-8.20766-8.

[R65] Stahl DA, Amann R, 1991. Development and application of nucleic acid probes in bacterial systematics. Stackebrandt E, Goodfellow M. Nucleic Acid Techniques in Bacterial Systematics. John Wiley and Sons Ltd., Chichester, UK, pp. 205–248. 10.1002/jobm.3620310616.

[R66] Stamatakis A, 2014. RAxML version 8: a tool for phylogenetic analysis and post-analysis of large phylogenies. Bioinformatics 30, 1312–1313. 10.1093/bioinformatics/btu033. 24451623 PMC3998144

[R67] Suzek BE, Wang Y, Huang H, McGarvey PB, Wu CH, 2015. UniProt consortium. UniRef clusters: a comprehensive and scalable alternative for improving sequence similarity searches. Bioinformatics 31, 926–932. 10.1093/bioinformatics/btu739. 25398609 PMC4375400

[R68] Tan B, Ng C, Nshimyimana JP, Loh LL, Gin KY, Thompson JR, 2015. Next-generation sequencing (NGS) for assessment of microbial water quality: current progress, challenges, and future opportunities. Front. Microbiol. 6, 1027. 10.3389/fmicb.2015.01027. 26441948 PMC4585245

[R69] Telatin A. 2020. MetaProkka v1.14.6_1. Available at: https://github.com/telatin/metaprokka (accessed 1 March 2021).

[R70] Tian R, Ning D, He Z, Zhang P, Spencer SJ, Gao S, Shi W, Wu L, Zhang Y, Yang Y, Adams BG, Rocha AM, Detienne BL, Lowe KA, Joyner DC, Klingeman DM, Arkin AP, Fields MW, Hazen TC, Stahl DA, Alm EJ, Zhou J. 2020. Small and mighty: adaptation of superphylum *Patescibacteria* to groundwater environment drives their genome simplicity. Microbiome 8, 51. doi: 10.1186/s40168-020-00825-w. 32252814 PMC7137472

[R71] Tiwari A, Gomez-Alvarez V, Siponen S, Sarekoski A, Hokajärvi A−M, Kauppinen A, Torvinen, Miettinen IT, Pitkanen T, 2022. Bacterial genes encoding resistance against antibiotics and metals in well-maintained drinking water distribution systems in Finland. Front. Microbiol. 12, 803094 10.3389/fmicb.2021.803094. PMC8859300 35197945

[R72] Uritskiy GV, DiRuggiero J, Taylor J. 2018. MetaWRAP-a flexible pipeline for genome-resolved metagenomic data analysis. Microbiome 6, 158. doi: 10.1186/s40168-018-0541-1. 30219103 PMC6138922

[R73] USEPA, 2007. The effectiveness of disinfectant residuals in the distribution system. Office of Water, Office of Ground Water and Drinking Water. In: Proceedings of the Total Coliform Rule Issue Paper. U.S. Environmental Protection Agency, Washington, DC. https://www.epa.gov/sites/default/files/2021-05/documents/effectiveness_of_disinfectant_residuals_final_-_3-7-07.pdf. Accessed on August 5, 2022.

[R74] Yates MV, 2019. Drinking water microbiology. Schmidt TM Encyclopedia of Microbiology, 4th ed. Academic Press, USA, pp. 83–89. 10.1016/B978-0-12-801238-3.66123-8.

[R75] Waak MB, Hozalski RM, Hallé C, LaPara TM, 2019. Comparison of the microbiomes of two drinking water distribution systems-with and without residual chloramine disinfection. Microbiome 7, 87. 10.1186/s40168-019-0707-5. 31174608 PMC6556008

[R76] Wang Y, Zhang R, He Z, Van Nostrand JD, Zheng Q, Zhou J, Jiao N, 2017. Functional gene diversity and metabolic potential of the microbial community in an estuary-shelf environment. Front. Microbiol. 8, 1153. 10.3389/fmicb.2017.01153. 28680420 PMC5478683

[R77] Watanabe K, Komatsu N, Ishii Y, Negishi M, 2009. Effective isolation of bacterioplankton genus *Polynucleobacter* from freshwater environments grown on photochemically degraded dissolved organic matter. FEMS Microbiol. Ecol. 67, 57–68. 10.1111/j.1574-6941.2008.00606.x. 19049496

[R78] Wood DE, Lu J, Langmead B, 2019. Improved metagenomic analysis with Kraken 2. Genome Biol. 20, 257. 10.1186/s13059-019-1891-0. 31779668 PMC6883579

[R79] Woodcroft B. 2020. SingleM v0.13.2. Available at: https://github.com/wwood/singlem (accessed 3 March 2021).

[R80] World Health Organization (WHO). Drinking-Water. Available online: https://www.who.int/news-room/fact-sheets/detail/drinking-water (accessed on 7 July 2021).

[R81] Wu YW, Simmons BA, Singer SW, 2016. MaxBin 2.0: an automated binning algorithm to recover genomes from multiple metagenomic datasets. Bioinformatics 32, 605–607. 10.1093/bioinformatics/btv638. 26515820

[R82] Xue C, Lin H, Zhu X, Liu J, Zhang Y, Rowley G, Todd J, Li M, Zhang X, 2021. DiTing: a pipeline to infer and compare biogeochemical pathways from metagenomic and metatranscriptomic data. Front. Microbiol. 12, 698286 10.3389/fmicb.2021.698286. PMC8367434 34408730

[R83] Zacheus O, 2013. Summary of major water distribution areas reporting to the European Commission on the control and quality of domestic water in 2018. Unit of Expert Microbiology. Health and Department of Welfare, Kuopio, Finland. Available online: https://www.valvira.fi/documents/14444/10176523/Talousvesiyhteenveto+2018.pdf.

[R84] Zhang Y, Liu W−T, 2019. The application of molecular tools to study the drinking water microbiome - current understanding and future needs. Crit. Rev. Environ. Sci. Technol. 49, 1188–1235. 10.1080/10643389.2019.1571351.

[R85] Zhou Z, Tran PQ, Breister AM, Liu Y, Kieft K, Cowley ES, Karaoz U, Anantharaman K. 2020. METABOLIC: high-throughput profiling of microbial genomes for functional traits, biogeochemistry, and community-scale metabolic networks. bioRxiv 761643. doi: 10.1101/761643. PMC8851854 35172890

