# Supplementary material for: A comparative analysis employing a gene- and genome-centric metagenomic approach reveals changes in composition, function, and activity in waterworks with different treatment processes and source water in Finland
Source: Water Res. Author manuscript; Available in PMC 2023 Apr 24. (PMC10125003; doi:10.1016/j.watres.2022.119495)
Supplement: Supplementary Material [file NIHMS1861462-supplement-Supplementary_Material.docx]

**Supporting Information**

**A comparative analysis employing a** **gene- and genome-centric metagenomic approach reveal changes in composition, function, and activity in waterworks with different treatment processes and source water in Finland**

Vicente Gomez-Alvarez ^a,*^, Sallamaari Siponen ^b,c^, Ari Kauppinen ^b,#^, Anna-Maria Hokajärvi ^b^, Ananda Tiwari ^b,d^, Anniina Sarekoski ^b,d^, Ilkka T. Miettinen ^b^, Eila Torvinen ^c^, and Tarja Pitkänen ^b,d^

^a^U.S. Environmental Protection Agency, Office of Research and Development, Cincinnati, Ohio 45268 USA

^b^Finnish Institute for Health and Welfare, Dept. of Health Security, FI-70701 Kuopio, Finland

^c^University of Eastern Finland, Dept. of Environmental and Biological Sciences, FI-70211 Kuopio, Finland

^d^University of Helsinki, Faculty of Veterinary Medicine, Dept. Food Hygiene and Environmental Health, FI-00790 Helsinki, Finland

^#^Current address: Finnish Food Authority, Laboratory and Research Division, Animal Health Diagnostic Unit, Helsinki, Finland

**Table of Contents**

|  |  | **Page** |
| --- | --- | --- |
| **Supplementary Materials and Methods** | | S2 |
| **Tables** |  |  |
| Table S1 | Summary of DWDS characteristics | S7 |
| Table S2 | Cold water quality parameters in DWDS | S8 |
| Table S3 | Summary statistics of MAGs | S9 |
| Table S4 | Distribution and coverage of MAGs | S14 |
| **Figures** |  |  |
| Figure S1 | Flowchart of research methodology | S17 |
| Figure S2 | Metagenomic taxonomic profile of Site A (ND) | S18 |
| Figure S3 | Metagenomic taxonomic profile of Site B (ND) | S19 |
| Figure S4 | Metagenomic taxonomic profile of Site C (CHL) | S20 |
| Figure S5 | Metagenomic taxonomic profile of Site E (CHL) | S21 |
| Figure S6 | Metagenomic taxonomic profile of Site D (CHM) | S22 |
| Figure S7 | Bacterial 16S rRNA gene diversity and taxonomy | S23 |
| Figure S8 | Archaeal 16S rRNA gene diversity and taxonomy | S24 |
| Figure S9 | Metabolic potential of five DWDS | S25 |
| Figure S10 | Cluster analysis of metabolic profiles from five DWDS | S26 |
| Figure S11 | Pathways involved in the Carbon cycle | S27 |
| Figure S12 | Taxonomic distribution of MAGs | S28 |
| Figure S13 | Differences in metabolic pathways of Site A | S29 |
| Figure S14 | Taxonomic and functional profiles of Site A | S30 |
| Figure S15 | Differences in metabolic pathways of Site D | S32 |
| Figure S16 | Taxonomic and functional profiles of Site D | S33 |
| Figure S17 | Metabolic and biogeochemical profiles in MAGs | S35 |
| **References** |  | S36 |

**SUPPLEMENTARY MATERIALS and METHODS**

*Sample collection*

A volume of 100 L from the cold-water system was collected and filtered on-site using a dead-end ultrafiltration method (DEUF) using a Rexeed-25A hollow-fiber polysulfone filter (Asahi Kasei Medical Co., Ltd., Tokyo, Japan) attached to a tap (Inkinen et al., 2021). The average flow of water during sample collection was 3 L/min. Water samples from chlorinated waterworks were treated with a 1% solution of sodium thiosulphate. Cells were eluted from capsules to 500 ml with a backflush solution containing 0.01% sodium polyphosphate, 0.001% Antifoam Y, and 0.5% Tween-80 in sterile water. The dead-end ultrafiltration eluate was concentrated by filtrating a volume of 350 ml through a 0.22 µm Express PLUS membrane filter (Merck KGaA, Darmstadt, Germany). Filters were stored and transported on ice for elution and DNA/RNA processing in the laboratory. The membranes were frozen at -75 °C or lower before the extraction of nucleic acids.

*DNA and RNA extraction, complementary DNA (cDNA) synthesis, and sequencing*

DNA and RNA were extracted from the Express PLUS membranes as described earlier (Inkinen et al., 2019). Briefly, total nucleic acids were extracted to a final elution volume of 100 μL using a Chemagic DNA Plant kit (Perkin Elmer, Waltham, USA) following the manufacturer’s instructions without removing the RNA. A subsample of the extracted nucleic acids (i.e., DNA) were treated with RNase A (Thermo Fischer Scientific, Waltham, USA). The RNA was purified from a subsample of the extracted nucleic acids using an Ambion TURBO DNA-freeTM kit (Life Technologies, Carlsbad, USA) followed by reverse transcription to cDNA of triplicate RNA subsamples using the Invitrogen Superscript III First-Strand Synthesis System (Thermo Fisher Scientific, Waltham, USA). The cDNA subsamples were combined and purified with GenElute PCR clean-up kit (Sigma-Aldrich, Darmstadt, Germany). Nucleic acid concentrations were measured with a with a Qubit minifluorometer using the dsDNA HS and RNA HS Assay kits (Thermo Fisher Scientific, Waltham, USA). The extraction series included a positive extraction and negative process control. DNA and cDNA samples were sent to GATC BioTech AG (European Genome and Diagnostics Centre, Germany) for sequencing.

*PCR amplification and sequencing*

Barcoded primers 341F-785R (Klindworth et al., 2013) targeting the highly variable V3-4 regions of the 16S rRNA gene were used for PCR amplification of the bacteria domain. The primers A340F (Gantner et al., 2011) and 915R (Stahl and Amann, 1991) designed specifically for archaea, were used in a tagged amplicon PCR. PCR reactions were conducted as described earlier (Inkinen et al., 2019) and included ca. 1-10 ng of nucleic acids. Illumina libraries were constructed using a ca. 100 ng purified amplicon pool and gel electrophoresis was used for pooling and size selection. Sequencing was performed on an Illumina MiSeq platform that produced 300 base pair (bp) paired-end reads (Illumina, Inc., San Diego, USA).

*Bacteria 16S reads processing*

Reads were analyzed using the software mothur (Schloss et al., 2009) v1.45.2 and screened following the procedure described in Gomez-Alvarez et al. (2016)**.** Briefly, fastq files with forward and reverse reads were used to form contigs. Reads were screened and removed if they (i) had a minimum length of 400 bp and maximum of 428 bp, (ii) contained ambiguous bases (N's), (iii) contained homopolymers greater than 7 bases, (iv) were identified as chimera, or (v) were classified as unknown, Archaea, Chloroplasts, or Mitochondria. Reads were aligned against the SILVA SEED (Quast et al., 2013) release 123 reference data set and grouped with 97% sequence identity as the cut-off point for each Operational Taxonomic Unit (OTU). Taxonomic classification was obtained using the GTDB r95 reference database (Parks et al., 2020). Bacteria database and taxonomic outline for the GTDB hierarchies were downloaded (Alishum 2021) and formatted for the purpose of using with the software mothur. Prior to community analysis, samples were rarefied to the smallest data set of 4,450 bacteria reads.

*Archaea 16S reads processing*

Reads were analyzed using the software mothur (Schloss et al., 2009) v1.45.2 and screened following the procedure described in Gomez-Alvarez et al. (2016)**.** Briefly, fastq files with forward and reverse reads were used to form contigs. Reads were screened and removed if they (i) had a minimum length of 400 bp and maximum of 544 bp, (ii) contained greater than 7 ambiguous bases (N's), (iii) contained homopolymers greater than 7 bases, (iv) were identified as chimera, or (v) were classified as unknown, Bacteria, Chloroplasts, or Mitochondria. Reads were aligned against the SILVA SEED (Quast et al., 2013) release 123 reference data set and grouped with 97% sequence identity as the cut-off point for each Operational Taxonomic Unit (OTU). Taxonomic classification was obtained using the GTDB r95 reference database (Parks et al., 2020). Archaea database and taxonomic outline for the GTDB hierarchies were downloaded (Alishum 2021) and formatted for the purpose of using with the software mothur. Prior to community analysis, samples were rarefied to the smallest data set of 1,000 bacteria reads.

*Metabolic inference of metagenomic and metatranscriptomic reads*

Metabolic reconstruction and the relative abundance of genes involved in key biogeochemical pathways were determined by DiTing v0.9 (Xue et al., 2021). DiTing infer and compare the prevalence of genes and pathways of Carbon, Nitrogen, Dimethylsulfoniopropionate (DMSP), and Sulfur biogeochemical cycles and consists of Open Reading Frame (ORF) prediction, mapping, gene annotation, and visualization of biogeochemical cycling. The input source is a set of metagenomic and/or metatranscriptomic (rRNA removed) reads and assembled contigs with default parameters. Genes are predicted and translated from the assembled contigs by Prodigal v2.6.3 (Hyatt et al., 2010) with the ‘-p meta’ option. The translated protein sequences are queried against the KOfam database (HMM database of KEGG Orthologs [v.2021-10-03 KEGG release 100.0]) (Aramaki et al., 2019) using *hmmsearch* implemented within HMMER v3.3.2 (Eddy 2011). An output table with the normalized relative abundance and annotation of genes was used to produced sketch plots for comparing the biogeochemical pathways in different samples. Normalized relative abundance was calculated through dividing the relative abundance of a pathway in an individual sample by the sum of this pathway’s relative abundance in all samples (Xue et al., 2021).

Hierarchical classification (BRITE, KO, modules, pathways) of metabolic functions was obtained using the online tool FuncTree2 (Darzi et al., 2019). A table with relative abundances of KO number was mapped to KEGG pathways to generate a hierarchical classification file. The abundance was calculated for each sample as the sum of the abundances of the genes that composed each hierarchical classification.

*Metagenome-Assembled Genomes (MAGs)*

Prior to assembly, metagenomes were pooled by location, and *de novo* co-assembly were performed with filtered reads using the metagenomic assembler MEGAHIT v1.2.9 (Li et al., 2016) with default settings and minimum contig size of 1500 nucleotides. Contigs data was binned using anvi’o v6.1 (Eren et al., 2015), following the online workflow Anvi'o User Tutorial for Metagenomic (http://merenlab.org/2016/06/22/anvio-tutorial-v2) with some modifications. Default parameters were used for all software unless otherwise specified. Briefly, the fasta file (contigs) was reformatted with simple deflines using the module *anvi-script-reformat-fasta*, and the reads were mapped to the contigs with Bowtie2 v2.3.5.1 (Langmead and Salzberg 2012). The resulting sam file was converted and sorted to a bam file with samtools v1.10 (Li et al., 2009). Binning was performed with MaxBin2 v.2.2.6 (Wu et al., 2016) and with MetaBat2 v.2.15 (Kang et al., 2019). Subsequently, the bins were optimized and dereplicated using the tool DAS Tool v 1.1.2 (Sieber et al., 2018). Bins were consolidated with the module *metawrap bin_refinement* using MetaWRAP v1.3.2 (Uritskiy et al., 2018) with a minimum completion of 50%. Bins were continuously assessed for completeness and contamination with CheckM v1.1.2 (Parks et al., 2015). Clusters were manually refined, and contaminants removed using the tools RefineM v0.1.1 (Parks et al., 2017) and MAGpurify v2.1.2 (Nayfach et al., 2019). Bins were reassembled with the module *metawrap reassemble_bins* using MetaWRAP (Uritskiy et al., 2018). Coverage and relative abundance in the community of bins (i.e., MAGs) was assessed with CheckM v1.1.2 using the modules *checkm* *coverage* and *checkm* *profile* (Parks et al., 2015). Relative abundance is defined as the proportion of a bin relative to the number of reads mapped to assembled contigs and adjusted for the size of the bin.

*Selection and Analysis of MAGs*

MAGs with ≥50% completeness, ≤10% contamination, and ≤10% strain heterogeneity were selected for downstream analysis (Bowers et al., 2017). In the present study, high-quality draft genomes were identified as MAGs with >90% completeness and < 5% contamination. Medium-high quality MAGs were identified as >70% completeness and < 5% contamination. In addition, the presence and completeness of encoded rRNAs and tRNAs were omitted as an additional metric for assembly quality (Bowers et al., 2017). In metagenomes assemblies (e.g., MAGs) the recovery of rRNA is poor, which is not unusual due to the difficulty of assembling these regions (Yuan et al., 2015). Contigs were annotated with Prokka v1.14.5 (Seemann 2014). Taxonomy was refined and confirmed by using GTDB-Tk v1.4.1 (Chaumeil et al., 2019), a software for assigning objective taxonomic classifications to genomes based on the Genome Taxonomy Database (GTDB) release 95 (Parks et al., 2020). Bins were de-replicated to generate a non-redundant set of MAGs using dRep v3.2.0 (Olm et al., 2017) with default parameters. A phylogenetic tree of de-replicated MAGs was created with PhyloPhlAn v3.0.2 (Asnicar et al., 2020) using RAxML version 8.2.12 (Stamatakis et al., 2014). The tree was visualized with FigTree v1.4.4 (Rambaut 2018).

**Table S1.** Characteristics of the DWDSs (adapted from Ikonen et al., 2017).

| **DWDS** |  | **Raw water source** |  | **Treatment methods** |  | **Disinfection** |
| --- | --- | --- | --- | --- | --- | --- |
| **A** |  | Artificial groundwater |  | Aeration, lime stabilization, flocculation, clarification, addition of sulfuric acid, sand filtration |  | No disinfection |
|  |  |  |  |  |  |  |
| **B** |  | Artificial groundwater |  | Aeration, lime stabilization, flocculation, clarification, sand filtration |  | No disinfection |
|  |  |  |  |  |  |  |
| **C** |  | Surface water |  | Ferric sulfate coagulation, flotation, sand filtration, activated carbon filtration |  | UV-light, ClO_2_, Cl |
|  |  |  |  |  |  |  |
| **D** |  | Surface water |  | Ferric sulfate coagulation, clarification, sand filtration, ozonation, activated carbon filtration |  | UV-light, NH_2_Cl |
|  |  |  |  |  |  |  |
| **E** |  | Groundwater |  | Aeration, limestone filtration |  | UV-light, NaOCl |

**Table S2.** Physico-chemical water quality and nutrients in cold drinking water systems (adapted from Inkinen et al., 2019).

|  |  | |  |  | |  | **DWDS** | |  |  | |  |  | |
| --- | --- | --- | --- | --- | --- | --- | --- | --- | --- | --- | --- | --- | --- | --- |
|  | **A** | |  | **B** | |  | **C** | |  | **D** | |  | **E** | |
|  | **avg** | **range** |  | **avg** | **range** |  | **avg** | **range** |  | **avg** | **range** |  | **avg** | **range** |
| **Turbidity** (NTU) | 0.14 | (0.01 - 0.36) |  | 0.11 | (0.04 - 0.72) |  | 0.04 | (0.01 - 0.11) |  | 0.08 | (0.02 - 0.34) |  | 0.02 | (0.01 - 0.05) |
| **Temperature** (°C) | 10.8 | (5.5 - 18.1) |  | 9.6 | (5.8 - 12.7) |  | 10.3 | (1.7 - 20.1) |  | 9.7 | (5 - 16.6) |  | 6.4 | (4 - 10.6) |
| **Cu** (mg/L) | 0.04 | (0.02 - 0.08) |  | 0.04 | (0.02 - 0.08) |  | 0.05 | (0.02 - 0.11) |  | 0.04 | (0.02 - 0.08) |  | 0.03 | (0.02 - 0.06) |
| **Al** (mg/L) | 0.01 | (0 - 0.03) |  | 0.01 | (0 - 0.02) |  | 0.01 | (0 - 0.04) |  | 0.01 | (0 - 0.05) |  | 0.01 | (0 - 0.03) |
| **Mn** (mg/L) | 0.02 | (0 - 0.12) |  | 0.01 | (0 - 0.01) |  | 0.01 | (0 - 0.05) |  | 0.01 | (0 - 0.04) |  | 0 | (0 - 0.01) |
| **Fe** (mg/L) | 0.07 | (0.03 - 0.16) |  | 0.05 | (0.01 - 0.09) |  | 0.03 | (0.01 - 0.12) |  | 0.09 | (0.05 - 0.14) |  | 0.03 | (0.01 - 0.17) |
| **EC** (μS/cm) | 217 | (163 - 252) |  | 214 | (160 - 240) |  | 151 | (145 - 155) |  | 147 | (134 - 194) |  | 102 | (94 - 160) |
| **pH** | 8.3 | (7.1 - 8.6) |  | 8.3 | (7.2 - 8.8) |  | 8.3 | (7.8 – 9.0) |  | 7.9 | (7.1 - 8.7) |  | 8.0 | (6.2 - 8.9) |
| **Total Cl** (mg/L) | - | - |  | - | - |  | 0.31 | (0.07 - 0.61) |  | 0.09 | (0.02 - 0.43) |  | 0.19 | (0.06 - 0.48) |
| **Free Cl** (mg/L) | - | - |  | - | - |  | 0.29 | (0.03 - 0.97) |  | 0.08 | (0.02 - 0.23) |  | 0.16 | (0.01 - 0.33) |
| **AOC** (μg-C/L) | 89 | (37 - 175) |  | 82 | (39 - 192) |  | 143 | (74 - 254) |  | 138 | (58 - 257) |  | 73 | (9 - 266) |
| **MAP** (μg-PO_4_-P/L) | 0.8 | (0.4 - 1.3) |  | 0.8 | (0.4 - 1.6) |  | 0.2 | (0.04 - 0.9) |  | 0.2 | (0.1 - 0.5) |  | 4.8 | (3.3 - 6.9) |

AOC: assimilable organic carbon, MAP: microbially available phosphorus, EC: electric conductivity

**Table S3.** Summary statistics of metagenome-assembled genomes (MAGs).

| **MAG** | **DWDS** |  | **Assembly** (bp) | |  | **Quality** (%) | |  | **Taxonomic placement** | | | |
| --- | --- | --- | --- | --- | --- | --- | --- | --- | --- | --- | --- | --- |
|  |  |  | **Size** | **Contig N_50_** |  | **Comp.** | **Cont.** |  | **Domain** | **Phylum** | **Class** | **Genus** |
| Bin.31 A | A |  | 581,332 | 44,374 |  | 68.9 | 0.0 |  | Archaea | *Nanoarchaeota* | *Nanoarchaeia* | ASMP01 |
| Bin.06 B | B |  | 3,808,249 | 29,672 |  | 90.5 | 2.6 |  | Bacteria | *Acidobacteriota* | *Blastocatellia* | OLB17 |
| Bin.21 E | E |  | 3,790,919 | 350,600 |  | 96.6 | 2.6 |  | Bacteria | *Acidobacteriota* | *Blastocatellia* | OLB17 |
| Bin.01 B | B |  | 2,362,387 | 68,271 |  | 89.0 | 0.1 |  | Bacteria | *Bacteroidota* | *Bacteroidia* | CG1-02-35-72 |
| Bin.09 A | A |  | 3,642,312 | 62,886 |  | 98.8 | 0.0 |  | Bacteria | *Bacteroidota* | *Bacteroidia* | PHOS-HE28 |
| Bin.23 E | E |  | 3,339,134 | 94,480 |  | 90.7 | 0.9 |  | Bacteria | *Bdellovibrionota* | *Bacteriovoracia* | UBA6144 |
| Bin.31 B | B |  | 4,161,557 | 42,109 |  | 91.6 | 2.5 |  | Bacteria | *Bdellovibrionota* | *Bdellovibrionia* | ND |
| Bin.37 B | B |  | 3,010,708 | 6,794 |  | 82.7 | 0.1 |  | Bacteria | *Bdellovibrionota* | *Bdellovibrionia_A* | ND |
| Bin.14 E | E |  | 1,564,038 | 36,403 |  | 92.7 | 0.0 |  | Bacteria | *Chloroflexota* | *Dehalococcoidia* | UBA2162 |
| Bin.10 E | E |  | 3,593,773 | 3,277 |  | 51.0 | 1.0 |  | Bacteria | *Cyanobacteria* | *Vampirovibrionia* | Ga0077546 |
| Bin.16 D | D |  | 6,313,525 | 6,036 |  | 77.8 | 3.1 |  | Bacteria | *Cyanobacteria* | *Vampirovibrionia* | ND |
| Bin.08 C | C |  | 4,353,021 | 3,630,122 |  | 90.6 | 0.6 |  | Bacteria | *Cyanobacteria* | *Vampirovibrionia* | ND |
| Bin.11 C | C |  | 6,376,314 | 18,314 |  | 85.8 | 2.3 |  | Bacteria | *Cyanobacteria* | *Vampirovibrionia* | ND |
| Bin.13 B | B |  | 1,937,564 | 3,159 |  | 53.4 | 3.6 |  | Bacteria | *Desulfobacterota_B* | *Binatia* | UBA9968 |
| Bin.02 A | A |  | 3,589,809 | 16,430 |  | 87.6 | 2.8 |  | Bacteria | *Nitrospirota* | *Nitrospiria* | *Nitrospira* |
| Bin.20 B | B |  | 2,560,451 | 12,156 |  | 58.9 | 0.1 |  | Bacteria | *Nitrospirota* | *Nitrospiria* | *Nitrospira* |
| Bin.11 D | D |  | 3,523,481 | 302,535 |  | 85.4 | 4.1 |  | Bacteria | *Nitrospirota* | *Nitrospiria* | *Nitrospira* |
| Bin.16 B | B |  | 2,665,310 | 10,134 |  | 89.0 | 3.2 |  | Bacteria | *Nitrospirota* | *Nitrospiria* | ND |
| Bin.10 A | A |  | 2,666,197 | 28,386 |  | 95.9 | 0.0 |  | Bacteria | *Nitrospirota* | *Nitrospiria_A* | 9FT-COMBO-42-15 |
| Bin.15 B | B |  | 2,704,405 | 25,264 |  | 96.5 | 1.8 |  | Bacteria | *Nitrospirota* | *Nitrospiria_A* | 9FT-COMBO-42-15 |
| Bin.05 E | E |  | 1,392,282 | 6,244 |  | 69.0 | 0.1 |  | Bacteria | *Omnitrophota* | *Koll11* | 2-01-FULL-45-10 |
| Bin.32 B | B |  | 640,681 | 3,229 |  | 54.8 | 0.1 |  | Bacteria | *Omnitrophota* | *Koll11* | 2-02-FULL-50-19 |
| Bin.12 E | E |  | 1,548,076 | 61,201 |  | 90.1 | 1.1 |  | Bacteria | *Omnitrophota* | *Koll11* | UBA6249 |
| Bin.18 B | B |  | 1,831,764 | 26,742 |  | 93.0 | 3.2 |  | Bacteria | *Omnitrophota* | *Koll11* | ND |
| Bin.19 A | A |  | 2,146,045 | 24,909 |  | 88.4 | 2.2 |  | Bacteria | *Omnitrophota* | *Koll11* | ND |
| Bin.43 B | B |  | 1,276,493 | 3,292 |  | 60.3 | 2.2 |  | Bacteria | *Omnitrophota* | *Koll11* | ND |
| Bin.06 E | E |  | 1,698,064 | 103,648 |  | 91.6 | 2.2 |  | Bacteria | *Omnitrophota* | *Koll11* | ND |
| Bin.09 E | E |  | 1,056,595 | 31,563 |  | 66.6 | 1.0 |  | Bacteria | *Patescibacteria* | *ABY1* | ND |
| Bin.21 B | B |  | 1,132,974 | 25,381 |  | 61.7 | 1.0 |  | Bacteria | *Patescibacteria* | *Doudnabacteria* | ND |
| Bin.34 A | A |  | 1,359,246 | 89,817 |  | 70.8 | 0.0 |  | Bacteria | *Patescibacteria* | *Gracilibacteria* | ND |
| Bin.18 A | A |  | 987,999 | 56,966 |  | 73.1 | 0.0 |  | Bacteria | *Patescibacteria* | *Microgenomatia* | 2-02-FULL-36-13 |
| Bin.45 B | B |  | 896,050 | 76,615 |  | 64.2 | 0.0 |  | Bacteria | *Patescibacteria* | *Microgenomatia* | ND |
| Bin.28 A | A |  | 818,596 | 4,564 |  | 67.6 | 0.0 |  | Bacteria | *Patescibacteria* | *Microgenomatia* | ND |
| Bin.14 D | D |  | 580,559 | 39,548 |  | 75.1 | 0.0 |  | Bacteria | *Patescibacteria* | *Paceibacteria* | 1-14-0-10-45-20 |
| Bin.23 A | A |  | 678,823 | 10,484 |  | 77.3 | 3.0 |  | Bacteria | *Patescibacteria* | *Paceibacteria* | 1-14-0-10-45-20 |
| Bin.25 B | B |  | 744,695 | 13,690 |  | 81.2 | 0.0 |  | Bacteria | *Patescibacteria* | *Paceibacteria* | 1-14-0-10-45-20 |
| Bin.28 B | B |  | 638,657 | 6,520 |  | 60.3 | 1.9 |  | Bacteria | *Patescibacteria* | *Paceibacteria* | 2-01-FULL-46-13 |
| Bin.17 A | A |  | 912,859 | 146,538 |  | 78.6 | 0.0 |  | Bacteria | *Patescibacteria* | *Paceibacteria* | C7867-006 |
| Bin.21 D | D |  | 634,152 | 32,703 |  | 78.4 | 0.0 |  | Bacteria | *Patescibacteria* | *Paceibacteria* | GWA1-54-10 |
| Bin.20 A | A |  | 611,924 | 144,332 |  | 62.2 | 1.7 |  | Bacteria | *Patescibacteria* | *Paceibacteria* | GWA1-54-10 |
| Bin.27 A | A |  | 676,141 | 20,057 |  | 68.1 | 8.4 |  | Bacteria | *Patescibacteria* | *Paceibacteria* | GWA1-54-10 |
| Bin.18 D | D |  | 917,421 | 89,436 |  | 62.9 | 0.9 |  | Bacteria | *Patescibacteria* | *Paceibacteria* | OLB19 |
| Bin.11 A | A |  | 655,439 | 9,315 |  | 63.2 | 1.6 |  | Bacteria | *Patescibacteria* | *Paceibacteria* | SURF-56 |
| Bin.21 A | A |  | 1,074,189 | 596,029 |  | 59.8 | 0.0 |  | Bacteria | *Patescibacteria* | *Paceibacteria* | UBA10103 |
| Bin.22 B | B |  | 1,076,087 | 638,143 |  | 60.7 | 0.0 |  | Bacteria | *Patescibacteria* | *Paceibacteria* | UBA10103 |
| Bin.36 B | B |  | 847,571 | 89,832 |  | 65.7 | 0.0 |  | Bacteria | *Patescibacteria* | *Paceibacteria* | UBA10103 |
| Bin.39 B | B |  | 697,467 | 88,863 |  | 72.2 | 2.3 |  | Bacteria | *Patescibacteria* | *Paceibacteria* | UBA11704 |
| Bin.35 B | B |  | 714,251 | 52,077 |  | 69.8 | 1.7 |  | Bacteria | *Patescibacteria* | *Paceibacteria* | UBA11704 |
| Bin.32 A | A |  | 382,512 | 5,469 |  | 54.9 | 1.1 |  | Bacteria | *Patescibacteria* | *Paceibacteria* | UBA5004 |
| Bin.29 B | B |  | 444,070 | 15,381 |  | 59.8 | 1.1 |  | Bacteria | *Patescibacteria* | *Paceibacteria* | UBA5004 |
| Bin.26 A | A |  | 516,105 | 57,370 |  | 72.4 | 0.0 |  | Bacteria | *Patescibacteria* | *Paceibacteria* | UBA5004 |
| Bin.47 B | B |  | 544,798 | 477,553 |  | 74.1 | 0.0 |  | Bacteria | *Patescibacteria* | *Paceibacteria* | UBA5004 |
| Bin.13 A | A |  | 484,370 | 83,928 |  | 72.4 | 0.0 |  | Bacteria | *Patescibacteria* | *Paceibacteria* | UBA5004 |
| Bin.19 B | B |  | 470,494 | 132,703 |  | 70.7 | 0.0 |  | Bacteria | *Patescibacteria* | *Paceibacteria* | UBA5004 |
| Bin.29 A | A |  | 530,184 | 99,924 |  | 77.6 | 0.0 |  | Bacteria | *Patescibacteria* | *Paceibacteria* | UBA5004 |
| Bin.11 B | B |  | 508,206 | 153,229 |  | 67.6 | 0.0 |  | Bacteria | *Patescibacteria* | *Paceibacteria* | UBA5004 |
| Bin.12 B | B |  | 453,291 | 27,003 |  | 56.4 | 1.7 |  | Bacteria | *Patescibacteria* | *Paceibacteria* | UBA5004 |
| Bin.46 B | B |  | 446,092 | 12,635 |  | 54.6 | 0.0 |  | Bacteria | *Patescibacteria* | *Paceibacteria* | UBA9973 |
| Bin.19 E | E |  | 616,459 | 171,938 |  | 78.3 | 0.0 |  | Bacteria | *Patescibacteria* | *Paceibacteria* | UBA9973 |
| Bin.05 A | A |  | 648,071 | 636,261 |  | 75.0 | 0.0 |  | Bacteria | *Patescibacteria* | *Paceibacteria* | UBA9973 |
| Bin.41 B | B |  | 634,691 | 634,691 |  | 75.0 | 0.0 |  | Bacteria | *Patescibacteria* | *Paceibacteria* | UBA9973 |
| Bin.33 A | A |  | 712,297 | 170,744 |  | 56.8 | 0.0 |  | Bacteria | *Patescibacteria* | *Paceibacteria* | ND |
| Bin.08 A | A |  | 753,897 | 423,921 |  | 62.6 | 0.9 |  | Bacteria | *Patescibacteria* | *Paceibacteria* | ND |
| Bin.05 B | B |  | 778,157 | 427,104 |  | 62.6 | 0.0 |  | Bacteria | *Patescibacteria* | *Paceibacteria* | ND |
| Bin.44 B | B |  | 545,475 | 7,744 |  | 52.7 | 3.6 |  | Bacteria | *Patescibacteria* | *Paceibacteria* | ND |
| Bin.38 B | B |  | 495,873 | 7,336 |  | 51.2 | 0.0 |  | Bacteria | *Patescibacteria* | *Paceibacteria* | ND |
| Bin.18 E | E |  | 707,042 | 25,457 |  | 71.5 | 0.0 |  | Bacteria | *Patescibacteria* | *Paceibacteria* | ND |
| Bin.35 A | A |  | 509,083 | 7,579 |  | 50.5 | 3.4 |  | Bacteria | *Patescibacteria* | *Paceibacteria* | ND |
| Bin.33 B | B |  | 438,462 | 8,004 |  | 53.0 | 1.6 |  | Bacteria | *Patescibacteria* | *Paceibacteria* | ND |
| Bin.24 B | B |  | 544,894 | 6,300 |  | 54.1 | 6.7 |  | Bacteria | *Patescibacteria* | *Paceibacteria* | ND |
| Bin.30 B | B |  | 1,005,134 | 7,984 |  | 69.2 | 0.5 |  | Bacteria | *Patescibacteria* | *Paceibacteria* | ND |
| Bin.03 A | A |  | 712,999 | 688,526 |  | 87.2 | 0.0 |  | Bacteria | *Patescibacteria* | *Paceibacteria* | ND |
| Bin.40 B | B |  | 626,226 | 164,694 |  | 79.4 | 0.0 |  | Bacteria | *Patescibacteria* | *Paceibacteria* | ND |
| Bin.30 A | A |  | 970,697 | 7,315 |  | 56.0 | 6.7 |  | Bacteria | *Patescibacteria* | *Paceibacteria* | ND |
| Bin.10 B | B |  | 890,844 | 104,917 |  | 61.1 | 5.1 |  | Bacteria | *Patescibacteria* | *Paceibacteria* | ND |
| Bin.03 B | B |  | 750,650 | 3,069 |  | 53.9 | 4.0 |  | Bacteria | *Patescibacteria* | *Paceibacteria* | ND |
| Bin.12 A | A |  | 712,023 | 374,323 |  | 77.9 | 0.0 |  | Bacteria | *Patescibacteria* | *Paceibacteria* | ND |
| Bin.02 B | B |  | 734,697 | 206,612 |  | 74.4 | 0.0 |  | Bacteria | *Patescibacteria* | *Paceibacteria* | ND |
| Bin.22 A | A |  | 788,202 | 6,695 |  | 57.7 | 1.5 |  | Bacteria | *Patescibacteria* | *Paceibacteria* | ND |
| Bin.42 B | B |  | 738,924 | 6,449 |  | 56.9 | 2.5 |  | Bacteria | *Patescibacteria* | *Paceibacteria* | ND |
| Bin.34 B | B |  | 485,285 | 5,831 |  | 59.5 | 0.0 |  | Bacteria | *Patescibacteria* | *Paceibacteria* | ND |
| Bin.08 B | B |  | 1,015,097 | 7,777 |  | 64.8 | 4.5 |  | Bacteria | *Patescibacteria* | *Paceibacteria* | ND |
| Bin.23 B | B |  | 726,707 | 9,943 |  | 55.4 | 0.0 |  | Bacteria | *Patescibacteria* | *Paceibacteria_A* | UBA1568 |
| Bin.15 D | D |  | 1,095,830 | 94,640 |  | 61.0 | 0.0 |  | Bacteria | *Patescibacteria* | *Saccharimonadia* | ND |
| Bin.26 D | D |  | 2,578,652 | 4,212 |  | 57.8 | 2.3 |  | Bacteria | *Planctomycetota* | *Phycisphaerae* | ND |
| Bin.20 E | E |  | 7,747,496 | 24,421 |  | 99.0 | 2.4 |  | Bacteria | *Planctomycetota* | *Planctomycetes* | SXKJ01 |
| Bin.05 C | C |  | 3,988,042 | 5,385 |  | 72.2 | 0.3 |  | Bacteria | *Proteobacteria* | *Alphaproteobacteria* | *Bosea* |
| Bin.17 E | E |  | 1,897,278 | 3,107 |  | 53.3 | 4.0 |  | Bacteria | *Proteobacteria* | *Alphaproteobacteria* | *Brevundimonas* |
| Bin.36 A | A |  | 2,363,778 | 6,447 |  | 78.5 | 2.3 |  | Bacteria | *Proteobacteria* | *Alphaproteobacteria* | Ga0077548 |
| Bin.09 C | C |  | 3,473,426 | 374,437 |  | 90.2 | 1.5 |  | Bacteria | *Proteobacteria* | *Alphaproteobacteria* | *Hyphomicrobium* |
| Bin.11 E | E |  | 3,606,544 | 108,718 |  | 92.0 | 1.5 |  | Bacteria | *Proteobacteria* | *Alphaproteobacteria* | *Hyphomicrobium* |
| Bin.20 D | D |  | 3,644,814 | 12,080 |  | 84.3 | 2.5 |  | Bacteria | *Proteobacteria* | *Alphaproteobacteria* | *Phenylobacterium* |
| Bin.08 D | D |  | 1,930,064 | 106,820 |  | 92.1 | 0.0 |  | Bacteria | *Proteobacteria* | *Alphaproteobacteria* | QFOX01 |
| Bin.03 E | E |  | 4,505,080 | 74,966 |  | 96.4 | 1.8 |  | Bacteria | *Proteobacteria* | *Alphaproteobacteria* | SG-bin9 |
| Bin.06 D | D |  | 3,365,696 | 67,864 |  | 93.1 | 0.5 |  | Bacteria | *Proteobacteria* | *Alphaproteobacteria* | *Sphingomonas* |
| Bin.16 E | E |  | 2,918,764 | 106,438 |  | 95.3 | 0.0 |  | Bacteria | *Proteobacteria* | *Alphaproteobacteria* | *Sphingomonas* |
| Bin.04 C | C |  | 2,159,618 | 10,189 |  | 88.7 | 0.0 |  | Bacteria | *Proteobacteria* | *Alphaproteobacteria* | *Sphingomonas* |
| Bin.22 E | E |  | 2,278,670 | 7,642 |  | 74.5 | 1.0 |  | Bacteria | *Proteobacteria* | *Alphaproteobacteria* | *Sphingopyxis* |
| Bin.01 C | C |  | 2,377,443 | 159,255 |  | 93.3 | 0.0 |  | Bacteria | *Proteobacteria* | *Alphaproteobacteria* | *Sphingorhabdus_B* |
| Bin.13 E | E |  | 2,186,605 | 253,293 |  | 91.5 | 0.2 |  | Bacteria | *Proteobacteria* | *Alphaproteobacteria* | *Sphingorhabdus_B* |
| Bin.04 D | D |  | 2,165,682 | 5,402 |  | 53.5 | 2.1 |  | Bacteria | *Proteobacteria* | *Alphaproteobacteria* | *Sphingorhabdus_B* |
| Bin.06 A | A |  | 2,491,150 | 166,037 |  | 94.5 | 0.0 |  | Bacteria | *Proteobacteria* | *Alphaproteobacteria* | SXRF01 |
| Bin.14 B | B |  | 2,184,561 | 250,697 |  | 94.5 | 0.0 |  | Bacteria | *Proteobacteria* | *Alphaproteobacteria* | SXRF01 |
| Bin.07 C | C |  | 3,238,417 | 95,964 |  | 90.6 | 0.2 |  | Bacteria | *Proteobacteria* | *Alphaproteobacteria* | UBA4765 |
| Bin.27 D | D |  | 2,165,188 | 1,144,420 |  | 93.5 | 0.0 |  | Bacteria | *Proteobacteria* | *Alphaproteobacteria* | UBA6145 |
| Bin.07 D | D |  | 2,578,682 | 26,768 |  | 95.1 | 0.7 |  | Bacteria | *Proteobacteria* | *Alphaproteobacteria* | ND |
| Bin.15 A | A |  | 3,081,276 | 8,078 |  | 72.9 | 0.4 |  | Bacteria | *Proteobacteria* | *Alphaproteobacteria* | ND |
| Bin.26 B | B |  | 3,079,361 | 9,007 |  | 74.3 | 0.9 |  | Bacteria | *Proteobacteria* | *Alphaproteobacteria* | ND |
| Bin.10 C | C |  | 3,085,398 | 4,400 |  | 55.9 | 4.9 |  | Bacteria | *Proteobacteria* | *Alphaproteobacteria* | ND |
| Bin.05 D | D |  | 1,810,166 | 13,208 |  | 72.8 | 0.4 |  | Bacteria | *Proteobacteria* | *Alphaproteobacteria* | ND |
| Bin.23 D | D |  | 2,221,950 | 208,249 |  | 87.5 | 5.2 |  | Bacteria | *Proteobacteria* | *Alphaproteobacteria* | ND |
| Bin.01 D | D |  | 2,172,892 | 19,606 |  | 78.2 | 5.0 |  | Bacteria | *Proteobacteria* | *Alphaproteobacteria* | ND |
| Bin.04 E | E |  | 2,145,226 | 2,056,071 |  | 95.9 | 0.7 |  | Bacteria | *Proteobacteria* | *Alphaproteobacteria* | ND |
| Bin.24 A | A |  | 2,169,982 | 71,329 |  | 93.4 | 0.7 |  | Bacteria | *Proteobacteria* | *Alphaproteobacteria* | ND |
| Bin.27 B | B |  | 1,962,279 | 10,775 |  | 78.5 | 1.3 |  | Bacteria | *Proteobacteria* | *Alphaproteobacteria* | ND |
| Bin.24 D | D |  | 1,936,062 | 12,856 |  | 77.6 | 2.0 |  | Bacteria | *Proteobacteria* | *Alphaproteobacteria* | ND |
| Bin.17 D | D |  | 2,667,023 | 11,399 |  | 86.7 | 0.9 |  | Bacteria | *Proteobacteria* | *Alphaproteobacteria* | ND |
| Bin.25 D | D |  | 3,245,071 | 15,841 |  | 89.9 | 0.2 |  | Bacteria | *Proteobacteria* | *Gammaproteobacteria* | Ga0077527 |
| Bin.02 E | E |  | 3,747,509 | 118,682 |  | 98.4 | 0.6 |  | Bacteria | *Proteobacteria* | *Gammaproteobacteria* | Ga0077527 |
| Bin.08 E | E |  | 1,492,514 | 2,788 |  | 50.4 | 2.4 |  | Bacteria | *Proteobacteria* | *Gammaproteobacteria* | Ga0077554 |
| Bin.04 B | B |  | 1,600,673 | 3,770 |  | 72.4 | 9.7 |  | Bacteria | *Proteobacteria* | *Gammaproteobacteria* | *Gallionella* |
| Bin.13 D | D |  | 2,874,934 | 13,409 |  | 82.9 | 1.4 |  | Bacteria | *Proteobacteria* | *Gammaproteobacteria* | *Hylemonella* |
| Bin.19 D | D |  | 4,180,516 | 77,185 |  | 85.6 | 3.3 |  | Bacteria | *Proteobacteria* | *Gammaproteobacteria* | *Hylemonella* |
| Bin.10 D | D |  | 2,397,418 | 9,453 |  | 66.3 | 2.8 |  | Bacteria | *Proteobacteria* | *Gammaproteobacteria* | *Immundisolibacter* |
| Bin.07 E | E |  | 3,718,166 | 63,974 |  | 93.7 | 0.4 |  | Bacteria | *Proteobacteria* | *Gammaproteobacteria* | KS41 |
| Bin.02 C | C |  | 4,014,699 | 19,299 |  | 86.1 | 1.0 |  | Bacteria | *Proteobacteria* | *Gammaproteobacteria* | *Nevskia* |
| Bin.12 D | D |  | 2,011,779 | 38,056 |  | 87.8 | 0.8 |  | Bacteria | *Proteobacteria* | *Gammaproteobacteria* | *Nitrotoga* |
| Bin.02 D | D |  | 3,308,277 | 190,897 |  | 94.6 | 1.8 |  | Bacteria | *Proteobacteria* | *Gammaproteobacteria* | *Nitrotoga* |
| Bin.03 D | D |  | 2,728,740 | 22,954 |  | 93.6 | 0.3 |  | Bacteria | *Proteobacteria* | *Gammaproteobacteria* | *Nitrotoga* |
| Bin.04 A | A |  | 2,848,478 | 7,990 |  | 70.8 | 2.8 |  | Bacteria | *Proteobacteria* | *Gammaproteobacteria* | PALSA-1006 |
| Bin.07 B | B |  | 2,340,713 | 31,663 |  | 79.9 | 0.8 |  | Bacteria | *Proteobacteria* | *Gammaproteobacteria* | PALSA-1006 |
| Bin.22 D | D |  | 3,171,460 | 15,314 |  | 78.2 | 3.9 |  | Bacteria | *Proteobacteria* | *Gammaproteobacteria* | PHCI01 |
| Bin.03 C | C |  | 2,471,749 | 116,076 |  | 88.0 | 0.0 |  | Bacteria | *Proteobacteria* | *Gammaproteobacteria* | *Polaromonas* |
| Bin.01 E | E |  | 2,455,787 | 221,858 |  | 92.8 | 0.0 |  | Bacteria | *Proteobacteria* | *Gammaproteobacteria* | *Polaromonas* |
| Bin.07 A | A |  | 1,849,920 | 42,961 |  | 81.4 | 0.9 |  | Bacteria | *Proteobacteria* | *Gammaproteobacteria* | *Polynucleobacter* |
| Bin.16 A | A |  | 2,614,797 | 3,414 |  | 51.0 | 0.0 |  | Bacteria | *Proteobacteria* | *Gammaproteobacteria* | *Rhizobacter* |
| Bin.09 D | D |  | 2,202,758 | 14,623 |  | 82.1 | 2.3 |  | Bacteria | *Proteobacteria* | *Gammaproteobacteria* | *Rugosibacter* |
| Bin.06 C | C |  | 4,290,644 | 174,985 |  | 96.9 | 0.3 |  | Bacteria | *Proteobacteria* | *Gammaproteobacteria* | *Undibacterium* |
| Bin.01 A | A |  | 5,209,822 | 5,353 |  | 80.8 | 2.9 |  | Bacteria | *Proteobacteria* | *Gammaproteobacteria* | ND |

Comp.: completeness, Cont.: contamination, ND: not determined.

**Table S4.** Relative abundance and coverage of MAGs.

| **MAG** | **DWDS** |  | **Mapped reads** (%) | **Relative abundance** (%) | **Coverage**  (±SD) |  | **MAG** | **DWDS** |  | **Mapped reads** (%) | **Relative abundance** (%) | **Coverage**  (±SD) |
| --- | --- | --- | --- | --- | --- | --- | --- | --- | --- | --- | --- | --- |
| Bin.31 A | A |  | 0.35 | 0.88 | 6.3 ± 0.5 |  | Bin.45 B | B |  | 0.71 | 1.00 | 7.3 ± 1.0 |
| Bin.06 B | B |  | 2.43 | 0.80 | 5.8 ± 1.0 |  | Bin.28 A | A |  | 0.30 | 0.55 | 3.9 ± 0.7 |
| Bin.21 E | E |  | 1.62 | 1.07 | 25.3 ± 7.6 |  | Bin.08 A | A |  | 1.99 | 3.90 | 28.0 ± 12.1 |
| Bin.01 B | B |  | 2.25 | 1.20 | 8.7 ± 1.0 |  | Bin.05 B | B |  | 2.75 | 4.44 | 32.3 ± 9.4 |
| Bin.09 A | A |  | 2.61 | 1.06 | 7.6 ± 2.5 |  | Bin.22 A | A |  | 0.29 | 0.53 | 3.8 ± 0.8 |
| Bin.23 E | E |  | 0.28 | 0.21 | 4.9 ± 0.5 |  | Bin.42 B | B |  | 0.36 | 0.62 | 4.5 ± 0.9 |
| Bin.31 B | B |  | 2.83 | 0.85 | 6.2 ± 1.2 |  | Bin.11 A | A |  | 0.28 | 0.64 | 4.6 ± 0.7 |
| Bin.37 B | B |  | 1.35 | 0.56 | 4.1 ± 0.7 |  | Bin.18 E | E |  | 0.05 | 0.18 | 4.2 ± 0.4 |
| Bin.14 E | E |  | 0.13 | 0.20 | 4.8 ± 0.7 |  | Bin.33 A | A |  | 0.49 | 1.01 | 7.2 ± 2.2 |
| Bin.10 E | E |  | 0.22 | 0.15 | 3.6 ± 0.7 |  | Bin.44 B | B |  | 0.25 | 0.59 | 4.3 ± 0.9 |
| Bin.08 C | C |  | 8.50 | 5.83 | 89.4 ± 20.9 |  | Bin.08 B | B |  | 0.51 | 0.64 | 4.6 ± 1.3 |
| Bin.11 C | C |  | 0.85 | 0.40 | 6.1 ± 1.0 |  | Bin.24 B | B |  | 0.24 | 0.56 | 4.1 ± 0.7 |
| Bin.16 D | D |  | 0.48 | 0.21 | 4.1 ± 10.0 |  | Bin.34 B | B |  | 0.23 | 0.60 | 4.3 ± 0.7 |
| Bin.13 B | B |  | 0.75 | 0.49 | 3.6 ± 0.7 |  | Bin.23 A | A |  | 0.28 | 0.61 | 4.4 ± 0.8 |
| Bin.02 A | A |  | 3.60 | 1.48 | 10.6 ± 2.7 |  | Bin.25 B | B |  | 0.50 | 0.84 | 6.1 ± 0.9 |
| Bin.20 B | B |  | 2.54 | 1.25 | 9.1 ± 2.1 |  | Bin.14 D | D |  | 0.10 | 0.49 | 9.3 ± 0.8 |
| Bin.11 D | D |  | 8.81 | 7.07 | 135.7 ± 74.1 |  | Bin.20 A | A |  | 0.48 | 1.17 | 8.4 ± 0.5 |
| Bin.16 B | B |  | 1.31 | 0.62 | 4.5 ± 0.9 |  | Bin.27 A | A |  | 0.34 | 0.73 | 5.3 ± 0.7 |
| Bin.10 A | A |  | 1.49 | 0.82 | 5.9 ± 1.1 |  | Bin.21 D | D |  | 0.10 | 0.43 | 8.2 ± 0.9 |
| Bin.15 B | B |  | 1.87 | 0.87 | 6.3 ± 1.1 |  | Bin.30 A | A |  | 0.41 | 0.62 | 4.4 ± 0.8 |
| Bin.18 B | B |  | 1.15 | 0.79 | 5.7 ± 0.8 |  | Bin.35 A | A |  | 0.21 | 0.61 | 4.4 ± 0.6 |
| Bin.12 E | E |  | 0.15 | 0.24 | 5.6 ± 1.4 |  | Bin.10 B | B |  | 0.65 | 0.92 | 6.7 ± 1.0 |
| Bin.19 A | A |  | 1.09 | 0.75 | 5.4 ± 0.8 |  | Bin.33 B | B |  | 0.21 | 0.60 | 4.4 ± 0.6 |
| Bin.06 E | E |  | 0.19 | 0.29 | 6.8 ± 1.3 |  | Bin.30 B | B |  | 0.48 | 0.59 | 4.3 ± 0.6 |
| Bin.43 B | B |  | 0.45 | 0.44 | 3.2 ± 0.7 |  | Bin.03 B | B |  | 0.29 | 0.49 | 3.6 ± 0.9 |
| Bin.05 E | E |  | 0.07 | 0.12 | 2.9 ± 0.6 |  | Bin.18 D | D |  | 0.11 | 0.33 | 6.3 ± 1.2 |
| Bin.32 B | B |  | 0.25 | 0.49 | 3.6 ± 0.7 |  | Bin.21 A | A |  | 0.93 | 1.28 | 9.2 ± 1.9 |
| Bin.09 E | E |  | 0.07 | 0.17 | 4.1 ± 0.6 |  | Bin.22 B | B |  | 1.97 | 2.31 | 16.8 ± 0.4 |
| Bin.21 B | B |  | 0.75 | 0.83 | 6.1 ± 0.8 |  | Bin.36 B | B |  | 0.66 | 0.98 | 7.1 ± 1.3 |
| Bin.34 A | A |  | 0.80 | 0.86 | 6.2 ± 0.3 |  | Bin.39 B | B |  | 0.75 | 1.35 | 9.8 ± 1.9 |
| Bin.18 A | A |  | 0.66 | 0.98 | 7.1 ± 0.8 |  | Bin.35 B | B |  | 0.47 | 0.82 | 6.0 ± 1.0 |
| Bin.05 A | A |  | 1.27 | 2.89 | 20.7 ± 5.6 |  | Bin.23 D | D |  | 0.57 | 0.73 | 14.0 ± 3.5 |
| Bin.41 B | B |  | 1.23 | 2.44 | 17.8 ± 0.0 |  | Bin.24 D | D |  | 0.22 | 0.32 | 6.1 ± 1.1 |
| Bin.46 B | B |  | 0.30 | 0.83 | 6.1 ± 1.3 |  | Bin.01 D | D |  | 0.26 | 0.34 | 6.5 ± 1.4 |
| Bin.19 E | E |  | 0.09 | 0.36 | 8.6 ± 1.0 |  | Bin.05 C | C |  | 0.74 | 0.55 | 8.4 ± 2.0 |
| Bin.38 B | B |  | 0.23 | 0.59 | 4.3 ± 0.6 |  | Bin.09 C | C |  | 15.06 | 12.93 | 198.3 ± 122.1 |
| Bin.17 A | A |  | 0.95 | 1.54 | 11.1 ± 2.0 |  | Bin.11 E | E |  | 1.06 | 0.73 | 17.3 ± 2.4 |
| Bin.03 A | A |  | 0.65 | 1.34 | 9.6 ± 2.8 |  | Bin.10 C | C |  | 0.35 | 0.34 | 5.2 ± 1.9 |
| Bin.40 B | B |  | 0.57 | 1.14 | 8.3 ± 0.9 |  | Bin.07 C | C |  | 20.26 | 18.66 | 286.4 ± 37.2 |
| Bin.29 A | A |  | 0.53 | 1.47 | 10.6 ± 0.3 |  | Bin.36 A | A |  | 1.11 | 0.69 | 5.0 ± 0.9 |
| Bin.26 A | A |  | 0.38 | 1.08 | 7.8 ± 1.1 |  | Bin.03 E | E |  | 0.60 | 0.33 | 7.8 ± 4.9 |
| Bin.13 A | A |  | 0.29 | 0.87 | 6.3 ± 1.3 |  | Bin.06 A | A |  | 2.71 | 1.60 | 11.5 ± 2.9 |
| Bin.32 A | A |  | 0.15 | 0.56 | 4.0 ± 0.8 |  | Bin.14 B | B |  | 3.04 | 1.75 | 12.7 ± 1.6 |
| Bin.47 B | B |  | 0.60 | 1.38 | 10.1 ± 0.4 |  | Bin.08 D | D |  | 0.38 | 0.56 | 10.7 ± 2.3 |
| Bin.11 B | B |  | 0.48 | 1.20 | 8.7 ± 1.6 |  | Bin.04 C | C |  | 0.37 | 0.52 | 8.0 ± 1.5 |
| Bin.29 B | B |  | 0.35 | 0.98 | 7.1 ± 1.4 |  | Bin.06 D | D |  | 2.07 | 1.74 | 33.3 ± 11.0 |
| Bin.19 B | B |  | 0.36 | 0.96 | 7.0 ± 1.0 |  | Bin.16 E | E |  | 0.58 | 0.50 | 11.9 ± 4.2 |
| Bin.12 B | B |  | 0.29 | 0.79 | 5.8 ± 0.9 |  | Bin.22 E | E |  | 0.27 | 0.30 | 7.1 ± 1.8 |
| Bin.12 A | A |  | 0.56 | 1.16 | 8.3 ± 0.0 |  | Bin.01 C | C |  | 19.96 | 25.05 | 384.6 ± 198.9 |
| Bin.02 B | B |  | 0.95 | 1.63 | 11.9 ± 2.7 |  | Bin.04 D | D |  | 0.16 | 0.22 | 4.1 ± 1.0 |
| Bin.28 B | B |  | 0.30 | 0.58 | 4.3 ± 0.7 |  | Bin.13 E | E |  | 5.97 | 6.82 | 161.5 ± 129.9 |
| Bin.23 B | B |  | 0.36 | 0.62 | 4.5 ± 0.8 |  | Bin.17 D | D |  | 0.34 | 0.36 | 6.9 ± 1.0 |
| Bin.15 D | D |  | 0.27 | 0.70 | 13.3 ± 1.0 |  | Bin.19 D | D |  | 2.44 | 1.65 | 31.5 ± 7.4 |
| Bin.26 D | D |  | 0.22 | 0.24 | 4.6 ± 0.9 |  | Bin.13 D | D |  | 0.82 | 0.80 | 15.3 ± 5.9 |
| Bin.20 E | E |  | 0.95 | 0.31 | 7.2 ± 0.9 |  | Bin.22 D | D |  | 1.00 | 0.89 | 17.1 ± 4.9 |
| Bin.17 E | E |  | 0.12 | 0.15 | 3.6 ± 2.6 |  | Bin.03 C | C |  | 12.19 | 14.71 | 225.9 ± 61.4 |
| Bin.20 D | D |  | 0.57 | 0.44 | 8.5 ± 1.8 |  | Bin.01 E | E |  | 68.14 | 69.40 | 1642.0 ± 1425.5 |
| Bin.07 D | D |  | 0.69 | 0.76 | 14.6 ± 2.3 |  | Bin.07 A | A |  | 4.23 | 3.38 | 24.2 ± 6.0 |
| Bin.15 A | A |  | 3.24 | 1.55 | 11.1 ± 2.6 |  | Bin.16 A | A |  | 1.24 | 0.70 | 5.0 ± 1.2 |
| Bin.26 B | B |  | 3.22 | 1.31 | 9.5 ± 2.2 |  | Bin.06 C | C |  | 0.86 | 0.60 | 9.2 ± 2.1 |
| Bin.04 E | E |  | 0.84 | 0.98 | 23.2 ± 7.0 |  | Bin.04 B | B |  | 0.65 | 0.51 | 3.7 ± 0.9 |
| Bin.27 D | D |  | 0.41 | 0.53 | 10.2 ± 2.6 |  | Bin.03 D | D |  | 17.55 | 18.18 | 349.1 ± 111.8 |
| Bin.24 A | A |  | 3.70 | 2.52 | 18.1 ± 2.5 |  | Bin.02 D | D |  | 3.63 | 3.10 | 59.3 ± 20.2 |
| Bin.27 B | B |  | 1.34 | 0.86 | 6.3 ± 1.1 |  | Bin.12 D | D |  | 1.12 | 1.57 | 30.0 ± 5.8 |
| Bin.05 D | D |  | 0.94 | 1.46 | 28.1 ± 5.1 |  | Bin.04 A | A |  | 1.23 | 0.64 | 4.6 ± 1.3 |
| Bin.07 B | B |  | 1.89 | 1.02 | 7.4 ± 2.0 |  | Bin.08 E | E |  | 0.06 | 0.10 | 2.3 ± 0.5 |
| Bin.09 D | D |  | 0.28 | 0.36 | 6.9 ± 2.2 |  | Bin.10 D | D |  | 0.71 | 0.84 | 16.1 ± 4.8 |
| Bin.01 A | A |  | 2.32 | 0.66 | 4.7 ± 1.0 |  | Bin.07 E | E |  | 1.74 | 1.17 | 27.8 ± 7.7 |
| Bin.25 D | D |  | 0.44 | 0.38 | 7.4 ± 1.3 |  | Bin.02 C | C |  | 1.73 | 1.29 | 19.7 ± 5.5 |
| Bin.02 E | E |  | 1.79 | 1.20 | 28.3 ± 4.7 |  |  |  |  |  |  |  |

Percentage of mapped reads: calculated as *(reads mapped to bin / total number of reads mapped to assembly)*.

Relative abundance: estimate the proportion of a bin relative to the number of reads mapped to assembled contigs and adjusted for the size of the bin. Calculated as *[percentage of binned population × (100 - percentage of reads assigned to unbinned contigs)]*.

Percentage of binned population is calculated as *[(percentage of mapped reads / bin size) × (1 / C)]*, where C is the sum of size adjusted bin coverages over all bins.

Sum of size adjusted bin coverages over all bins is calculated as *Σ(percentage of mapped reads / bin size)*.


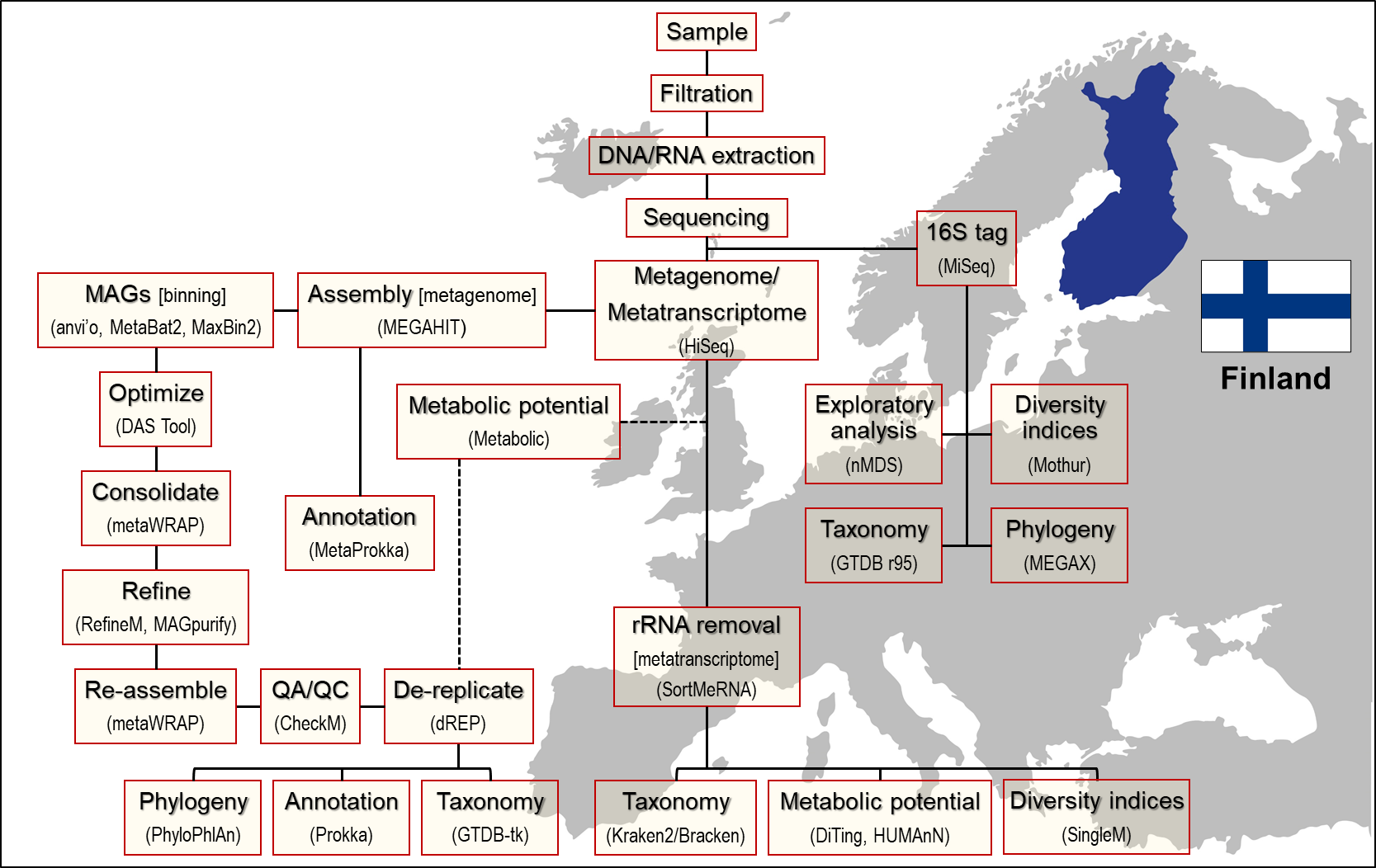


**Figure S1.** **Sampling location and research methodology.** Methods, techniques, and tests are listed in parenthesis.


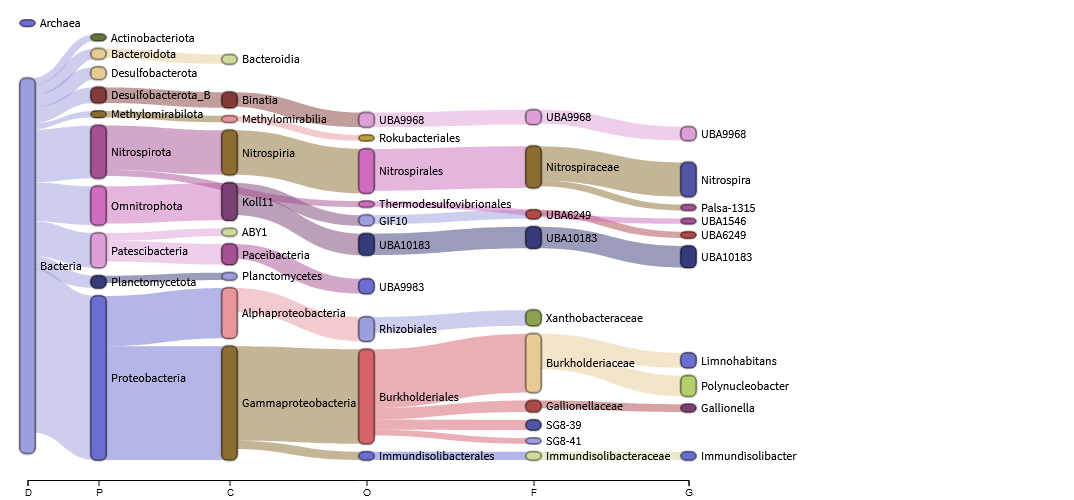


**Figure S2.** **Taxonomic profiling of Site A (ND) microbiota obtained from shotgun metagenomic.** Different colored sidebars in the Sankey diagram show the relative abundance of the microbial community. Flow diagram represents the top 10 taxon at each taxonomic level. Lineage: D, Domain; K, kingdom; P, phylum; C, class; O, order; F, family; G, genus.


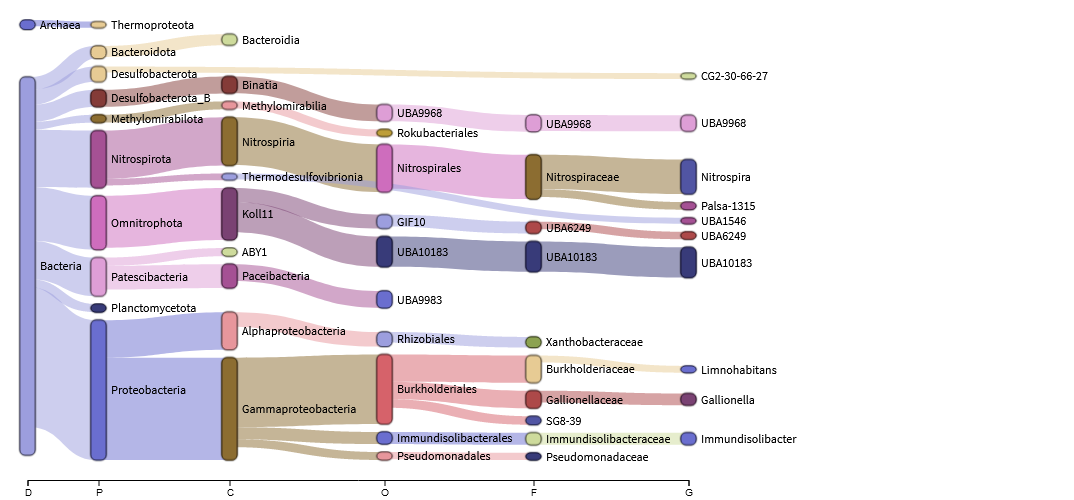


**Figure S3.** **Taxonomic profiling of Site B (ND) microbiota obtained from shotgun metagenomic.** Different colored sidebars in the Sankey diagram show the relative abundance of the microbial community. Flow diagram represents the top 10 taxon at each taxonomic level. Lineage: D, Domain; K, kingdom; P, phylum; C, class; O, order; F, family; G, genus.


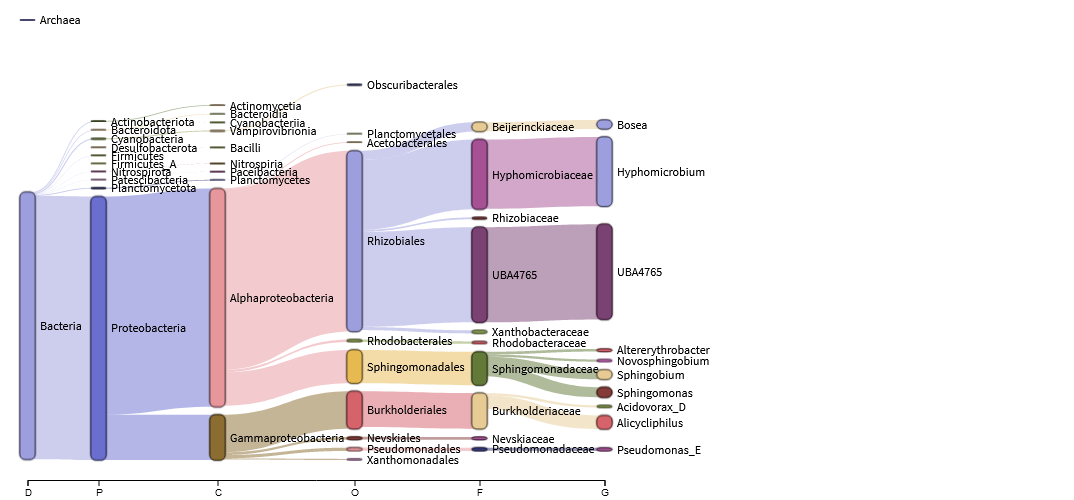


**Figure S4.** **Taxonomic profiling of Site C (CHL) microbiota obtained from shotgun metagenomic.** Different colored sidebars in the Sankey diagram show the relative abundance of the microbial community. Flow diagram represents the top 10 taxon at each taxonomic level. Lineage: D, Domain; K, kingdom; P, phylum; C, class; O, order; F, family; G, genus.


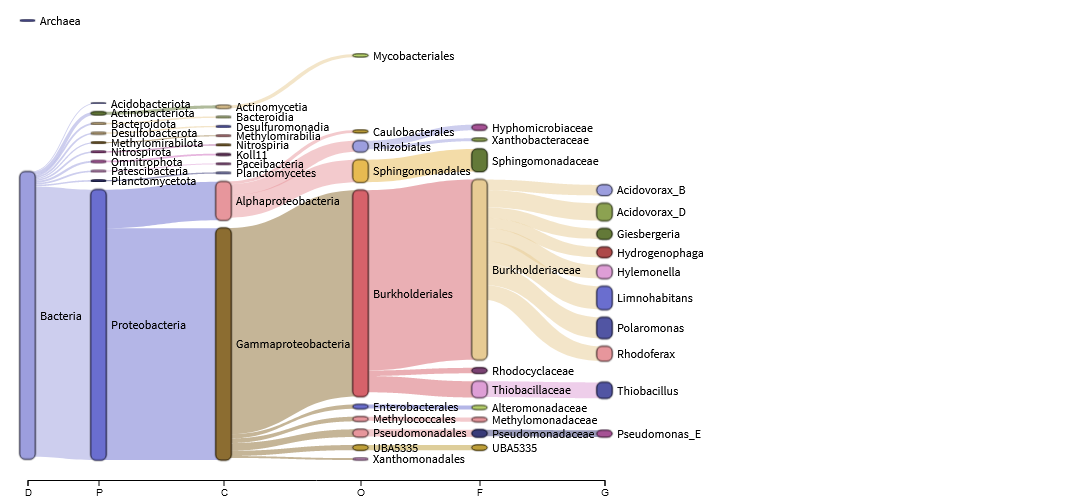


**Figure S5.** **Taxonomic profiling of Site E (CHL) microbiota obtained from shotgun metagenomic.** Different colored sidebars in the Sankey diagram show the relative abundance of the microbial community. Flow diagram represents the top 10 taxon at each taxonomic level. Lineage: D, Domain; K, kingdom; P, phylum; C, class; O, order; F, family; G, genus.


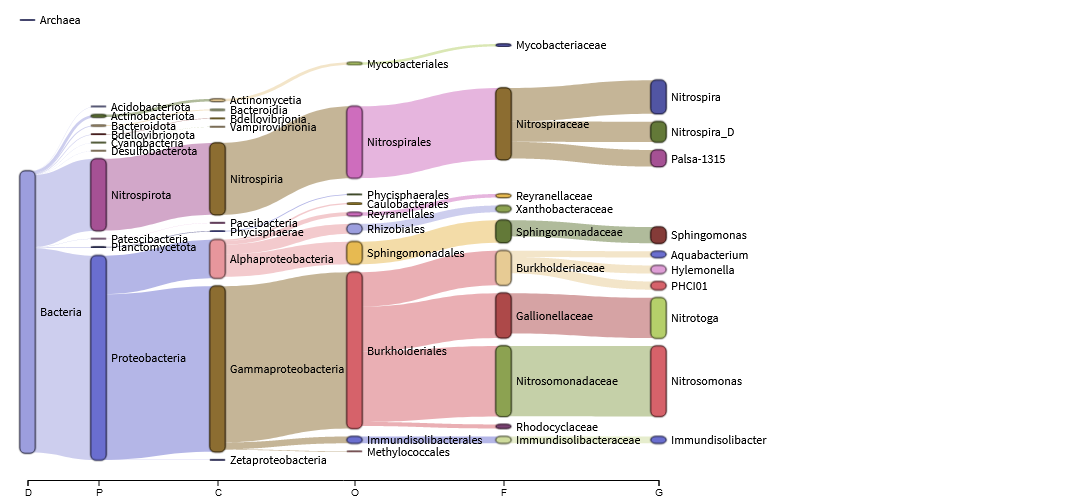


**Figure S6.** **Taxonomic profiling of Site D (CHM) microbiota obtained from shotgun metagenomic.** Different colored sidebars in the Sankey diagram show the relative abundance of the microbial community. Flow diagram represents the top 10 taxon at each taxonomic level. Lineage: D, Domain; K, kingdom; P, phylum; C, class; O, order; F, family; G, genus.


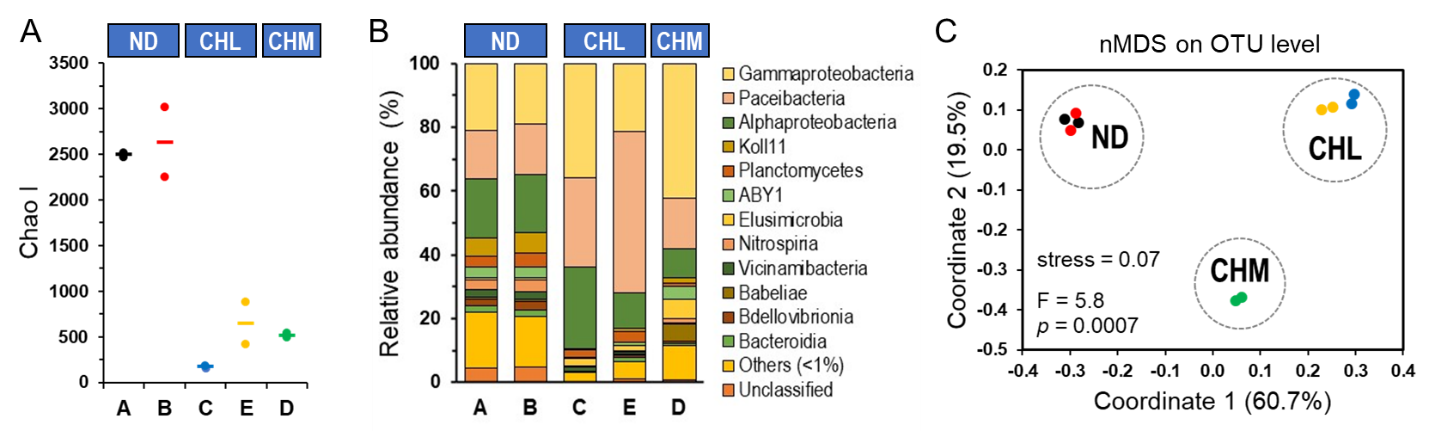


**Figure S7. Bacterial diversity and community composition based on the 16S rRNA profile of five DWDS.** (A) A decreasing gradient in microbial diversity revealed by Chao 1 from the non-disinfectant to the treated-water. (B) The bacterial relative abundance (%) of each DWDS at the class level. (C) nMDS of all samples based on Jensen-Shannon distance on OTU level. Disinfectant: ND, none; CHL, chlorine; CHM, chloramine.


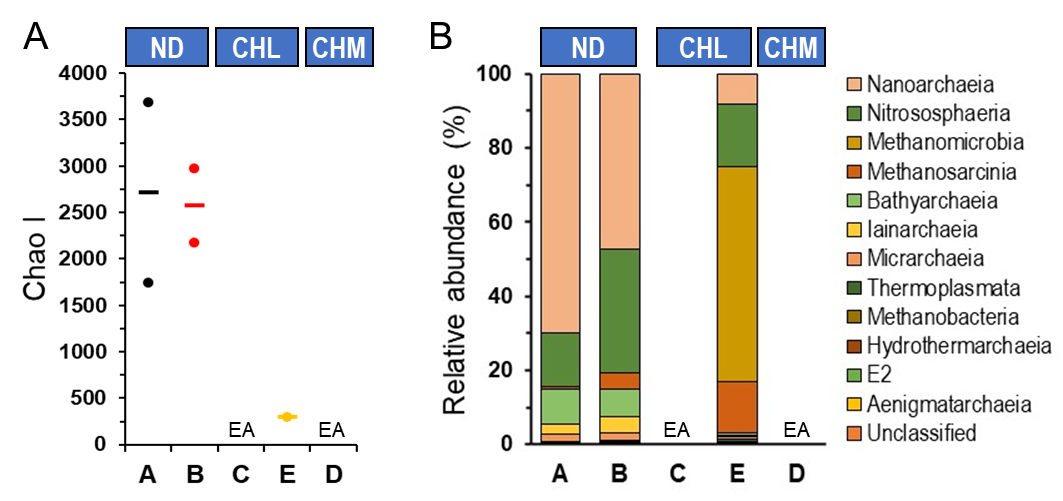


**Figure S8.** **Archaeal diversity and community composition based on the 16S rRNA profile of three DWDS.** (A) A decreasing gradient in microbial diversity revealed by Chao 1 from the non-disinfectant to the treated-water. (B) The archaeal relative abundance of each DWDS at the class level. Disinfectant: none, ND; chlorine, CHL. EA: excluded from analysis.


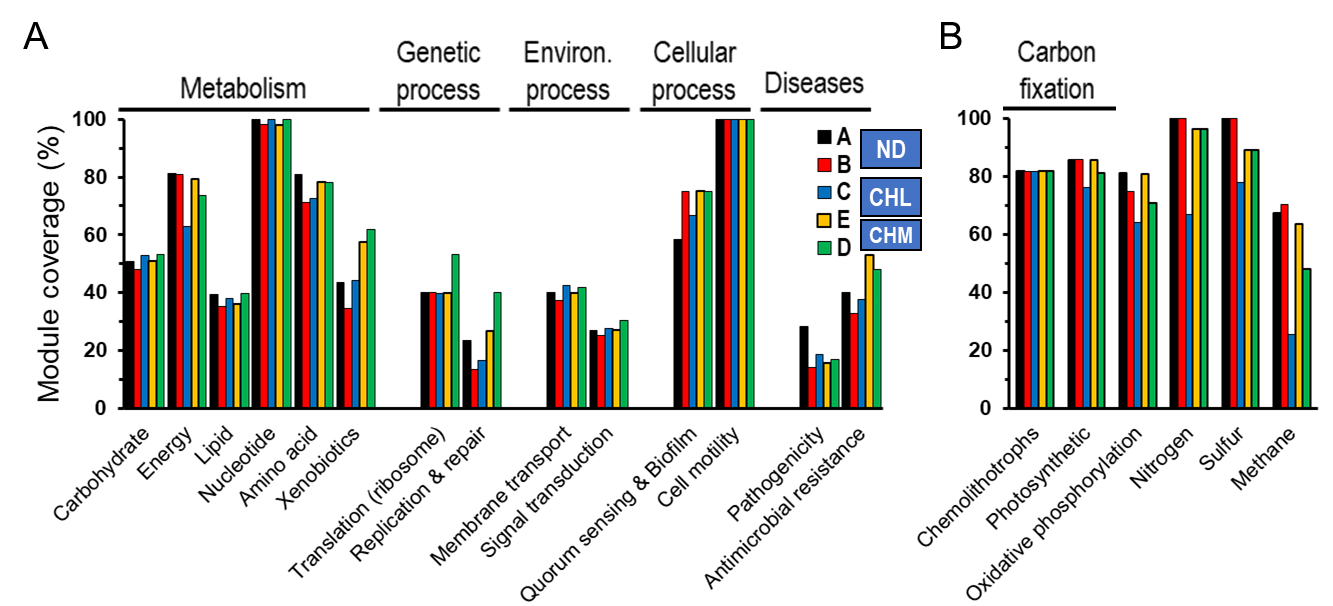


**Figure S9.** **Metabolic potential of five DWDS revealed by shotgun metagenomes.** Module coverage (%) estimated in KEGG (A) biological processes and (B) energy metabolic pathways. Disinfectant: ND, none; CHL, chlorine; CHM, chloramine.


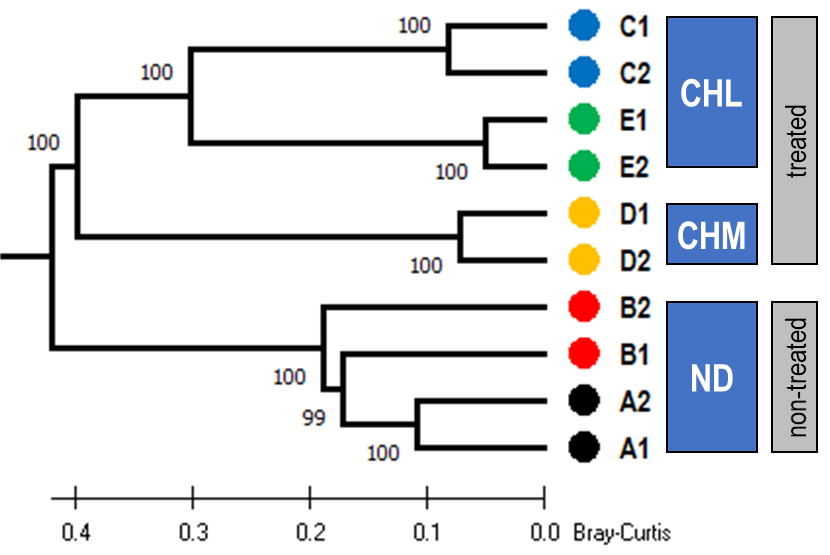


**Figure S10. Cluster analysis of metabolic profiles from five DWDS.** UPGMA cluster tree based on the Bray-Curtis matrix calculated from the relative abundance of KEGG orthologs (KO). Nodes with a bootstrap value ≥50% of 1,000 replicates are identified. The scale-bar represents Bray-Curtis dissimilarity distance. Disinfectant: ND, none; CHL, chlorine; CHM, chloramine.


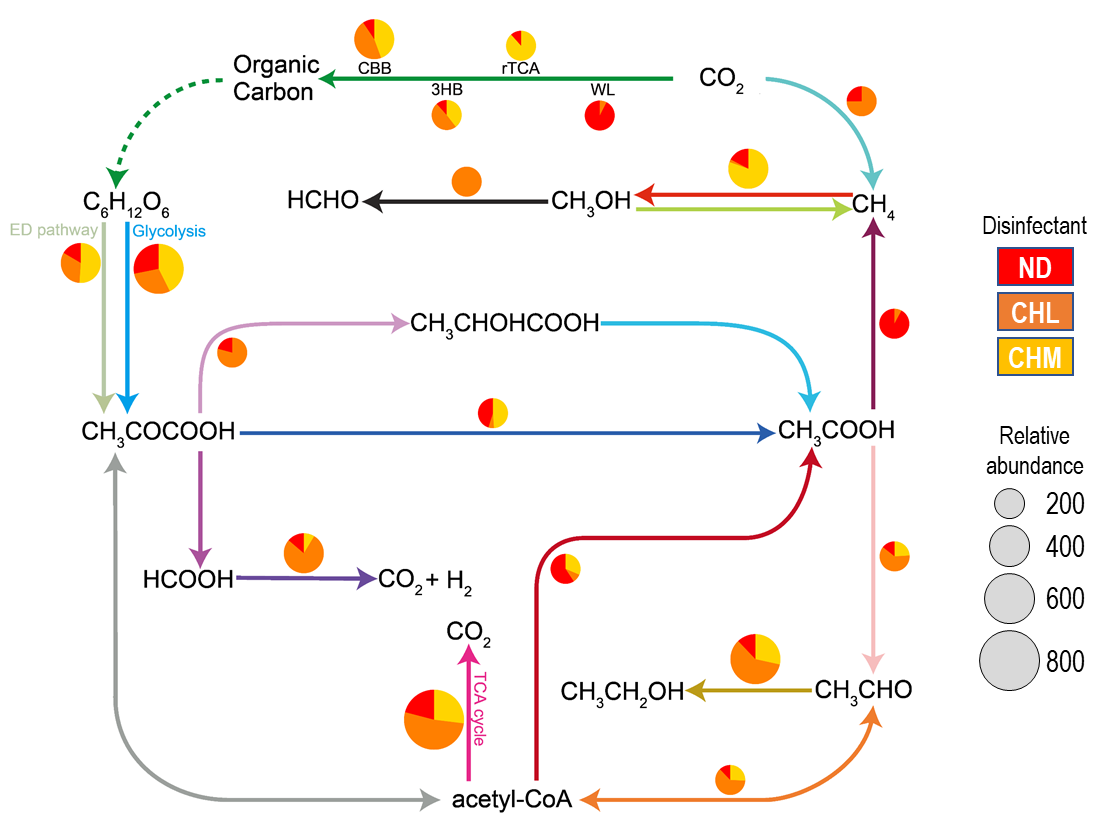


**Figure S11. Relative abundances of the pathways involved in the Carbon cycle.** The pie chart indicates the relative abundance of each pathway in each disinfectant group and the size of the pie chart represent the total relative abundance of each pathway. Disinfectant: ND, none; CHL, chlorine; CHM, chloramine. Pathways: CBB, Calvin-Benson-Bassham cycle; rTCA, reductive citric acid cycle; WL, Wood-Ljungdahl pathway; 3HB, 3-hydroxypropionate bicycle; ED, Entner-Doudoroff; TCA, tricarboxylic acid (Krebs cycle).


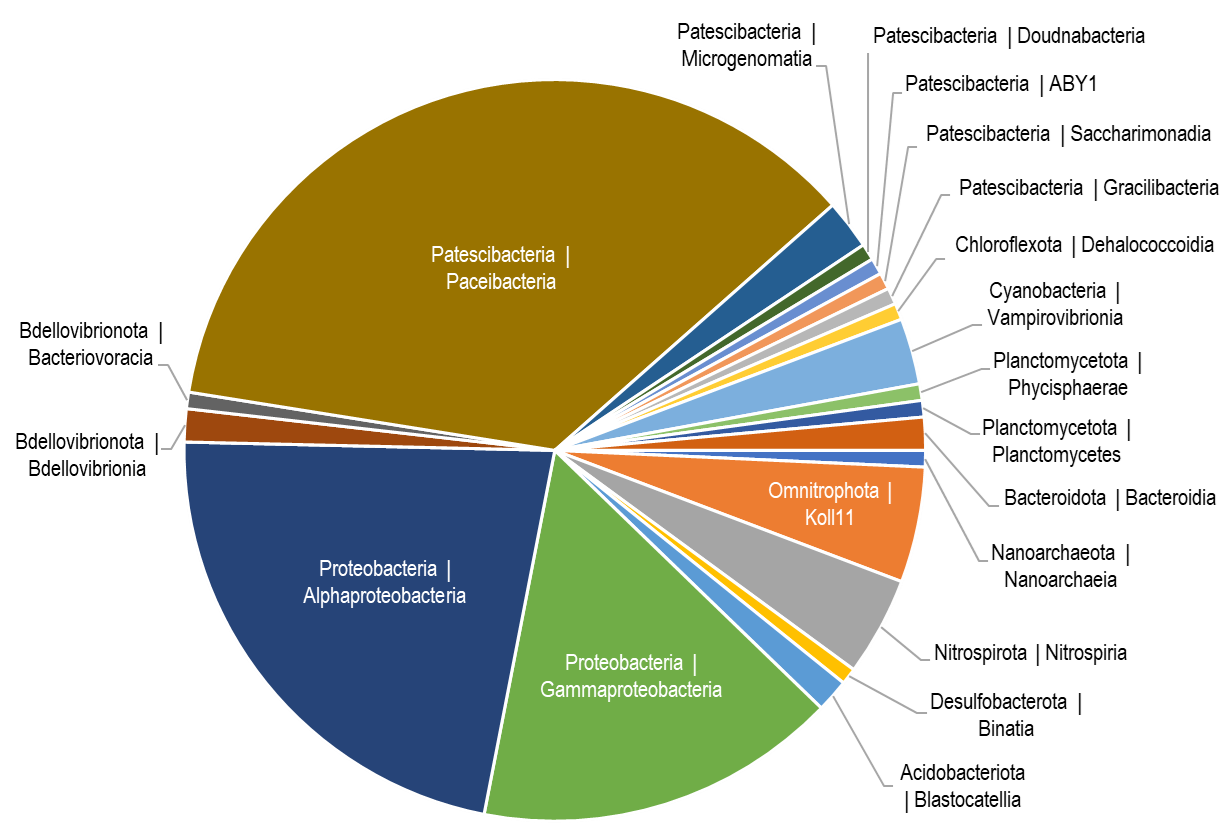


**Figure S12. Taxonomic distribution of MAGs.** Taxonomic classification based on phylum and class levels and abundance (%) of 139 recovered MAGs. Lineage: phylum | class.


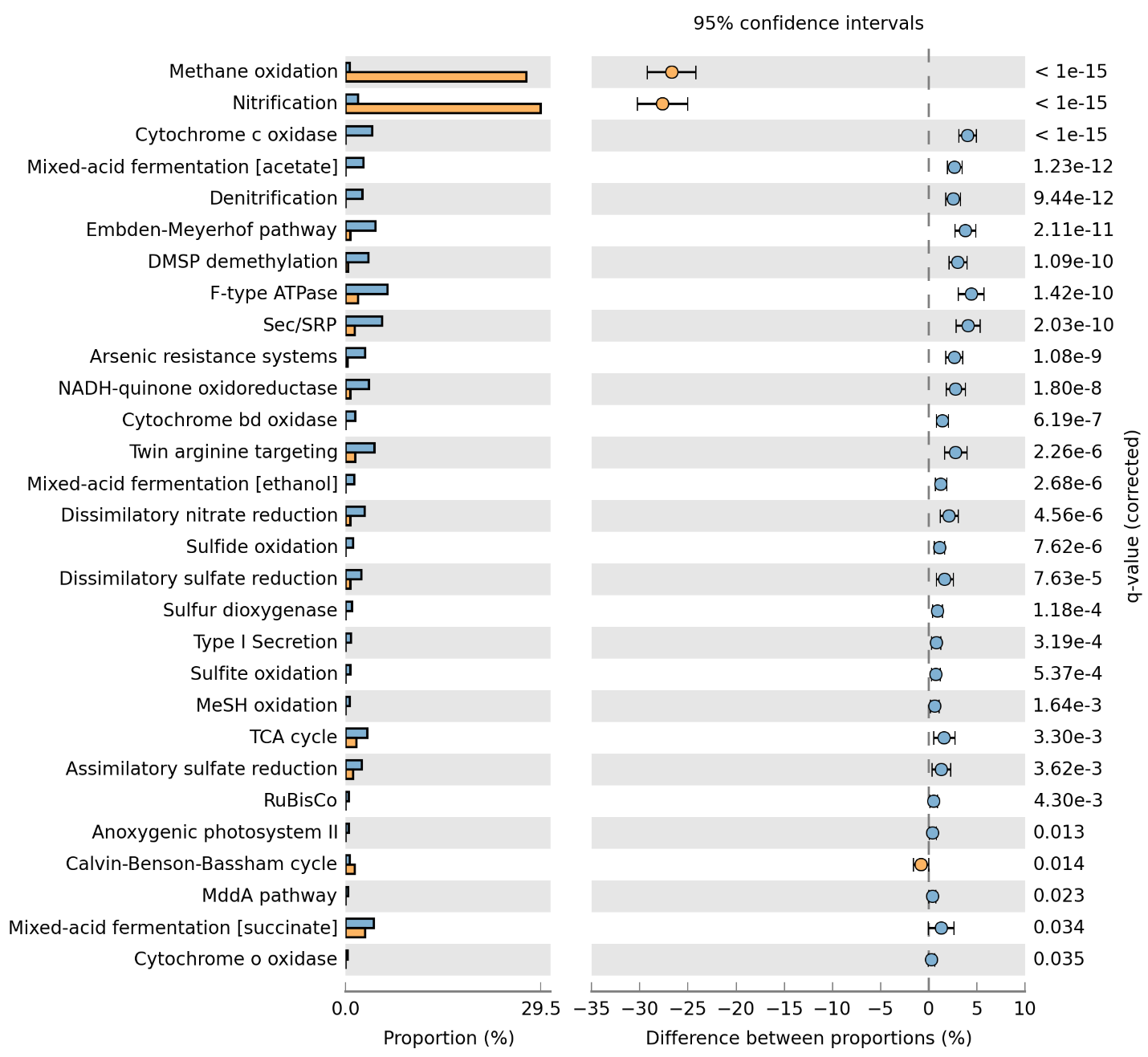


**Figure S13. Metabolic profiles of the total (■) and active (■) community for Site A (ND).** KEGG metabolism pathway differences (q-value < 0.05, Fisher’s t-test and Storey’s FDR multiple test correction) were identified.


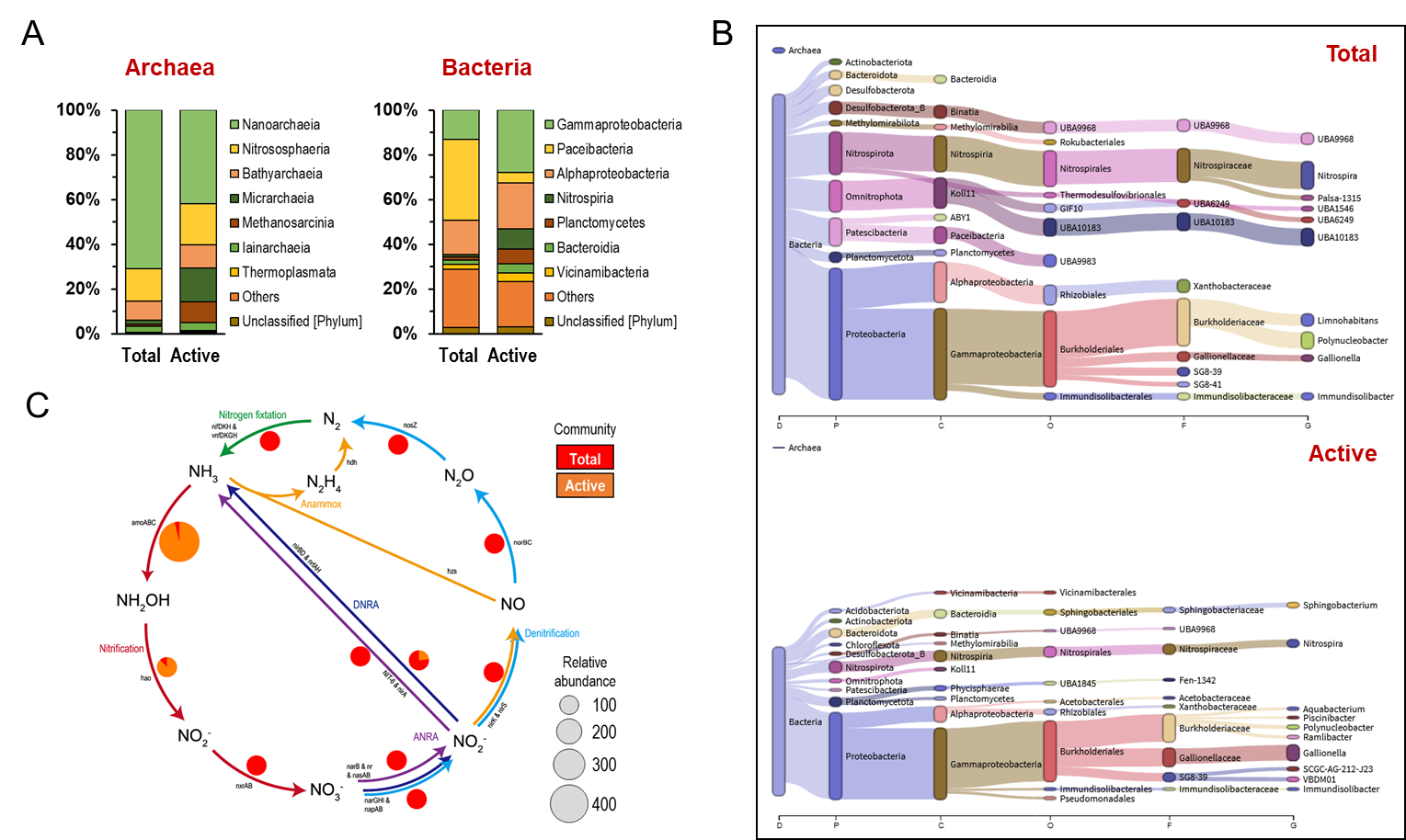


**Figure S14. Taxonomic and functional profiles of the total and active community for Site A (ND).** (A) Relative abundance (%) of Archaea and Bacteria classes based on 16S rRNA gene profiles. (B) Different colored sidebars in the Sankey diagram show the relative abundance of the total and active microbial communities based on metagenome and metatranscriptome sequencing. Flow diagrams represent the top 10 taxon at each taxonomic level. (C) Relative abundances of the pathways involved in the Nitrogen cycle. The pie chart indicates the relative abundance of each pathway in the total and active community and the size of the pie chart is proportional to the relative abundance of the gene involved in the pathway. Lineage: D, Domain; K, kingdom; P, phylum; C, class; O, order; F, family; G, genus. Nitrogen pathways: ANRA, assimilatory nitrate reduction to ammonium DNRA, Dissimilatory nitrate reduction to ammonium; Anammox, anaerobic ammonium oxidation.


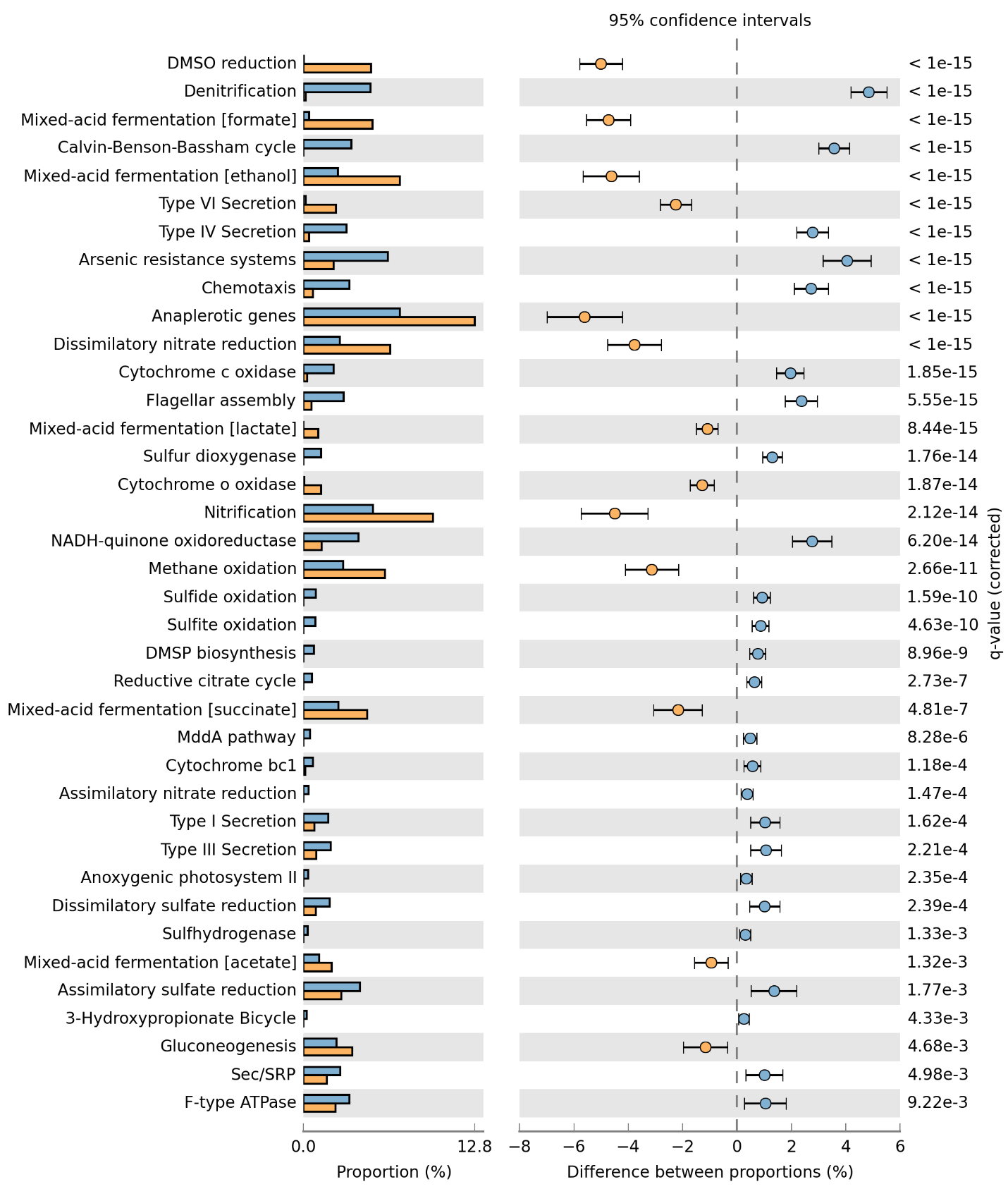


**Figure S15. Metabolic profiles of the total (■) and active (■) community for Site D (CHM).** KEGG metabolism pathway differences (q-value < 0.05, Fisher’s t-test and Storey’s FDR multiple test correction) were identified.


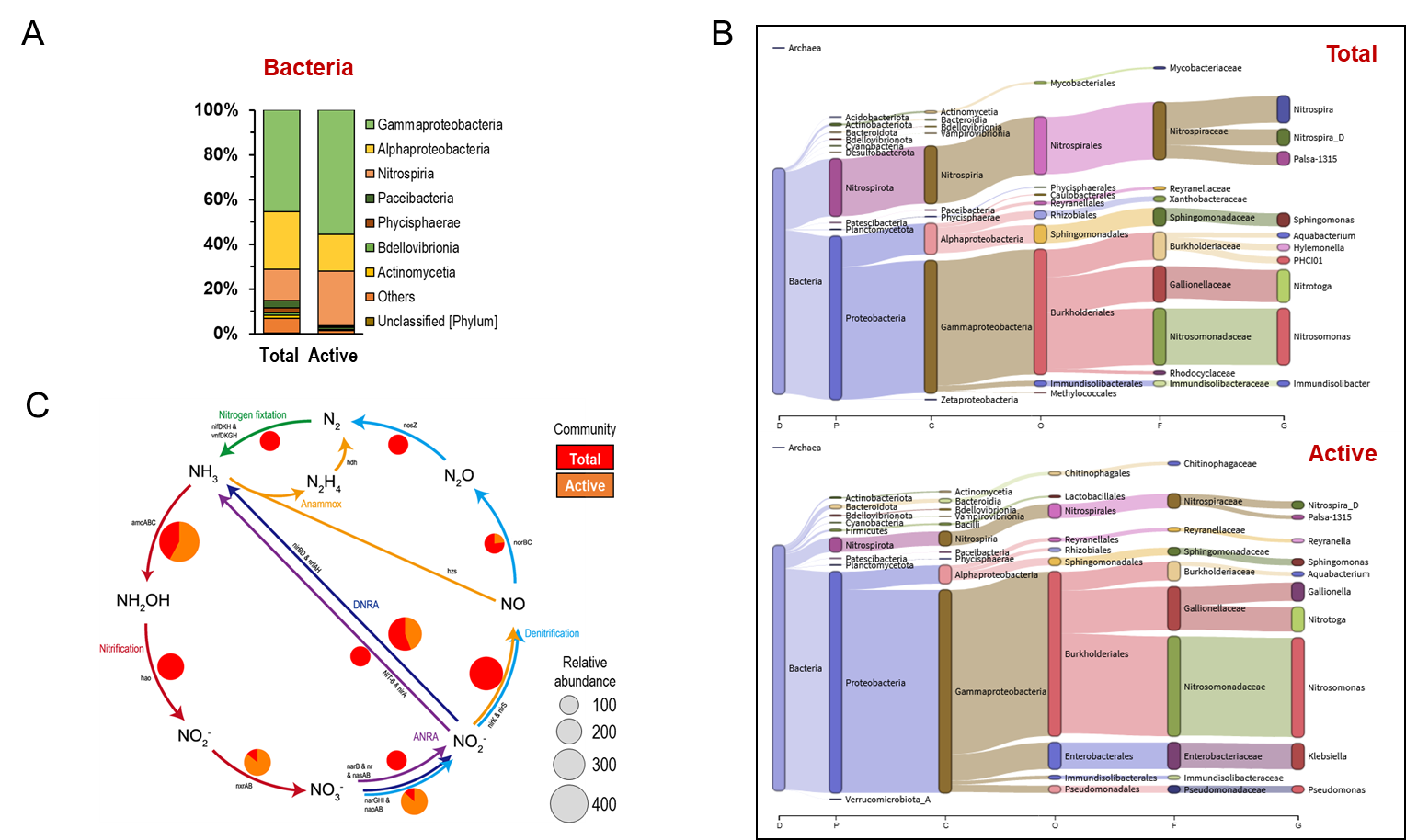


**Figure S16. Taxonomic and functional profiles of the total and active community for Site D (CHM).** (A) Relative abundance (%) of Archaea and Bacteria classes based on 16S rRNA gene profiles. (B) Different colored sidebars in the Sankey diagram show the relative abundance of the total and active microbial communities based on metagenome and metatranscriptome sequencing. Flow diagrams represent the top 10 taxon at each taxonomic level. (C) Relative abundances of the pathways involved in the Nitrogen cycle. The pie chart indicates the relative abundance of each pathway in the total and active community and the size of the pie chart is proportional to the relative abundance of the gene involved in the pathway. Lineage: D, Domain; K, kingdom; P, phylum; C, class; O, order; F, family; G, genus. Nitrogen pathways: ANRA, assimilatory nitrate reduction to ammonium DNRA, Dissimilatory nitrate reduction to ammonium; Anammox, anaerobic ammonium oxidation.


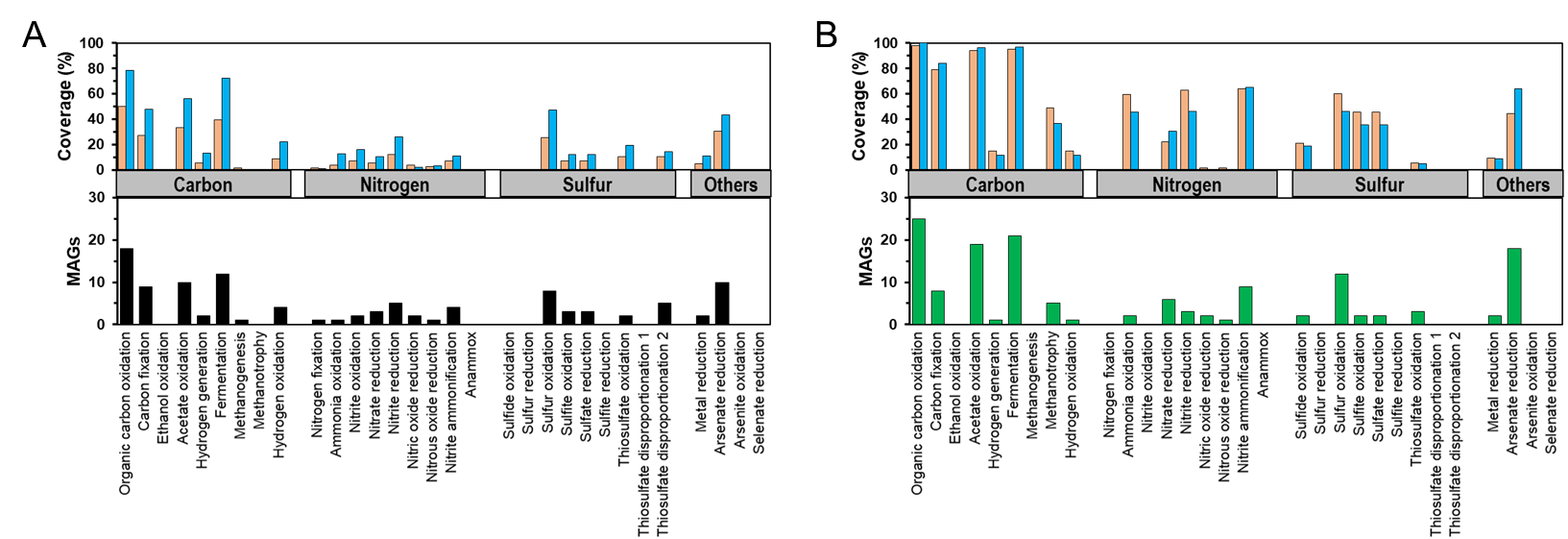


**Figure S17. Prediction of metabolic and biogeochemical functional traits in MAGs.** Number and coverage (%) of MAGs recovered from (A) Site A (■ ND) and (B) Site D (■ CHM). The lower graphs indicate the number of MAGs that have the genes present for that pathway. The top graphs represent the metagenome (■) and metatranscriptome (■) coverage for those MAGs in the total and active community, respectively. Disinfectant: ND, none; CHM, chloramine.

**REFERENCES**

Alishum, A. (2021). DADA2 formatted 16S rRNA gene sequences for both bacteria & archaea (Version Version 4.1) [Data set]. Zenodo. http://doi.org/10.5281/zenodo.4409439.

Aramaki, T., Blanc-Mathieu, R., Endo, H., Ohkubo, K., Kanehisa, M., Goto, S., Ogata H. 2019. KofamKOALA: KEGG ortholog assignment based on profile HMM and adaptive score threshold. Bioinformatics 7, 2251-2252. doi: 10.1093/bioinformatics/btz859.

Asnicar, F., Thomas A.M., Beghini, F., Mengoni C., Manara, S., Manghi, P., Zhu, Q., Bolzan, M., Cumbo, F., May, U., Sanders, J.G., Zolfo, M., Kopylova, E., Pasolli, E., Knight, R., Mirarab, S., Huttenhower, C., Segata, N. 2020. Precise phylogenetic analysis of microbial isolates and genomes from metagenomes using PhyloPhlAn 3.0. Nat. Commun. 11, 2500. doi: 10.1038/s41467-020-16366-7.

Bowers, R.M., Kyrpides, N.C., Stepanauskas, R., Harmon-Smith, M., Doud, D., Reddy, T.B.K., Schulz, F., Jarett, J., Rivers, A.R., Eloe-Fadrosh, E.A., Tringe, S.G, Ivanova, N.N., Copeland, A., Clum, A., Becraft, E.D., Malmstrom, R.R., Birren, B., Podar, M., Bork, P., Weinstock, G.M., Garrity, G.M., Dodsworth, J.A., Yooseph, S., Sutton, G., Glöckner, F.O., Gilbert, J.A., Nelson, W.C., Hallam, S.J., Jungbluth, S.P., Ettema, T.J.G., Tighe, S., Konstantinidis, K.T., Liu, W.-T., Baker, B.J., Rattei, T., Eisen, J.A., Hedlund, B., McMahon, K.D., Fierer, N., Knight, R.: Finn, R., Cochrane, G., Karsch-Mizrachi, I., Tyson, G.W., Rinke, C., The Genome Standards Consortium, Lapidus, A., Meyer, F., Yilmaz, P., Parks, D.H., Murat Eren, A., Schriml, L., Banfield, J.F., Hugenholtz, P., Woyke, T. 2017. Minimum information about a single amplified genome (MISAG) and a metagenome-assembled genome (MIMAG) of Bacteria and Archaea. Nat. Biotechnol. 35, 725-731. doi: 10.1038/nbt.3893.

Chaumeil, P.A., Mussig, A.J., Hugenholtz, P., Parks, D.H. 2019. GTDB-Tk: a toolkit to classify genomes with the Genome Taxonomy Database. Bioinformatics 36, 1925-1927. doi: 10.1093/bioinformatics/btz848.

Darzi, Y., Yamate, Y., Yamada, T. 2019. FuncTree2: an interactive radial tree for functional hierarchies and omics data visualization. Bioinformatics 35, 4519-4521. doi: 10.1093/bioinformatics/btz245.

Eddy, S.R. 2011. Accelerated Profile HMM Searches. PLoS Comput. Biol. 7, e1002195. doi: 10.1371/journal.pcbi.1002195.

Gantner, S., Andersson, A.F., Alonso-Sáez, L., Bertilsson, S., 2011. Novel primers for 16S rRNA-based archaeal community analyses in environmental samples. J. Microbiol. Methods 84, 12-18. doi:10.1016/j.mimet.2010.10.001.

Gomez-Alvarez, V., Pfaller, S., Pressman, J.G., Wahman, D.G., Revetta, R.P. 2016. Resilience of microbial communities in a simulated drinking water distribution system subjected to disturbances: role of conditionally rare taxa and potential implications for antibiotic-resistant bacteria. Environ. Sci.: Water Res. Technol. 2, 645-657. doi: 10.1039/c6ew00053c.

Hyatt, D., Chen, G., Locascio, P.F., Land, M.L., Larimer, F.W., Hauser, L.J. 2010. Prodigal: prokaryotic gene recognition and translation initiation site identification. BMC Bioinformatics 11, 119. doi: 10.1186/1471-2105-11-119.

Ikonen, J.M., Hokajärvi, A-M., Heikkinen, J., Pitkänen, T., Ciszek, R., Kolehmainen, M. 2017. Drinking water quality in distribution systems of surface and ground waterworks in Finland. J .Water Secur. 3, 1-10. doi: 10.15544/jws.2017.004.

Inkinen, J., Jayaprakash, B., Siponen, S., Hokajärvi, A., Pursiainen, A., Ikonen, J. 2019. Active eukaryotes in drinking water distribution systems of ground and surface waterworks. Microbiome 7, 1-17. doi:10.1186/s40168-019-0715-5.

Inkinen, J., Siponen, S., Jayaprakash, B., Tiwari, A., Hokajärvi, A.M., Pursiainen, A., Ikonen, J., Kauppinen, A., Miettinen, I.T., Paananen, J., Torvinen, E., Kolehmainen, M., Pitkänen, T. 2021 Diverse and active archaea communities occur in non-disinfected drinking water systems-Less activity revealed in disinfected and hot water systems. Water Res. X 12, 100101. doi: 10.1016/j.wroa.2021.100101.

Kang, D.D., Li, F., Kirton, E., Thomas, A., Egan. R., An, H., Wang, Z. 2019. MetaBAT 2: an adaptive binning algorithm for robust and efficient genome reconstruction from metagenome assemblies. PeerJ 7, e7359. doi: 10.7717/peerj.7359.

Klindworth, A., Pruesse, E., Schweer, T., Peplies, J., Quast, C., Horn, M., Glöckner, F.O. 2013. Evaluation of general 16S ribosomal RNA gene PCR primers for classical and next-generation sequencing-based diversity studies. Nucleic Acids Res. 41, e1. doi: 10.1093/nar/gks808.

Langmead, B., Salzberg, S.L. 2012. Fast gapped-read alignment with Bowtie 2. Nat. Methods 9, 357-359. doi: 10.1038/nmeth.1923.

Li, H., Handsaker, B., Wysoker, A., Fennell, T., Ruan, J., Homer, N., Marth, G., Abecasis, G., Durbin, R. 2009. 1000 Genome Project Data Processing Subgroup. The Sequence alignment/map (SAM) format and SAMtools. Bioinformatics 25, 2078-2079. doi: 10.1093/bioinformatics/btp352.

Nayfach, S., Shi, Z.J., Seshadri, R., Pollard, K.S., Kyrpides, N.C. 2019. New insights from uncultivated genomes of the global human gut microbiome. Nature 568, 505-510. doi: 10.1038/s41586-019-1058-x.

Olm, M.R., Brown, C.T., Brooks, B., Banfield, J.F. 2017. dRep: a tool for fast and accurate genomic comparisons that enables improved genome recovery from metagenomes through de-replication. ISME J. 11, 2864-2868. doi: 10.1038/ismej.2017.126.

Parks, D.H., Imelfort, M., Skennerton, C.T., Hugenholtz, P., Tyson, G.W. 2015. CheckM: assessing the quality of microbial genomes recovered from isolates, single cells, and metagenomes. Genome Res. 25, 1043-1055. doi: 10.1101/gr.186072.114.

Parks, D.H., Rinke, C., Chuvochina, M., Chaumeil, P.A., Woodcroft, B.J., Evans, P.N., Hugenholtz, P., Tyson, G.W. 2017. Recovery of nearly 8,000 metagenome-assembled genomes substantially expands the tree of life. Nat. Microbiol. 2, 1533-1542. doi: 10.1038/s41564-017-0012-7.

Parks, D.H., Chuvochina, M., Chaumeil, P.A., Rinke, C., Mussig, A.J., Hugenholtz, P. 2020. A complete domain-to-species taxonomy for Bacteria and Archaea. Nat. Biotechnol. 38, 1079-1086. doi: 10.1038/s41587-020-0501-8.

Quast, C., Pruesse, E., Yilmaz, P., Gerken, J., Schweer, T., Yarza, P., Peplies, J., Glöckner, F.O. 2013. The SILVA ribosomal RNA gene database project: improved data processing and web-based tools. Nucleic Acids Res. 41, D590-D596. doi: 10.1093/nar/gks1219.

Rambaut, A. 2018. FigTree v1.4.4. Available at: https://github.com/rambaut/figtree (accessed 18 February 2021).

Schloss, P.D., Westcott, S.L., Ryabin, T., Hall, J.R., Hartmann, M., Hollister, E.B., Lesniewski, R.A., Oakley, B.B., Parks, D.H., Robinson, C.J., Sahl, J.W., Stres, B., Thallinger, G.G., Van Horn, D.J., Weber, C.F. 2009. Introducing mothur: open-source, platform-independent, community-supported software for describing and comparing microbial communities. Appl. Environ. Microbiol. 75, 7537-7541. doi: 10.1128/AEM.01541-09.

Seemann, T. 2014. Prokka: rapid prokaryotic genome annotation. Bioinformatics 30, 2068-2069. doi: 10.1093/bioinformatics/btu153.

Sieber, C.M.K., Probst, A.J., Sharrar, A., Thomas, B.C., Hess, M., Tringe, S.G., Banfield, J.F. 2018. Recovery of genomes from metagenomes via a dereplication, aggregation and scoring strategy. Nat. Microbiol. 3, 836-843. doi: 10.1038/s41564-018-0171-1.

Stahl, D.A., Amann, R., 1991. Development and application of nucleic acid probes in bacterial systematics. In: Stackebrandt, E., Goodfellow, M. (Eds.), Nucleic Acid Techniques in Bacterial Systematics. John Wiley and Sons Ltd., Chichester, UK, pp. 205-248. doi:10.1002/jobm.3620310616.

Stamatakis A. 2014. RAxML version 8: a tool for phylogenetic analysis and post-analysis of large phylogenies. Bioinformatics 30, 1312-1313. doi: 10.1093/bioinformatics/btu033.

Uritskiy, G.V., DiRuggiero, J., Taylor, J. 2018. MetaWRAP-a flexible pipeline for genome-resolved metagenomic data analysis. Microbiome 6, 158. doi: 10.1186/s40168-018-0541-1.

Wu, Y-W., Simmons, B.A., Singer, S.W. 2016. MaxBin 2.0: an automated binning algorithm to recover genomes from multiple metagenomic datasets. Bioinformatics 32, 605-607, doi: 10.1093/bioinformatics/btv638.

Xue, C., Lin, H., Zhu, X., Liu, J., Zhang, Y., Rowley, G., Todd, J., Li, M., Zhang, X. 2021. DiTing: a pipeline to infer and compare biogeochemical pathways from metagenomic and metatranscriptomic data. Front. Microbiol. 12, 698286. doi:10.3389/fmicb.2021.698286.

Yuan, C., Lei, J., Cole, J., Sun, Y. 2015. Reconstructing 16S rRNA genes in metagenomic data. Bioinformatics 31, i35-i43. doi: 10.1093/bioinformatics/btv231.
